# Effects of Nutritional Supplements on Inflammatory Biomarkers in Overweight and Obese Adults: A Systematic Review and Network Meta-Analysis of Randomized Controlled Trials

**DOI:** 10.3390/nu18172936

**Published:** 2026-09-07

**Authors:** Zhiyue Chen, Ailing Liu, Shuran Bian, Zhize Zhou, Dong Zhang

**Affiliations:** Institute of Artificial Intelligence in Sports, Capital University of Physical Education and Sports, Beijing 100191, China; chelseaczy2025@163.com (Z.C.); 19506595698@163.com (A.L.); b2775830871@163.com (S.B.); zhouzhize@cupes.edu.cn (Z.Z.)

**Keywords:** obesity, overweight, nutritional supplements, inflammatory biomarkers, meta-analysis, randomized controlled trials

## Abstract

**Background/Objectives**: Obesity is frequently associated with chronic low-grade inflammation, which contributes to metabolic dysfunction. However, the comparative effects of nutritional supplements on inflammatory biomarkers in adults with overweight or obesity remain unclear. To address this gap, this paper presents a systematic review and network meta-analysis comparing 14 supplementation strategies for their effects on inflammatory biomarkers in this population. **Methods**: PubMed, Embase, EBSCO, Web of Science, and the Cochrane Library were searched for randomized controlled trials published between January 2000 and January 2026. A frequentist random-effects network meta-analysis was conducted. Effects were reported as mean differences (MDs) with 95% confidence intervals (CIs), and interventions were ranked using the surface under the cumulative ranking curve (SUCRA). The confidence in network estimates was assessed using the Confidence in Network Meta-Analysis (CINeMA) framework. **Results**: A total of 60 randomized controlled trials were included. Green tea extract (MD = −2.57, 95% CI [−3.99, −1.15]) and curcumin (MD = −1.75, 95% CI [−2.74, −0.76]) both significantly reduced IL-6 levels. Synbiotics (MD = −12.25, 95% CI [−16.7, −7.8]), curcumin (MD = −2.59, 95% CI [−3.83, −1.35]) and probiotics combined with omega-3 (MD = −6.19, 95% CI [−9.12, −3.26]) had a favorable effect on TNF-α. Probiotics combined with omega-3 (MD = −1.74, 95% CI [−3.03, −0.46]), synbiotics (MD = −1.19, 95% CI [−1.85, −0.53]), zinc (MD = −1.18, 95% CI [−2.24, −0.11]), and curcumin (MD = −1.11, 95% CI [−2.14, −0.07]) all reduced CRP levels. Synbiotics (MD = 15.37, 95% CI [12.13, 18.61]) increased adiponectin levels. In contrast, no nutritional supplement significantly reduced leptin levels. Exploratory meta-regression suggested that age, exercise co-interventions, and the proportion of male participants may partly explain heterogeneity in selected outcomes. **Conclusions**: Nutritional supplements showed biomarker-specific effects. Probiotics combined with omega-3 fatty acids, synbiotics, green tea extract, and curcumin may improve selected inflammatory biomarkers. However, differences in supplement dose, intervention duration, and participant characteristics warrant cautious interpretation and call for further high-quality trials.

## 1. Introduction

In recent years, obesity has become one of the most serious public health challenges worldwide. According to statistics from the World Health Organization (WHO), as of 2022, 2.5 billion adults aged 18 and over were overweight globally, including more than 890 million classified as obese [1]. The World Obesity Federation’s World Obesity Atlas 2025 projects that the number of adults living with obesity worldwide will increase by more than 115%, from 524 million in 2010 to 1.13 billion by 2030 [2]. Furthermore, it is predicted that by 2050, over half of the world’s adult population will be overweight or obese [3]. The global prevalence of overweight and obesity continues to rise, with implications that extend far beyond increased body weight, as these conditions are closely associated with a higher risk of developing various chronic metabolic diseases.

Obesity leads to a state of low-grade chronic inflammation characterized by the upregulation of pro-inflammatory markers and the downregulation of anti-inflammatory cytokines. In individuals with obesity, adipose tissue exhibits dysregulated secretion of inflammation-related cytokines, including increased interleukin-6 (IL-6), tumor necrosis factor-alpha (TNF-α) and leptin, and decreased adiponectin, which likely contribute to a pro-inflammatory state and oxidative stress [4]. Elevated IL-6, in turn, stimulates hepatic synthesis of *C*-reactive protein (CRP), making it a crucial biomarker for assessing low-grade systemic inflammation [5]. These adipose-derived inflammatory biomarkers act through multiple signaling pathways to disrupt metabolic homeostasis, establishing a state of chronic, systemic low-grade inflammation that represents a central pathophysiological mechanism linking obesity to insulin resistance, type 2 diabetes, cardiovascular disease, and certain cancers [6,7,8,9]. Research by Hotamisligil et al. indicates that obesity-related inflammatory responses play a pivotal role in the development of metabolic diseases; therefore, reducing inflammation may be a key strategy for mitigating obesity-related health risks [10,11].

Lifestyle changes and dietary management are the safest and most cost-effective interventions for weight control in adults with obese or overweight diagnoses [12]. Nutritional supplements, as a dietary management strategy, have attracted widespread attention due to their greater safety profile compared to pharmaceuticals, as well as their ease of use and potential anti-inflammatory effects. In recent years, numerous randomized controlled trials (RCTs) have investigated the effects of various nutritional supplements on inflammatory biomarkers. The trace element zinc and the macromineral magnesium, as well as supplementation involving these elements, have emerged as key potential targets for modulating obesity-related inflammatory responses [13,14,15]. Omega-3 polyunsaturated fatty acids are thought to reduce inflammatory responses by inhibiting the NF-κB signaling pathway, thereby effectively lowering high-sensitivity *C*-reactive protein levels in individuals who are overweight or have central obesity [16]. Probiotic intervention can significantly influence the secretion levels of leptin and adiponectin by reshaping the gut microbiota, enhancing intestinal barrier function and modulating immune and inflammatory responses, thereby improving insulin resistance and lipid metabolism disorders [17,18]. As key micronutrients, vitamin D and vitamin E play important roles in alleviating obesity-related inflammation through immune-endocrine regulation and antioxidant defence mechanisms, respectively [19,20]. Furthermore, plant-derived polyphenols such as curcumin and resveratrol possess significant antioxidant and anti-inflammatory properties, reducing inflammation in adipose tissue by modulating gut microbiota and zinc homeostasis [21,22].

Although a large number of studies have investigated the anti-inflammatory effects of nutritional supplements, current research findings remain insufficient for drawing comparative conclusions. Previous meta-analyses have typically evaluated only a single type of nutritional supplement, making it difficult to compare the relative effects of different supplements. In clinical and public health practice, however, determining the differences in efficacy among various interventions and their relative prioritization is essential. Therefore, there is a need for a method capable of comparing multiple interventions simultaneously. Network meta-analysis (NMA) is a statistical method developed from traditional meta-analysis that integrates direct and indirect evidence within a single analytical framework, thereby enabling comparisons between multiple interventions and the ranking of their effectiveness. Compared with traditional meta-analysis, network meta-analysis provides more comprehensive evidence, offering a more reliable basis for public health interventions. Accordingly, this systematic review and network meta-analysis aimed to compare the effects of 14 pre-specified nutritional supplementation strategies on circulating inflammatory biomarkers in adults with overweight or obesity. Eligible studies were randomized controlled trials comparing these interventions with placebo, no supplementation, or one another. The primary outcomes were CRP, IL-6, and TNF-α, while leptin and adiponectin were evaluated as secondary outcomes. By integrating both direct and indirect evidence, this study sought to determine the relative efficacy of these supplementation strategies and to inform the development of future nutritional interventions targeting obesity-related inflammation.

## 2. Methods

### 2.1. Registration

This study was conducted in accordance with the PRISMA 2020 guidelines for reporting systematic reviews and meta-analyses [23], as well as the PRISMA-NMA guidelines for network meta-analyses [24]. The completed PRISMA-NMA checklist is provided in Supplementary File S1. The study protocol has been registered with and approved by the International Prospective Registration Centre for Systematic Reviews (PROSPEROID: CRD420261348823).

### 2.2. Search Strategy

A systematic search was conducted in the PubMed, Embase, EBSCO, Web of Science and Cochrane Library databases to identify all randomized controlled trials (RCTs) published in English from January 2000 to January 2026. The search strategy was formulated based on key phrases related to the components of the PICOS tool: (P) Population: overweight and obese adults; (I) Intervention: resveratrol, magnesium (Mg), vitamin D (VD), zinc, probiotics, omega-3, α-lipoic acid (ALA), synbiotics, vitamin E (VE), green tea extract, curcumin, vitamin D plus magnesium, probiotics plus ALA, and probiotics plus omega-3; (C) Control: no nutritional intervention or placebo; (O) Outcomes: TNF-α, IL-6, CRP, leptin, and adiponectin; (S) Study type: RCT. In addition to the databases, the reference lists of relevant articles and reviews were searched to avoid any potentially relevant studies that might have been overlooked by the electronic search. The full search strategies for each database are provided in Supplementary File S2.

### 2.3. Inclusion and Exclusion Criteria

The article reviews and selection process strictly adhered to the updated PRISMA guidelines. Based on the inclusion and exclusion criteria provided below, two reviewers conducted a comprehensive screening of the database search results by reviewing titles and abstracts to identify all potentially relevant studies. The full texts of research articles meeting the inclusion criteria were identified and independently assessed by the same two reviewers. Where any discrepancies or disagreements arose between the reviewers regarding the inclusion or exclusion of literature, consensus was reached through discussion or by seeking the opinion of a third-party researcher. Eligible studies met the following criteria: (1) Randomized controlled trials were published in English. (2) Studies were required to include at least one intervention group receiving nutritional supplementation for a minimum of 2 weeks. Eligible comparators included placebo, no supplementation, usual care, or another eligible supplementation strategy. Trials evaluating single supplements, combined supplementation strategies, and multi-arm interventions were eligible, provided that they met the predefined inclusion criteria and contributed to direct or indirect comparisons within the network. The minimum duration of 2 weeks was selected to exclude acute or single-dose supplementation effects and to ensure that the intervention represented a sustained supplementation period. Because intervention durations varied substantially across eligible studies (2–52 weeks), intervention duration was additionally examined as a potential effect modifier in the meta-regression analyses. (3) Participants were adults aged 18 years or older who met the criteria for overweight or obesity, defined as a body mass index (BMI) of ≥25 kg/m^2^ (overweight) or ≥30 kg/m^2^ (obesity) according to WHO (Western) criteria, or ≥24 kg/m^2^ (overweight) or ≥28 kg/m^2^ (obesity) according to Asian criteria. (4) Inflammatory biomarkers (IL-6, TNF-α, CRP, leptin or adiponectin) were assessed as outcomes before and after the dietary supplement intervention. Studies meeting any of the following criteria were excluded from the analysis. The exclusion criteria are as follows: (1) duplicate publications, letters to the editor, reviews, case reports, conference abstracts, animal studies, or other non-original research; (2) studies evaluating acute or single-dose supplementation effects; (3) studies in which dietary or exercise co-interventions differed between randomized groups; (4) studies with insufficient information on participant characteristics, intervention details, or study design; and (5) studies for which the full text or relevant outcome data could not be obtained despite attempts to contact the authors.

### 2.4. Data Extraction

Two researchers independently carried out the literature screening and data extraction, using a standardized data extraction form to record the findings. Data on changes before and after the intervention were extracted for this NMA. All data reported in the articles were continuous, and means and standard deviations (SD) were extracted. For studies reporting medians and interquartile ranges, means and standard deviations were estimated from the sample size, median, first quartile, and third quartile using the method proposed by Wan et al. [25]. For studies reporting 95% confidence intervals (CI), the standard error (SE) was estimated as (upper limit − lower limit)/3.92, and the standard deviation (SD) was calculated as SE × √n [26]. Where data were incomplete or unreported, or the full text was unavailable, we contacted the authors to request detailed outcome information.

In the event of disagreement, this was resolved through discussion or third-party arbitration. Information on study design was extracted, including study-level characteristics (i.e., first author’s name, year of publication and geographical location), participant-level characteristics (i.e., age, proportion of male participants and dietary control or regular exercise), and protocol-level characteristics (i.e., study design, sample size per group, type and dose of nutritional supplements, and outcome data). Pre-intervention and post-intervention change data were collected to conduct this NMA. For randomized controlled trials with multiple time points, only the final time point was considered, while intermediate time points were disregarded.

### 2.5. Assessment of Risk of Bias

The Cochrane Risk of Bias Assessment Tool was used to evaluate the quality of the included studies [27]. Using the Cochrane Risk of Bias Assessment Tool, two assessors independently assessed the risk of bias in the included studies (ROB 2.0). Any discrepancies were resolved through discussion to reach a consensus or by consulting a third party for a final decision. The Cochrane ROB 2 assessment tool comprises five domains: (1) the randomization process, (2) deviations from the intended intervention, (3) missing outcome data, (4) outcome measurement, and (5) selective reporting bias. For each domain of bias, studies were assessed based on the available information and categorized as ‘Low risk’, ‘High risk’ or ‘Some concerns.

### 2.6. Statistical Analysis

To minimize the impact of baseline differences on the estimation of intervention effects, effect sizes were calculated using the mean and standard deviation (SD) of change scores between baseline and post-intervention measurements. When the SD of the change score was not directly reported, it was calculated using the formula recommended in the Cochrane Handbook for Systematic Reviews of Interventions. Because study-specific correlations between baseline and post-intervention measurements were generally not reported in the included trials and therefore could not be empirically estimated, a common pre–post correlation coefficient of 0.50 was assumed and applied consistently across studies requiring estimation of change-score SDs. In accordance with the PRISMA guidelines for network meta-analyses, effect sizes were pooled using a random-effects model within frequentist random-effects network meta-analysis. The 95% confidence intervals (CIs) were calculated using Stata 17.0 software. Where necessary, biomarker concentrations were converted to common units: mg/L for CRP, pg/mL for IL-6 and TNF-α, and ng/mL for leptin and adiponectin. As each outcome was reported in a consistent unit across studies, effect estimates were pooled as mean differences (MDs) with 95% confidence intervals (CIs). Meta-regression analysis was then performed using Stata 17.0 to assess the impact of other factors on non-negligible differences in participants’ baseline characteristics [28]. The relationships among different nutritional supplement interventions were depicted using a network diagram, where lines connecting nodes represent direct comparisons between interventions. The thickness of these lines is proportional to the number of studies, and the size of the nodes is proportional to the sample size. The transitivity assumption was assessed qualitatively by comparing the distribution of clinically relevant potential effect modifiers across the available treatment comparisons. These included participants’ age, sex distribution, BMI, underlying comorbidities, baseline inflammatory status where reported, intervention duration, supplement dose, and concomitant lifestyle interventions such as exercise or dietary control. Because these characteristics were not uniformly reported across all trials, a formal statistical test of transitivity was not considered feasible. Instead, the plausibility of transitivity was evaluated on the basis of whether major clinical or methodological differences that could modify treatment effects were evident across the different comparisons.

Consistency within each closed loop was assessed by calculating the inconsistency factor and its 95% confidence interval (95% CI). An inconsistency model was used to test for inconsistency, and when the inconsistency was not statistically significant (*p* > 0.05), a consistency model was subsequently applied. Node-splitting analysis was used to test the consistency between direct and indirect comparisons, with *p* > 0.05 indicating non-significant inconsistency [29]. The surface under the cumulative ranking curve (SUCRA), based on cumulative probability plots, was used to rank and compare the intervention effects of different types of nutritional supplements [30]. SUCRA values range from 0 to 100; a higher value indicates that the intervention ranks higher in terms of effectiveness. The symmetry of the funnel plot was examined to determine whether publication bias or small-sample effects were present in the network meta-analysis (NMA). It is important to note that SUCRA values reflect only the relative ranking of interventions and do not represent the quality of evidence or clinical priority.

### 2.7. Certainty of Evidence

Confidence in the network meta-analysis estimates was assessed using the Confidence in Network Meta-Analysis (CINeMA) framework. The assessment considered six domains: within-study bias, reporting bias, indirectness, imprecision, heterogeneity, and incoherence. Within-study bias was informed by the RoB 2 assessments of the included randomized controlled trials, whereas indirectness was evaluated by considering the applicability of study populations and intervention characteristics to the review question, including participant comorbidities, BMI, intervention duration, supplement dose, and concomitant lifestyle interventions. Imprecision and heterogeneity were evaluated according to the magnitude and uncertainty of the network estimates, including confidence intervals and prediction intervals, in relation to the prespecified clinically important effect thresholds. Incoherence was assessed by examining agreement between direct and indirect evidence where such comparisons were estimable. Reporting bias was evaluated considering the comprehensiveness of the literature search and potential evidence of selective publication or small-study effects.

For each domain, concerns were categorized according to the CINeMA framework, and the overall confidence in each network estimate was rated as high, moderate, low, or very low. The certainty assessment focused primarily on comparisons between each nutritional supplementation strategy and placebo/control, as these comparisons constituted the principal basis for interpretation of intervention effects in this review. Detailed CINeMA assessments are presented in Supplementary Table S6.

### 2.8. Software

All statistical analyses were performed using Stata 17.0.

## 3. Results

### 3.1. Literature Search

**Figure 1:**
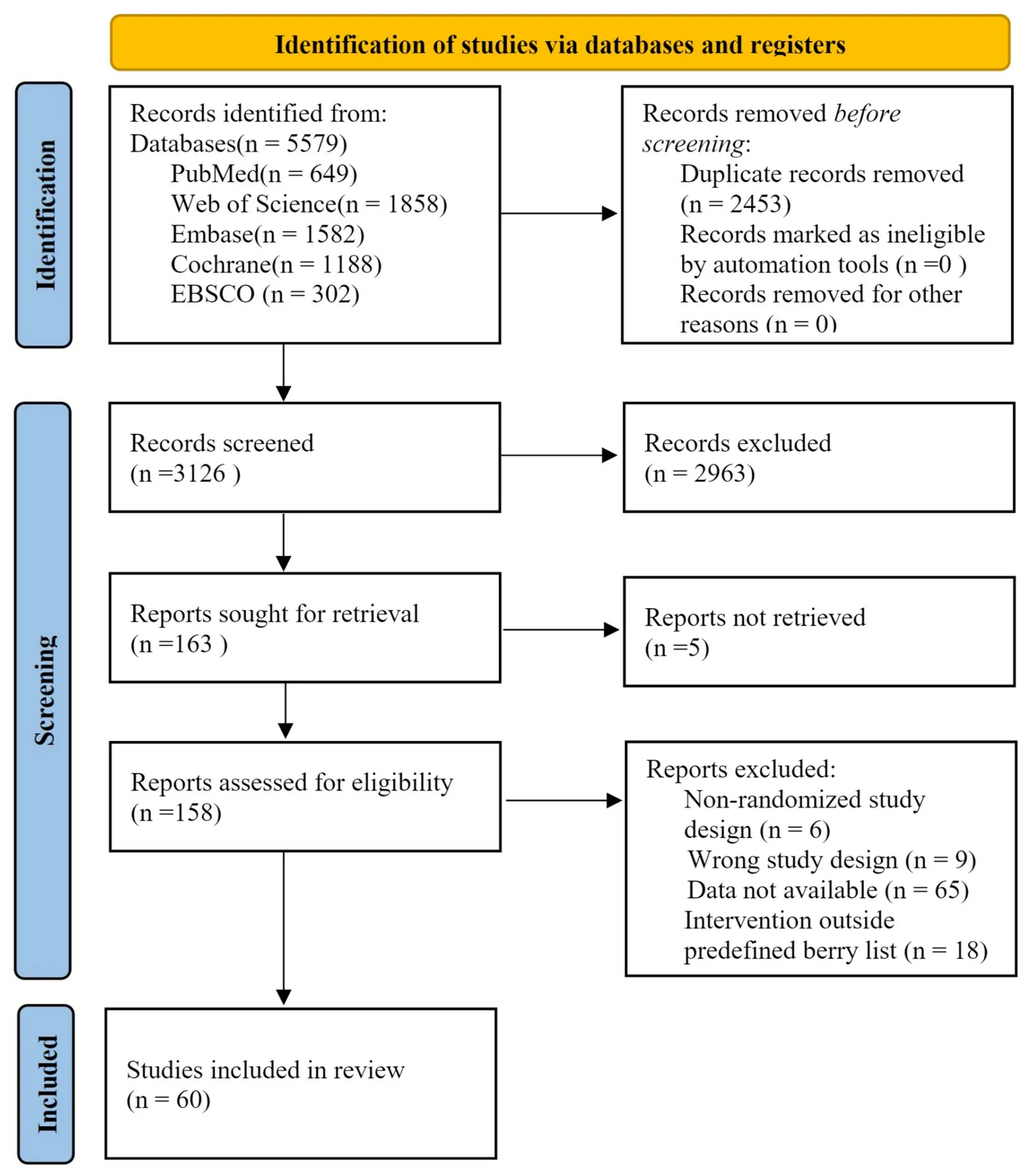
PRISMA flow diagram of the study selection process.

A total of 5579 articles were identified through the literature search. The search results were subsequently deduplicated, with 2453 duplicate articles removed, leaving a final total of 3126 articles for screening. During the initial screening of titles and abstracts, a total of 3126 articles were reviewed; 2963 articles unrelated to the research topic were excluded, leaving 163 articles proceeding to the full-text retrieval stage. Of these, 5 articles were excluded due to unavailability of the full text, resulting in 158 articles undergoing full-text assessment. During the full-text assessment, a total of 98 articles were excluded: 6 were non-randomized controlled trials; 9 had study designs that did not meet the inclusion criteria; 65 contained incomplete or non-extractable data; and 18 had interventions that did not meet the predefined criteria. Following this rigorous screening, 60 studies were ultimately included in the review for quantitative synthesis. A complete list of the included studies is provided in Supplementary File S3. The flowchart detailing the trial selection process is shown in Figure 1.

### 3.2. Characteristics of the Included Studies

All included studies were English-language randomized controlled trials published between January 2000 and January 2026. The majority of these studies were conducted in Iran (*n* = 19) and the United States (*n* = 9). Regarding study participants, 16 studies exclusively involved women, 8 exclusively involved men, 32 included both men and women, and 4 studies did not report the gender of participants or gender ratio. Baseline demographic characteristics reported included body mass index and body fat percentage. Participants in 25 studies had comorbidities such as diabetes, metabolic syndrome, polycystic ovary syndrome, and non-alcoholic steatohepatitis.

In terms of nutritional supplements, this study included 14 types: resveratrol, magnesium (Mg), vitamin D (VD), vitamin D combined with magnesium (VD + Mg), zinc, probiotics, probiotics combined with alpha-lipoic acid (ALA), probiotics combined with omega-3, alpha-lipoic, synbiotics, omega-3, vitamin E, green tea extract, curcumin. The duration of the nutritional interventions ranged from 2 to 52 weeks, with most studies implementing intervention periods of 8 or 12 weeks.

The outcome measures reported in the studies were as follows: more than half of the studies measured CRP (44 studies, 1227 participants in the intervention group and 1525 in the control group), IL-6 (36 studies, 1163 participants in the intervention group and 1327 in the control group), TNF-α (29 studies, 929 participants in the intervention group and 1050 in the control group); amongst adipokine-related markers, 11 studies measured leptin (303 participants in the intervention group and 279 in the control group), whilst 18 studies measured adiponectin (494 participants in the intervention group and 587 in the control group).

### 3.3. Results of the Risk of Bias Assessment

Detailed study-level risk-of-bias assessments are presented in Supplementary Table S4, and the corresponding risk-of-bias graph is presented in Supplementary Figure S2. The quality of the 60 included randomized controlled trials was assessed using the Revised Cochrane Risk of Bias Tool (ROB 2).

Regarding the randomization process, all 59 eligible randomized controlled trials mentioned randomization and were classified as ‘Low risk’; only one randomized controlled trial showed ‘Some concerns’; regarding deviation from the intended intervention, 3 studies showed ‘Some concerns’ and 2 studies showed ‘High risk’; regarding missing outcome data, 10 studies showed ‘Some concerns’, whilst the remaining studies were classified as ‘Low risk’; regarding outcome measurement, 7 studies showed ‘Some concerns’ and 1 study showed ‘High risk’; regarding selective reporting, all 60 studies were assessed as ‘Low risk’ for completeness of reporting.

### 3.4. Network Meta-Analysis

**Figure 2:**
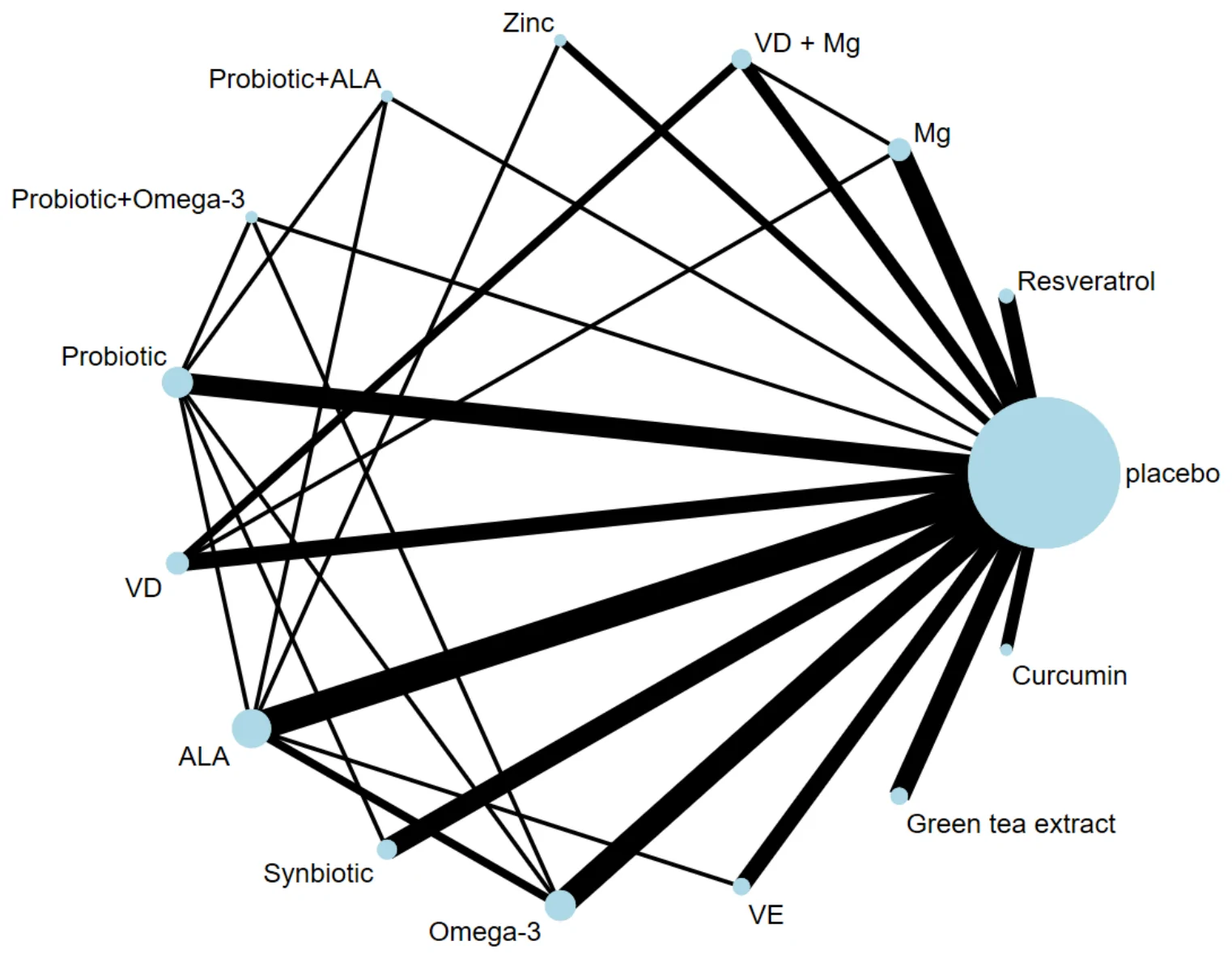
Network plots of eligible nutritional supplementation strategies for inflammatory biomarkers in adults with overweight or obesity.

Figure 2 shows the network meta-analysis diagram for studies that satisfied the inclusion criteria, illustrating the effects of different types of nutritional supplements on inflammatory biomarkers. The size of the nodes is proportional to the sample size of the studies corresponding to each type of nutritional supplement, whilst the thickness of the lines connecting different types of nutritional supplements represents the number of studies conducting direct comparisons within that category. Of all intervention types, probiotics were the most extensively studied, whilst zinc was the least studied. The corresponding supplementary network plot is provided in Supplementary Figure S1.

The clinical and methodological characteristics of the included studies were also reviewed to assess the plausibility of the transitivity assumption. Potential effect modifiers, including age, sex distribution, BMI, underlying comorbidities, intervention duration, supplement dose, and concomitant lifestyle interventions, varied across the included comparisons. In particular, a substantial proportion of trials included participants with metabolic or other chronic comorbidities, and intervention duration ranged from 2 to 52 weeks. These differences indicate the presence of clinical heterogeneity across the network. However, no single effect modifier was identified as being uniquely restricted to one treatment comparison on the basis of the available study-level information. Therefore, transitivity was considered broadly plausible, although it could not be fully verified because of incomplete reporting and heterogeneity in several potential effect modifiers. Accordingly, indirect comparisons were interpreted with caution.

To evaluate inconsistency in the analyses of inflammatory biomarkers, this study performed ring-specific heterogeneity estimates, inconsistency model analyses, and node decomposition analysis (Supplementary Table S3).

Supplementary Figure S3 shows funnel plots for inflammatory biomarkers, which were used to evaluate potential publication bias within the network meta-analysis. The funnel plots for all outcome measures demonstrated a generally symmetrical distribution, suggesting a low probability of publication bias or small-sample effects in the network meta-analysis conducted in this study.

### 3.5. Network Meta-Analysis Results for Inflammatory Biomarkers

Treatment rankings were summarized using the surface under the cumulative ranking curve (SUCRA) and were interpreted together with the magnitude and precision of the network estimates and the corresponding CINeMA confidence ratings. Higher SUCRA values indicate higher relative rankings but do not, by themselves, establish treatment superiority. The corresponding pairwise meta-analysis results are presented in Supplementary Table S2.

#### 3.5.1. IL-6

**Figure 3:**
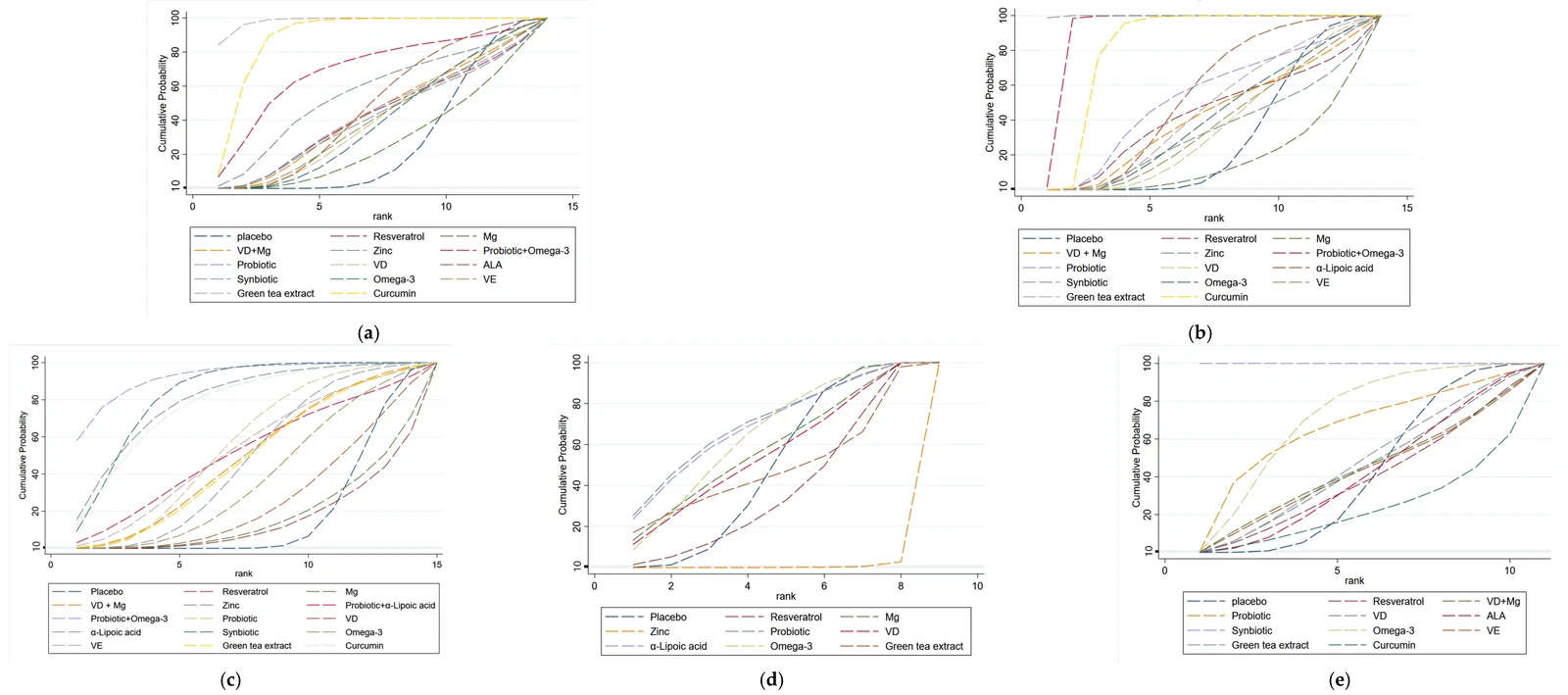
Cumulative ranking probability curves for nutritional supplementation strategies across the five inflammatory biomarkers. (**a**) Interleukin-6; (**b**) tumor necrosis factor-alpha; (**c**) *C*-reactive protein; (**d**) leptin; (**e**) adiponectin.

Curcumin (MD = −1.75, 95% CI [−2.74, −0.76]) and green tea extract (MD = −2.57, 95% CI [−3.99, −1.15]) were associated with significant reductions in IL-6 levels compared with placebo (Table 1). Based on the SUCRA rankings, green tea extract ranked highest (SUCRA = 97.5), followed by curcumin (SUCRA = 87.2), whereas magnesium ranked lowest (SUCRA = 22.5) (Figure 3, Table 4). The CINeMA assessment indicated high confidence in the network estimate for green tea extract and moderate confidence for curcumin (Supplementary Table S6). Therefore, although SUCRA summarizes the relative ranking of interventions, the favorable IL-6 findings for green tea extract and curcumin were also supported by comparatively stronger confidence in the corresponding network estimates.

#### 3.5.2. TNF-α

Synbiotics (MD = −12.25, 95% CI [−16.70, −7.80]), probiotics combined with omega-3 (MD = −6.19, 95% CI [−9.12, −3.26]), and curcumin (MD = −2.59, 95% CI [−3.83, −1.35]) were associated with significant reductions in TNF-α levels compared with placebo (Table 1). Based on the SUCRA rankings, Synbiotics ranked highest (SUCRA = 99.9), followed by probiotics combined with omega-3 (SUCRA = 92.3), and curcumin (SUCRA = 82.4) (Figure 3, Table 4). The CINeMA assessment indicated moderate confidence in the estimate for probiotics combined with omega-3, whereas the estimates for synbiotics and curcumin were rated as very low and low confidence, respectively (Table 5); confidence in the remaining comparisons ranged from low to very low, largely because of imprecision and, for several comparisons, concerns regarding indirectness. These findings therefore suggest potentially favorable effects of synbiotics, probiotics combined with omega-3, and curcumin on TNF-α, but the comparative rankings should be interpreted cautiously and in conjunction with the certainty of the underlying evidence.

#### 3.5.3. CRP

Zinc (MD = −1.18, 95% CI [−2.24, −0.11]), probiotics combined with omega-3 (MD = −1.74, 95% CI [−3.03, −0.46]), synbiotics (MD = −1.19, 95% CI [−1.85, −0.53]), and curcumin (MD = −1.11, 95% CI [−2.14, −0.07]) were associated with significant reductions in CRP levels compared with placebo (Table 2). Based on the SUCRA rankings, probiotics combined with omega-3 ranked highest (SUCRA = 92.35), followed by synbiotics (SUCRA = 82.84), zinc (SUCRA = 79.54), and curcumin (SUCRA = 77.3), whereas resveratrol ranked lowest (SUCRA = 15.24) (Figure 3, Table 4). The CINeMA assessment indicated low confidence in the network estimates for probiotics combined with omega-3, synbiotics, zinc, and curcumin (Table 5). The CINeMA assessment showed that confidence in the CRP network estimates was generally limited, with most comparisons rated as low or moderate and some as very low. Therefore, the observed ranking hierarchy should be considered exploratory rather than definitive.

#### 3.5.4. Leptin

No nutritional supplement was associated with a statistically significant reduction in leptin levels compared with placebo. Zinc supplementation was associated with a significant increase in leptin levels (MD = 19.20, 95% CI [6.64, 31.76]), indicating an unfavorable direction of effect (Table 3). According to the SUCRA rankings, probiotics (SUCRA = 70.1) and α-lipoic acid (SUCRA = 68.8) ranked highest for reducing leptin, whereas zinc ranked lowest (SUCRA = 0.5) (Figure 3, Table 4). However, the corresponding effect estimates for probiotics and α-lipoic acid were not statistically significant, and their higher SUCRA rankings should therefore not be interpreted as evidence of efficacy. Moreover, the CINeMA assessment rated the evidence for the zinc–placebo comparison as very low confidence, primarily because of concerns regarding indirectness and major imprecision (Supplementary Table S6).

#### 3.5.5. Adiponectin

Synbiotics (MD = 15.37, 95% CI [12.13, 18.61]) and probiotics (MD = 2.84, 95% CI [0.01, 5.69]) were associated with significant increases in adiponectin levels compared with placebo (Table 3). According to the SUCRA rankings, synbiotics ranked highest (SUCRA = 100), followed by probiotics (SUCRA = 84.4) (Figure 3, Table 4). However, the CINeMA assessment rated the confidence in both the synbiotic and probiotic network estimates as very low (Table 5). Therefore, despite the statistically significant estimates and high SUCRA rankings, these findings should be regarded as uncertain and require confirmation in larger, well-designed randomized controlled trials.

### 3.6. Meta-Regression Analysis

There exists a certain degree of heterogeneity in the effects of different nutritional interventions on inflammatory biomarkers. To further identify the sources of this heterogeneity and explore the relationships between potential factors and effect size, this study conducted meta-regression analyses for each inflammatory marker separately (Supplementary Table S5). The corresponding meta-regression plots are presented in Supplementary Figure S4. A random-effects model was used for the pooled analysis of each outcome measure, with intervention type, intervention duration, age, proportion of males, BMI, whether exercise intervention was combined, and whether dietary control was implemented used as individual covariates. Due to missing data in some studies, the relevant covariates were included in the corresponding analyses based on available data. Meta-regression employed the Restricted Maximum Likelihood (REML) method to establish univariate random-effects models.

The results showed that in the meta-regression analysis for IL-6, exercise intervention (β = −0.578, *p* = 0.003, R^2^ = 21.86%) and dietary control (β = −0.367, *p* = 0.010, R^2^ = 21.58%) both exerted significant negative moderating effects, suggesting that factors related to lifestyle interventions can, to some extent, explain the between-study variation in the effect size of IL-6.

In the analyses of CRP and adiponectin, exercise intervention was also identified as a key moderating variable. Specifically, its effect on the effect size of CRP was β = −0.484 (*p* = 0.022, R^2^ = 18.29%), whereas its effect on adiponectin was β = 1.816 (*p* = 0.014, R^2^ = 31.57%). These findings suggest that exercise intervention may exert differential modulatory effects on different outcome measures by influencing inflammatory levels and pathways related to lipid metabolism.

In the analysis of TNF-α and adiponectin, age was a significant moderator; its effect on TNF-α (β = 0.036, *p* = 0.008, R^2^ = 37.46%) and adiponectin (β = 0.076, *p* = 0.024, R^2^ = 33.75%), suggesting that different age distribution may influence the sources of variation in intervention effects. Additionally, the proportion of male participants emerged as a significant moderator in the meta-regression model for the effect size of adiponectin (β = 0.024, *p* = 0.002, R^2^ = 63.91%), indicating that differences in gender composition across studies may be a potential factor influencing the heterogeneity of the adiponectin response.

## 4. Discussion

This network meta-analysis suggests that nutritional supplementation may exert biomarker-specific effects on inflammation in adults with overweight or obesity. Several interventions were associated with favorable changes in CRP, IL-6, TNF-α, or adiponectin, whereas no supplement demonstrated a statistically significant reduction in leptin. Importantly, the confidence in the network estimates varied considerably across comparisons. The reductions in IL-6 associated with green tea extract and curcumin were supported by high- and moderate-confidence evidence, respectively, whereas some apparently favorable findings for other outcomes were supported by low or very-low-confidence evidence. In particular, the significant increases in adiponectin associated with synbiotics and probiotics were both rated as very low confidence. Therefore, SUCRA rankings should be interpreted together with the magnitude and precision of the effect estimates and the corresponding CINeMA confidence ratings, rather than as a definitive hierarchy of the most effective nutritional supplements.

### 4.1. The Effects of Different Types of Nutritional Supplements on Inflammatory Responses in Overweight and Obese Individuals

IL-6, primarily secreted by adipose tissue, muscle and the liver, functions as a pro-inflammatory cytokine and serves as a principal inducer of *C*-reactive protein synthesis in the liver. It plays a significant role in the development of chronic inflammation [31]. Clinical studies have demonstrated a positive correlation between plasma IL-6 concentrations and obesity; elevated IL-6 levels are associated with obesity-related insulin resistance and may also serve as predictive biomarkers for the onset of type 2 diabetes in various populations [32]. The present study revealed that, compared with the control group, green tea extract and curcumin significantly reduced IL-6 levels. Epigallocatechin gallate (EGCG), the major polyphenolic component in green tea, possesses potent anti-inflammatory potential and effectively reduces the levels of pro-inflammatory biomarkers, including IL-6. A secondary analysis of a randomised controlled trial involving overweight or obese individuals showed that supplementation with green tea extract (GTE) significantly modulates circulating inflammatory cytokines [33]. At the clinical level, a systematic review and meta-analysis focusing on patients with metabolic syndrome confirmed that dietary supplementation with curcumin significantly improves inflammatory markers in patients, including a reduction in serum IL-6 levels [34].

TNF-α is an important pro-inflammatory biomarker involved in the body’s inflammatory response and immune regulation. Elevated TNF-α levels are associated with reduced insulin sensitivity and increased fat accumulation [35]. Proliferating adipose tissue secretes large amounts of TNF-α [36]. Furthermore, TNF-α is also involved in the regulation of insulin resistance and dyslipidaemia, and is one of the risk factors for the development of type 2 diabetes [37]. Inhibition of TNF-α expression has been shown to improve obesity-induced hyperinsulinemia and alleviate oxidative stress. Therefore, reducing TNF-α levels in overweight and obese individuals is particularly important for their health management. In the present network, synbiotics ranked first for lowering TNF-α levels (SUCRA = 99.9); however, this ranking was based on a single trial of 30 participants and should be interpreted with caution. Numerous published studies and systematic reviews have demonstrated that synbiotic supplements can effectively reduce TNF-α levels in the body [38,39]. The mechanisms underlying their effect on tumour necrosis factor-α (TNF-α) levels primarily involve modulating the gut microbiota, enhancing intestinal barrier function, and directly or indirectly regulating immune cell function [40,41,42].

Furthermore, curcumin—a polyphenolic compound derived from turmeric (Curcuma longa L.), has demonstrated beneficial effects on TNF-α levels. Research indicates that curcumin significantly modulates IL-6 and TNF-α levels through multi-targeted, multi-pathway molecular mechanisms [43].

CRP is an acute-phase protein synthesized by the liver [44], and elevated CRP levels are associated with an increased risk of obesity-related diseases such as cardiovascular disease, diabetes and hepatic steatosis [45,46]. Consequently, reducing CRP levels is of great significance in mitigating the risk of obesity-related diseases. In a state of obesity, adipose tissue, acting as an endocrine organ, secretes various cytokines and hormones that promote elevated CRP levels; this is closely associated with chronic, low-grade inflammation in adipose tissue [47]. CRP can induce inflammatory responses and cause endothelial damage through its interaction with vascular endothelial cells. Additionally, it can also activate vascular smooth muscle cells, increasing cholesterol uptake and lipid deposition, thereby further promoting the development of atherosclerosis [48,49]. In this study, compared with the control group, some of the nutritional interventions significantly improved CRP levels; Analysis of the surface under the cumulative ranking curve indicated that the combination of probiotics and omega-3 fatty acids ranked first for improving CRP levels (SUCRA = 92.35), although this ranking rested on a single small trial (30 participants) whose direct estimate was not statistically significant; confirmation in larger trials is therefore required. The results of the network meta-analysis revealed that the combined use of probiotics and omega-3 fatty acids significantly outperformed the control group in reducing CRP levels; however, the network estimate indicated that the difference between the combined intervention and probiotics alone was not statistically significant (Table 2), and its apparent advantage over omega-3 alone was derived largely from a single small trial. Combined supplementation with probiotics and omega-3 fatty acids demonstrates significant synergistic potential in regulating CRP levels and alleviating chronic low-grade inflammation; the underlying mechanisms involve the strengthening of the intestinal barrier function, the remodelling of the gut microbiota, and the suppression of systemic inflammatory pathways [50,51,52]. Recent clinical studies have confirmed that the combined probiotic and omega-3 strategy demonstrates greater efficacy than single-supplement regimens in reducing key inflammatory markers, particularly in obese, elderly and metabolic syndrome patients [53,54].

Leptin and adiponectin are two principal hormones secreted by adipose tissue. Leptin plays a critical role in regulating energy balance, with elevated leptin levels contributing to the development of chronic inflammation. Conversely, adiponectin enhances insulin sensitivity and suppresses inflammatory responses. Both play a central role in energy homeostasis, glucose metabolism and the regulation of inflammation [55]. Furthermore, they are closely associated with obesity-related complications such as insulin resistance, impaired glucose tolerance and diabetes [56]. Excessive accumulation of visceral fat in obese patients leads to adipocyte hypertrophy and hypoxia, which in turn triggers chronic low-grade inflammation; this inflammatory state directly drives alterations in the adipokine secretion profile [57]. These alterations manifest specifically as abnormally elevated leptin levels and leptin resistance, as well as a significant decrease in adiponectin levels [58]. Previous studies have indicated that nutritional supplements can improve abnormal serum leptin and adiponectin levels in overweight and obese individuals; however, the conclusions of relevant studies remain controversial. In the present analysis, no nutritional supplement significantly reduced leptin levels; although probiotics ranked highest by SUCRA (70.1), the underlying effect estimate was not statistically significant, so this ranking cannot be interpreted as evidence of efficacy.

For adiponectin, synbiotics ranked first (SUCRA = 100) with a significant network estimate, but this result was driven by a single trial of 36 participants and requires confirmation. Furthermore, the network estimate for zinc suggested a significant increase in leptin whereas the corresponding direct comparison was non-significant; given the sparse direct evidence underlying this comparison and its very-low-confidence CINeMA rating, this estimate should be interpreted with caution. A meta-analysis of multiple randomised controlled trials showed that probiotic or synbiotic supplementation can significantly reduce inflammatory markers in individuals with prediabetes and type 2 diabetes, and regulate circulating levels of adiponectin and leptin [59]. A recent systematic review and meta-analysis reported that incorporating prebiotics, probiotics or synbiotics into weight-loss diets significantly improves leptin levels in overweight and obese adults [60]. Current evidence suggests that the potential mechanisms underlying the improvement of these two adipokines are jointly regulated by multiple factors, including genetics, diet, exercise, medical interventions, body fat distribution and the environment [61,62,63,64,65].

### 4.2. Meta-Regression Findings and Lifestyle Co-Interventions

This study used meta-regression analysis to further explore potential sources of between-study heterogeneity in the effects of nutritional supplementation on inflammatory biomarkers. The results indicated that the presence of exercise co-interventions was associated with variation in the estimated intervention effects for IL-6, CRP, and adiponectin, while dietary control was also associated with variation in the effect estimates for IL-6. Prior research has demonstrated that exercise may alleviate obesity-related metabolic dysfunction by reducing fat accumulation and low-grade chronic inflammation, thereby lowering baseline inflammatory levels at rest [66,67]. Dietary modification may likewise influence body weight, metabolic status, and systemic inflammation, suggesting that the background lifestyle context could contribute to differences in the observed responses to nutritional supplementation across studies.

Age was also associated with variation in the estimated effects on TNF-α and adiponectin, suggesting that participant age may contribute to between-study heterogeneity. Evidence indicates that, in the context of obesity and ageing, white adipose tissue undergoes fibrosis and hypoxia, promoting macrophage polarization towards the pro-inflammatory M1 phenotype and increased TNF-α production. At the same time, adiponectin bioavailability and the sensitivity of its downstream signalling pathways may be reduced, while adiponectin gene expression can be suppressed by pro-inflammatory biomarkers such as TNF-α [68]. The proportion of male participants was additionally associated with variation in adiponectin responses. Previous epidemiological and clinical studies have consistently reported sex-related differences in circulating adiponectin levels, with females generally exhibiting higher adiponectin concentrations [69,70]. These differences may be related to sex hormones, adipose tissue distribution, and metabolic status [71,72,73,74]. Therefore, differences in age and sex composition across studies may partly contribute to heterogeneity in the observed effects of nutritional supplementation on adiponectin and other inflammatory biomarkers.

These findings should, however, be interpreted as exploratory study-level associations rather than evidence of causal effect modification. Because the meta-regression analyses were based on aggregate study-level data, they cannot establish whether exercise or dietary interventions directly modify the effects of nutritional supplementation, nor can they determine whether the combined effects are additive, synergistic, or independent. Moreover, exercise and dietary co-interventions were required to be applied similarly across randomized groups within eligible trials; therefore, the present analysis did not directly compare supplementation alone with supplementation combined with lifestyle intervention. Future factorial or multi-arm randomized controlled trials comparing supplementation alone, lifestyle intervention alone, their combination, and an appropriate control would be better suited to formally evaluate potential supplement–lifestyle interactions.

### 4.3. Clinical and Practical Implications

These findings should be interpreted primarily as evidence regarding changes in inflammatory biomarkers rather than as recommendations for specific supplementation regimens. Considerable variation was observed across the included trials in supplement dose, formulation, and intervention duration. Green tea extract was administered at approximately 379–1500 mg/day for 4–52 weeks, whereas curcumin doses ranged from 80 to 1500 mg/day for 4–48 weeks. Synbiotic interventions also differed substantially in strain composition and dose, ranging from approximately 3 × 10^8^ CFU twice daily to 2 × 10^10^ CFU/day, with some formulations additionally containing prebiotic components such as inulin; intervention durations ranged from 8 to 52 weeks. Evidence for combined probiotic–omega-3 supplementation was more limited. One multi-arm trial administered VSL#3 at 1.125 × 10^11^ CFU/day together with 180 mg EPA and 120 mg DHA/day for 6 weeks, whereas another trial used one sachet/day of a probiotic–omega-3 PUFA formulation for 8 weeks. Given the substantial heterogeneity in dose, formulation, strain composition, and intervention duration, the present analysis does not allow conclusions regarding an optimal supplementation regimen. Therefore, the ranges reported above should be regarded as descriptions of the interventions evaluated in the included trials rather than as recommended clinical doses (Supplementary Table S1).

From a practical perspective, SUCRA rankings should be interpreted in conjunction with the magnitude and precision of the corresponding effect estimates, the amount of direct evidence, and the confidence in network estimates assessed using the Confidence in Network Meta-Analysis (CINeMA) framework. In particular, highly ranked interventions supported by a single small trial or by low- or very-low-confidence evidence should be considered preliminary and require confirmation in further adequately powered randomized controlled trials. Accordingly, nutritional supplements may be considered as potential adjunctive strategies for modulating selected inflammatory biomarkers, but the current evidence does not support their use as substitutes for established lifestyle-based approaches to the management of overweight and obesity.

### 4.4. Heterogeneity, Generalizability, and Evidence Gaps

Generalizability is constrained by substantial clinical heterogeneity across the included trials. Participants included individuals with type 2 diabetes, metabolic syndrome, polycystic ovary syndrome, non-alcoholic steatohepatitis, and other obesity-related or chronic comorbidities, in addition to otherwise overweight or obese populations. Baseline inflammatory status, BMI, comorbidity burden, supplement dose and formulation, intervention duration, and concomitant lifestyle programs are all plausible effect modifiers. These differences may challenge the plausibility of the transitivity assumption underlying valid indirect comparisons. In addition, the predominantly star-shaped network structure and the limited number of closed loops restricted formal evaluation of inconsistency between direct and indirect evidence. Accordingly, average network estimates should not be assumed to apply equally across all clinical subgroups.

Future randomized trials should prioritize several important evidence gaps. Replication is particularly needed for highly ranked comparisons supported by single or small trials, including synbiotics for TNF-α and adiponectin and probiotic–omega-3 combinations for selected inflammatory outcomes. Future trials should use standardized and fully described supplement formulations, prespecify dose and intervention duration, report adherence and adverse events, and provide consistent participant characteristics, including BMI, age, sex, comorbidities, and baseline inflammatory status. Multi-arm or factorial designs could directly compare combination supplements with their individual components and evaluate potential interactions with standardized lifestyle interventions such as diet and exercise. Longer follow-up and the inclusion of clinically meaningful outcomes, in addition to inflammatory biomarkers, would further improve the clinical relevance and generalizability of the evidence.

### 4.5. Strengths and Limitations of the Study

This study has several strengths. At the intervention level, it synthesised evidence on multiple commonly used nutritional supplements and compared their effects within a single analytical framework. At the outcome level, it simultaneously evaluated several inflammatory biomarkers, including CRP, IL-6, TNF-α, leptin, and adiponectin. Methodologically, network meta-analysis enabled the integration of both direct and indirect evidence, while meta-regression was used to explore potential sources of heterogeneity. In addition, the certainty of the network evidence was formally assessed using the Confidence in Network Meta-Analysis (CINeMA) framework, allowing the robustness of individual network estimates to be considered alongside the magnitude and ranking of treatment effects.

Several limitations should nevertheless be considered when interpreting the findings. First, substantial clinical and methodological heterogeneity existed across the included trials, including differences in supplement dose and formulation, intervention duration, baseline inflammatory status, underlying comorbidities, and participant characteristics. These differences limit the extent to which the findings can be used to derive specific supplementation regimens and may also affect the comparability of treatment effects across studies.

The validity of indirect comparisons depends on the transitivity assumption. Although clinically relevant potential effect modifiers were examined at the study level, the included populations were heterogeneous, and relevant characteristics were not consistently reported across all trials. In addition, most outcome-specific networks were predominantly star-shaped and contained relatively few closed loops. Consequently, formal statistical assessment of inconsistency was possible only for a limited subset of comparisons. The absence of statistically significant inconsistency should therefore not be interpreted as evidence of consistency across the entire network.

Intervention duration varied substantially across eligible trials, ranging from 2 to 52 weeks. Although intervention duration was explored as a potential moderator in the meta-regression analyses and was not statistically significant for most outcomes, the statistical power of these analyses was limited. Pooling very short-term and long-term interventions within the same treatment node may therefore have contributed to residual heterogeneity.

When the standard deviation of change scores was not directly reported, it was estimated using an assumed pre–post correlation coefficient of 0.50 because study-specific correlations were generally unavailable. Although this approach is commonly used when the actual correlation cannot be obtained, the assumed value may have influenced the precision of some effect estimates. Sensitivity analyses using alternative correlation coefficients were not performed; therefore, the robustness of the results to this assumption could not be formally evaluated.

Although the confidence in the network estimates was formally evaluated using CINeMA, several clinically relevant findings were supported by low- or very-low-confidence evidence. For example, the significant adiponectin effects associated with synbiotics and probiotics were both rated as very low confidence, and the apparent increase in leptin associated with zinc was also supported by very-low-confidence evidence. These ratings reflected concerns related to factors such as imprecision, heterogeneity, indirectness, and incoherence. Accordingly, statistically significant effects and high SUCRA rankings should not be interpreted as definitive evidence of clinical superiority when the corresponding confidence in the network estimate is limited.

Several potentially important effect modifiers could not be adequately examined because of incomplete reporting in the original studies. Changes in body weight were not consistently reported, detailed exercise modalities were often unavailable, and the distributions of age and sex varied across studies. Although exercise-related factors, age, and sex distribution were explored in meta-regression analyses where possible, residual confounding by these and other unmeasured study-level characteristics cannot be excluded.

Only English-language publications were included, which may have introduced language bias. In addition, for some outcomes, the evidence base was sparse and relied on small sample sizes, which may have reduced the precision and stability of both effect estimates and treatment rankings.

Finally, the outcomes evaluated in this review were circulating inflammatory biomarkers rather than clinical disease events. Therefore, the observed effects should be interpreted as biomarker-level findings and should not be extrapolated directly to the prevention or treatment of obesity-related clinical conditions. Overall, the findings are most appropriately interpreted at the level of adults with overweight or obesity as a broad population rather than being generalized to specific clinical subgroups.

## 5. Conclusions

This systematic review and network meta-analysis suggests that nutritional supplementation may influence circulating inflammatory biomarkers in adults with overweight or obesity in a biomarker-specific manner. Green tea extract and curcumin were associated with reductions in IL-6, with the corresponding network estimates supported by high- and moderate-confidence evidence, respectively. Several interventions also showed potentially favorable effects on TNF-α and CRP, although their relative rankings should be interpreted cautiously in light of the size and confidence of the underlying evidence base. No nutritional supplement significantly reduced leptin, and the observed increase in leptin associated with zinc was supported by very-low-confidence evidence. Synbiotics and probiotics were associated with increases in adiponectin, but CINeMA rated the confidence in both estimates as very low. Therefore, SUCRA rankings should not be interpreted as definitive treatment hierarchies or evidence of clinical superiority. The findings represent biomarker-level effects on surrogate outcomes and should not be extrapolated directly to the prevention or treatment of obesity-related clinical diseases. Further adequately powered randomized controlled trials using standardized supplementation protocols, longer intervention periods, and consistent reporting of participant and intervention characteristics are required to confirm these findings.

## Figures and Tables

**Table 1:** League table of the network meta-analysis results for IL-6 (interleukin-6) and TNF-α (tumor necrosis factor-alpha).

TNF-α													
**Resveratrol**	0.94 (−2.18, 4.06)	0.05 (−3.45, 3.55)	0.44 (−3.17, 4.06)	**−5.78 (−9.82, −1.74)**	−0.48 (−4.21, 3.25)	0.26 (−2.72, 3.25)	−0.29 (−3.26, 2.68)	**−11.84 (−17.09, −6.59)**	0.11 (−3.04, 3.26)	0.24 (−2.87, 3.35)	−0.04 (−3.11, 3.04)	−2.18 (−5.22, 0.87)	0.41 (−2.38, 3.20)
−0.51 (−2.25, 1.22)	**Mg**	−0.89 (−3.15, 1.37)	−0.49 (−3.19, 2.20)	**−6.72 (−9.97, −3.47)**	−1.42 (−4.28, 1.44)	−0.67 (−2.32, 0.97)	−1.22 (−2.97, 0.53)	**−12.78 (−17.45, −8.11)**	−0.83 (−2.87, 1.21)	−0.70 (−2.68, 1.28)	−0.98 (−2.90, 0.95)	**−3.11 (−4.99, −1.23)**	−0.53 (−1.94, 0.88)
−0.04 (−1.85, 1.76)	0.47 (−0.83, 1.77)	**VD + Mg**	0.39 (−2.74, 3.53)	**−5.83 (−9.45, −2.21)**	−0.53 (−3.80, 2.74)	0.22 (−1.96, 2.39)	−0.34 (−2.70,2.03)	**−11.89 (−16.82, −6.96)**	0.06 (−2.53, 2.65)	0.19 (−2.35, 2.72)	−0.09 (−2.58, 2.41)	−2.23 (−4.69, 0.24)	0.36 (−1.77, 2.49)
0.76 (−1.55, 3.06)	1.27 (−0.74, 3.29)	0.80 (−1.27, 2.87)	**Zinc**	**−6.22 (−9.94, −2.50)**	−0.92 (−4.31, 2.46)	−0.18 (−2.72, 2.37)	−0.73 (−3.04, 1.58)	**−12.28 (−17.29, −7.27)**	−0.33 (−3.00, 2.33)	−0.21 (−2.85, 2.44)	−0.48 (−3.12, 2.16)	**−2.62 (−5.23, −0.01)**	−0.03 (−2.33, 2.27)
1.66 (−1.23, 4.56)	2.18 (−0.50, 4.85)	1.71 (−1.01, 4.42)	0.91 (−2.16,3.98)	**Probiotic+ Omega-3**	**5.30 (1.46, 9.14)**	**6.04 (2.92, 9.17)**	**5.49 (2.39, 8.60)**	**−6.06 (−11.39, −0.73)**	**5.89 (2.61, 9.17)**	**6.02 (2.78, 9.25)**	**5.74 (2.54, 8.94)**	**3.60 (0.43, 6.78)**	**6.19 (3.26, 9.12)**
0.09 (−1.92, 2.11)	0.61 (−1.07, 2.29)	0.14 (−1.61, 1.88)	−0.66 (−2.92, 1.59)	−1.57 (−4.43, 1.29)	**Probiotic**	0.74 (−1.97, 3.46)	0.19 (−2.50, 2.88)	**−11.36 (−16.46, −6.26)**	0.59 (−2.30, 3.48)	0.72 (−2.13, 3.56)	0.44 (−2.36, 3.25)	−1.70 (−4.47, 1.08)	0.89 (−1.59, 3.37)
−0.08 (−1.82, 1.66)	0.44 (−0.79, 1.67)	−0.04 (−1.23, 1.16)	−0.84 (−2.85, 1.18)	−1.74 (−4.42, 0.93)	−0.17 (−1.85, 1.51)	**VD**	−0.55 (−2.05, 0.95)	**−12.10 (−16.69, −7.52)**	−0.15 (−1.99, 1.68)	−0.03 (−1.79, 1.73)	−0.30 (−2.00, 1.40)	**−2.44 (−4.09, −0.79)**	0.15 (−0.94, 1.23)
0.32 (−1.32, 1.96)	0.84 (−0.37, 2.04)	0.37 (−0.93, 1.66)	−0.43 (−2.21, 1.35)	−1.34 (−3.95, 1.27)	0.23 (−1.35, 1.81)	0.40 (−0.80, 1.61)	**α-Lipoic acid**	**−11.55 (−16.12, −6.98)**	0.40 (−1.19, 1.98)	0.52 (−1.07, 2.12)	0.25 (−1.42,1.91)	**−1.89 (−3.50, −0.28)**	0.70 (−0.34, 1.73)
−0.02 (−2.03, 2.00)	0.50 (−1.18, 2.17)	0.03 (−1.72, 1.77)	−0.77 (−3.03, 1.48)	−1.68 (−4.54, 1.18)	−0.11 (−2.07, 1.85)	0.06 (−1.61, 1.74)	−0.34 (−1.92, 1.24)	**Synbiotic**	**11.95 (7.26, 16.64)**	**12.08 (7.41, 16.74)**	**11.80 (7.16, 16.44)**	**9.66 (5.04, 14.29)**	**12.25 (7.80, 16.70)**
−0.04 (−1.71, 1.63)	0.47 (−0.77, 1.72)	0.00 (−1.33, 1.33)	−0.80 (−2.72, 1.12)	−1.70 (−4.33, 0.93)	−0.13 (−1.74, 1.48)	0.04 (−1.21, 1.28)	−0.36 (−1.32, 0.60)	−0.02 (−1.63, 1.58)	**Omega-3**	0.13 (−1.86, 2.11)	−0.15 (−2.12, 1.82)	**−2.29 (−4.21, −0.36)**	0.30 (−1.18, 1.77)
0.38 (−1.39, 2.16)	0.90 (−0.48, 2.27)	0.43 (−1.03, 1.88)	−0.37 (−2.39, 1.65)	−1.28 (−3.98, 1.42)	0.29 (−1.42, 2.01)	0.46 (−0.91, 1.84)	0.06 (−1.11, 1.23)	0.40 (−1.31, 2.11)	0.43 (−0.85, 1.70)	**VE**	−0.27 (−2.17, 1.63)	**−2.41 (−4.27, −0.55)**	0.17 (−1.21, 1.56)
**2.16 (0.13, 4.20)**	**2.68 (0.97, 4.39)**	**2.21 (0.44, 3.98)**	1.41 (−0.87, 3.69)	0.50 (−2.38, 3.38)	**2.07 (0.08, 4.06)**	**2.24 (0.54, 3.95)**	**1.84 (0.23, 3.45)**	**2.18 (0.20, 4.17)**	**2.21 (0.57, 3.84)**	**1.78 (0.04, 3.52)**	**Green tea extract**	**−2.14 (−3.94, −0.34)**	0.45 (−0.86, 1.75)
1.34 (−0.42, 3.11)	**1.86 (0.49, 3.22)**	1.39 (−0.06, 2.83)	0.59 (−1.45, 2.62)	−0.32 (−3.01, 2.37)	1.25 (−0.45, 2.95)	**1.42 (0.06, 2.79)**	1.02 (−0.22, 2.26)	1.36 (−0.34, 3.06)	**1.38 (0.11, 2.66)**	0.96 (−0.45, 2.37)	−0.82 (−2.55, 0.91)	**Curcumin**	**2.59 (1.35, 3.83)**
−0.41 (−1.87, 1.05)	0.11 (−0.83, 1.05)	−0.36 (−1.42, 0.69)	−1.16 (−2.94, 0.62)	−2.07 (−4.57, 0.43)	−0.50 (−1.89, 0.89)	−0.33 (−1.27, 0.61)	−0.73 (−1.48, 0.02)	−0.39 (−1.78, 1.00)	−0.37 (−1.18, 0.45)	−0.79 (−1.80, 0.21)	**−2.57 (−3.99, −1.15)**	**−1.75 (−2.74, −0.76)**	**Placebo**
**IL-6**													

Results are presented as mean differences with 95% confidence intervals. Statistically significant comparisons are shown in bold. For both IL-6 and TNF-α, a negative mean difference indicates a greater reduction in the biomarker level.

**Table 2:** League table of the network meta-analysis results for CRP (*C*-reactive protein).

CRP														
Resveratrol														
0.06 (−1.08, 1.20)	Mg													
0.63 (−0.50, 1.76)	0.57 (−0.45, 1.59)	VD + Mg												
**1.34 (0.01, 2.67)**	1.28 (−0.05, 2.61)	0.71 (−0.61, 2.03)	Zinc											
0.70 (−0.77, 2.16)	0.64 (−0.83, 2.10)	0.07 (−1.39, 1.52)	−0.64 (−2.24, 0.96)	Probiotic + ALA										
**1.91 (0.39, 3.42)**	**1.85 (0.33, 3.37)**	1.28 (−0.23, 2.79)	0.57 (−1.09, 2.23)	1.21 (−0.53, 2.95)	Probiotic + Omega-3									
0.74 (−0.27, 1.76)	0.68 (−0.33, 1.70)	0.11 (−0.89, 1.11)	−0.60 (−1.82, 0.62)	0.04 (−1.22, 1.31)	−1.17 (−2.49, 0.16)	Probiotic								
0.24 (−0.82, 1.31)	0.18 (−0.79, 1.16)	−0.39 (−1.27, 0.50)	−1.10 (−2.37, 0.18)	−0.45 (−1.87, 0.96)	**−1.66 (−3.13, −0.20)**	−0.50 (−1.43, 0.44)	VD							
0.60 (−0.37, 1.58)	0.54 (−0.43, 1.52)	−0.03 (−0.99, 0.94)	−0.74 (−1.85, 0.38)	−0.09 (−1.35, 1.16)	−1.30 (−2.68, 0.07)	−0.14 (−0.91, 0.64)	0.36 (−0.53, 1.25)	α-Lipoic acid						
**1.35 (0.31, 2.39)**	**1.29 (0.25, 2.33)**	0.72 (−0.31, 1.75)	0.01 (−1.24, 1.26)	0.65 (−0.73, 2.03)	−0.56 (−1.99, 0.88)	0.61 (−0.24, 1.46)	**1.11 (0.14, 2.07)**	0.75 (−0.11, 1.60)	Synbiotic					
0.44 (−0.58, 1.47)	0.38 (−0.64, 1.41)	−0.19 (−1.19, 0.82)	−0.90 (−2.11, 0.32)	−0.25 (−1.60, 1.10)	**−1.46 (−2.80, −0.13)**	−0.30 (−1.13, 0.53)	0.20 (−0.74, 1.14)	−0.16 (−0.92, 0.60)	−0.91 (−1.81, −0.00)	Omega-3				
0.71 (−0.51, 1.94)	0.65 (−0.57, 1.88)	0.08 (−1.13, 1.29)	−0.63 (−2.02, 0.76)	0.01 (−1.50, 1.53)	−1.20 (−2.78, 0.38)	−0.03 (−1.13, 1.07)	0.47 (−0.69, 1.63)	0.11 (−0.90, 1.11)	−0.64 (−1.78, 0.49)	0.27 (−0.84, 1.37)	VE			
0.62 (−0.48, 1.72)	0.56 (−0.54, 1.66)	−0.01 (−1.10, 1.08)	−0.72 (−2.02, 0.58)	−0.07 (−1.51, 1.36)	−1.28 (−2.77, 0.20)	−0.12 (−1.09, 0.85)	0.38 (−0.65, 1.41)	0.02 (−0.91, 0.95)	−0.73 (−1.73, 0.27)	0.18 (−0.80, 1.16)	−0.09 (−1.27, 1.10)	Green tea extract		
1.27 (−0.04, 2.58)	1.21 (−0.10, 2.52)	0.64 (−0.66, 1.94)	−0.07 (−1.55, 1.41)	0.57 (−1.03, 2.18)	−0.64 (−2.29, 1.01)	0.53 (−0.68, 1.73)	1.03 (−0.22, 2.28)	0.67 (−0.51, 1.84)	−0.08 (−1.31, 1.15)	0.83 (−0.38, 2.04)	0.56 (−0.83, 1.95)	0.65 (−0.63, 1.93)	Curcumin	
0.16 (−0.64, 0.97)	0.11 (−0.70, 0.91)	−0.47 (−1.25, 0.32)	**−1.18 (−2.24, −0.11**)	−0.53 (−1.76, 0.69)	**−1.74 (−3.03, −0.46)**	−0.58 (−1.19, 0.04)	−0.08 (−0.78, 0.62)	−0.44 (−0.99, 0.11)	**−1.19 (−1.85, −0.53)**	−0.28 (−0.91, 0.35)	−0.55 (−1.47, 0.38)	−0.46 (−1.21, 0.29)	**−1.11 (−2.14, −0.07)**	Placebo

Results are presented as mean differences with 95% confidence intervals. Statistically significant comparisons are shown in bold. For CRP (*C*-reactive protein), a negative mean difference indicates a greater reduction in the biomarker level.

**Table 3:** League table of the network meta-analysis results for Adiponectin and leptin.

Adiponectin												
Resveratrol	————	0.46 (−2.83, 3.74)	————	3.29 (−0.19, 6.77)	0.55 (−2.26, 3.37)	0.83 (−1.71, 3.38)	**15.82 (12.01, 19.63)**	1.08 (−1.37, 3.53)	0.58 (−2.69, 3.86)	0.82 (−1.76, 3.41)	−0.12 (−3.55, 3.32)	0.45 (−1.55, 2.45)
2.24 (−6.22, 10.71)	Mg	————	————	————	————	————	————	————	————	————	————	————
————	————	VD + Mg	————	2.83 (−1.02, 6.69)	0.10 (−2.50, 2.70)	0.38 (−2.66, 3.42)	**15.36 (11.21, 19.51)**	0.62 (−2.33, 3.58)	0.13 (−3.54, 3.79)	0.37 (−2.70, 3.44)	−0.57 (−4.39, 3.24)	−0.01 (−2.61, 2.59)
**−17.84 (−30.23, −5.45)**	**−20.08 (−34.41, −5.76)**	————	Zinc	————	————	————	————	————	————	————	————	————
3.81 (−4.68, 12.30)	1.57 (−8.20, 11.34)	————	**21.65 (7.31, 36.00)**	Probiotic	−2.74 (−6.20, 0.73)	−2.46 (−5.71, 0.80)	**12.53 (8.22, 16.84)**	−2.21 (−5.39, 0.97)	−2.71 (−6.55, 1.14)	−2.47 (−5.75, 0.82)	−3.41 (−7.39, 0.58)	**−2.84 (−5.69, −0.01)**
1.90 (−6.42, 10.22)	−0.34 (−9.97, 9.29)	————	**19.74 (5.50, 33.99)**	−1.91 (−11.56, 7.74)	VD	0.28 (−2.25, 2.81)	**15.27 (11.48, 19.06)**	0.53 (−1.90, 2.96)	0.03 (−3.22, 3.28)	0.27 (−2.29, 2.83)	−0.67 (−4.09, 2.75)	−0.10 (−2.08, 1.87)
3.66 (−4.78, 12.11)	1.42 (−8.32, 11.16)	————	**21.50 (7.19, 35.82)**	−0.15 (−9.91, 9.61)	1.76 (−7.85, 11.37)	α-Lipoic acid	**14.99 (11.39, 18.58)**	0.25 (−1.67, 2.16)	−0.25 (−2.84, 2.34)	−0.01 (−2.28, 2.26)	−0.95 (−4.12, 2.22)	−0.38 (−1.96, 1.19)
————	————	————	————	————	————	————	Synbiotic	**−14.74 (−18.27, −11.21)**	**−15.24 (−19.38, −11.09)**	**−15.00 (−18.62, −11.37)**	**−15.94 (−20.21, −11.67)**	**−15.37 (−18.61, −12.13)**
1.77 (−4.37, 7.91)	−0.47 (−8.30, 7.36)	————	**19.61 (6.53, 32.70)**	−2.04 (−9.90, 5.81)	−0.13 (−7.80, 7.54)	−1.89 (−9.70, 5.92)	————	Omega−3	−0.50 (−3.37, 2.38)	−0.26 (−2.41, 1.90)	−1.20 (−4.33, 1.93)	−0.63 (−2.04, 0.78)
————	————	————	————	————	————	————	————	————	VE	0.24 (−2.82,3.30)	−0.70 (−4.49,3.09)	−0.13 (−2.72,2.45)
0.56 (−12.66, 13.78)	−1.68 (−15.76, 12.40)	————	**18.40 (0.84, 35.97)**	−3.25 (−17.35, 10.85)	−1.34 (−15.34, 12.66)	−3.10 (−17.17, 10.97)	————	−1.21 (−14.03, 11.62)	————	Green tea extract	−0.94 (−4.17, 2.28)	−0.37 (−2.01, 1.26)
————	————	————	————	————	————	————	————	————	————	————	Curcumin	0.57 (−2.22, 3.35)
1.36 (−3.55, 6.27)	−0.88 (−7.78, 6.02)	————	**19.20 (6.64, 31.76)**	−2.45 (−9.38, 4.48)	−0.54 (−7.26, 6.18)	−2.30 (−9.17, 4.57)	————	−0.41 (−4.11, 3.30)	————	0.80 (−11.48, 13.08)	————	Placebo
leptin												

Results are presented as mean differences with 95% confidence intervals. Statistically significant comparisons are shown in bold. For both Adiponectin and leptin, a negative MD indicates a greater reduction in leptin, whereas a positive MD indicates a greater increase in adiponectin.

**Table 4:** Surface under the cumulative ranking curve values and relative rankings of nutritional supplementation strategies for each inflammatory biomarker.

Treatment	IL-6		TNF-α		CRP		Leptin		Adiponectin	
	SUCRA (%)	RANK	SUCRA (%)	RANK	SUCRA (%)	RANK	SUCRA (%)	RANK	SUCRA (%)	RANK
Placebo	22.3	14	29.2	13	17.86	13	48.2	6	36.2	9
Resveratrol	40.6	8	42.5	7	15.24	15	37.2	8	27.2	11
Mg	22.5	13	16.7	14	17.59	14	57.7	4	NA	NA
VD + Mg	38.9	10	41.4	8	48.79	8	NA	NA	39.1	8
Zinc	58	4	32.5	12	79.54	3	0.5	9	NA	NA
Probiotic + ALA	NA	NA	NA	NA	51.16	7	NA	NA	NA	NA
Probiotic + Omega-3	74.1	3	92.3	2	92.35	1	NA	NA	NA	NA
Probiotics	42.1	7	52.2	5	55.47	5	70.1	1	84.4	2
VD	37.9	11	35.7	11	25.86	12	55.3	5	40.4	7
α-Lipoic acid	48.4	6	54	4	47.39	10	68.8	2	48.6	4
Synbiotics	39.5	9	99.9	1	82.84	2	NA	NA	100	1
Omega-3	37.4	12	40	9	37.46	11	64.1	3	56.5	3
VE	53.7	5	36.4	10	52.96	6	NA	NA	41.8	6
Green tea extract	97.5	1	44.9	6	47.61	9	48.1	7	48.2	5
Curcumin	87.2	2	82.4	3	77.3	4	NA	NA	27.7	10

**Table 5:** Summary of selected higher-ranked interventions, network effect estimates, SUCRA values, and CINeMA confidence.

Biomarker	Selected Higher-Ranked Findings	SUCRA (%)	CINeMA/Evidence Note
IL-6	Green tea extract: −2.57 (−3.99 to −1.15)	97.5	High
	Curcumin: −1.75 (−2.74 to −0.76)	87.2	Moderate
TNF-α	Synbiotics: −12.25 (−16.70 to −7.80)	99.9	Very low
	Probiotics + omega-3: −6.19 (−9.12 to −3.26)	92.3	Moderate
	Curcumin −2.59 (−3.83 to −1.35)	82.4	low
CRP	Probiotics + omega-3: −1.74 (−3.03 to −0.46)	92.35	low
	Synbiotics: −1.19 (−1.85 to −0.53)	82.84	low
	Zinc: −1.18 (−2.24 to −0.11)	79.54	low
	Curcumin: −1.11 (−2.14 to −0.07)	77.3	low
Leptin	No nutritional supplement showed a statistically significant reduction	—	Higher SUCRA ranks were not supported by significant reductions
Adiponectin	Synbiotics: +15.37 (12.13 to 18.61)	100	Very low
	Probiotics: +2.84 (0.01 to 5.69)	84.4	Very low

A negative MD indicates a reduction in IL-6, TNF-α, CRP, or leptin; a positive MD indicates an increase in adiponectin. SUCRA reflects relative ranking and should be interpreted together with effect size, precision, amount of direct evidence, and CINeMA confidence.

## Data Availability

No new data were created or analyzed in this study. Data sharing is not applicable to this article.

## Supplementary Materials

The following supporting information can be downloaded at https://www.mdpi.com/article/10.3390/nu18172936/s1, Supplementary File S1. PRISMA NMA checklist; Supplementary File S2. Search strategy of PubMed, Embase, Cochrane, Web of Science, and EBSCO; Supplementary Table S1. Characteristics of included studies; Supplementary File S3. List of included studies; Supplementary Table S2. Pairwise meta-analysis; Supplementary Figure S1. Network plot of different nutrition supplements for overweight and obese treatment; Supplementary Table S3. Inconsistency of IL-6, TNF-α, CRP, leptin, adiponectin, tested by loop-specific heterogeneity estimates, inconsistency model and node splitting analysis; Supplementary Table S4. Risk of bias table; Supplementary Figure S2. Risk of bias graph; Supplementary Figure S3. The funnel plot graphics of IL-6, TNF-α, CRP, leptin, and adiponectin in NMA; Supplementary Table S5. Results of Meta-regression; Supplementary Figure S4. Results of Meta-regression graph; Supplementary Table S6. CINeMA assessment of the confidence.

## Abbreviations

The following abbreviations are used in this manuscript:

RCT	Randomized Controlled Trial
NMA	Network Meta-Analysis
CRP	*C*-Reactive Protein
IL-6	Interleukin-6
TNF-α	Tumor Necrosis Factor-alpha
SUCRA	Surface Under the Cumulative Ranking Curve
MD	Mean Difference
CI	Confidence Interval
BMI	Body Mass Index
PRISMA	Preferred Reporting Items for Systematic Reviews and Meta-Analyses
ROB	Risk of Bias
REML	Restricted Maximum Likelihood
ALA	α-Lipoic Acid
VD	Vitamin D
VE	Vitamin E
EGCG	Epigallocatechin-3-Gallate
