# Supplementary material for: Effects of Nutritional Supplements on Inflammatory Biomarkers in Overweight and Obese Adults: A Systematic Review and Network Meta-Analysis of Randomized Controlled Trials
Source: Nutrients. 2026 Sep 7;18(17):2936. doi: 10.3390/nu18172936 (PMC13567556; doi:10.3390/nu18172936)
Supplement: Supplementary file 1 [file nutrients-18-02936-s001.zip › nutrients-4514635-supplementary.pdf]

SUPPLEMENTARY MATERIAL TO THE MANUSCRIPT

Effects of Nutritional Supplements on Inflammatory Biomarkers in Overweight and Obese Adults: A Systematic Review and Network Meta-Analysis of Randomized Controlled Trials

Zhiyue Chen, Ailing Liu, Shuran Bian, Zhize Zhou, Dong Zhang

Supplemental materials	Page
**Supplementary File S1.** PRISMA NMA checklist.	1-4
**Supplementary File S2.** Search strategy of PubMed, Embase, Cochrane, Web of Science, and EBSCO.	5-6
**Supplementary Table S1.** Characteristics of included studies.	7-13
**Supplementary File S3.** List of included studies.	14-18
**Supplementary Table S2.** Pairwise meta-analysis	19-21
**Supplementary Figure S1.** Network plot of different nutrition supplements for overweight and obese treatment	22-23
**Supplementary Table S3.** Inconsistency of IL-6, TNF-α, CRP, leptin, adiponectin, tested by loop-specific heterogeneity estimates, inconsistency model and node splitting analysis	24-26
**Supplementary Table S4.** Risk of bias table.	27-28
**Supplementary Figure S2.** Risk of bias graph.	29-30
**Supplementary Figure S3.** The funnel plot graphics of IL-6, TNF-α,CRP, leptin, and adiponectin in NMA.	31-33
**Supplementary Table S5.** Results of Meta-regression.	34
**Supplementary Figure S4.** Results of Meta-regression graph.	35-38
**Supplementary Table S6.** CINeMA assessment of the confidence.	39-43

Supplementary File S1
PRISMA 2020 Checklist

	Item #	Checklist item	Location where item is reported
**TITLE**			
Title	1	Identify the report as a systematic review.	1
**ABSTRACT**			
Abstract	2	See the PRISMA 2020 for Abstracts checklist.	2
**INTRODUCTION**			
Rationale	3	Describe the rationale for the review in the context of existing knowledge.	2-4
Objectives	4	Provide an explicit statement of the objective(s) or question(s) the review addresses.	4
**METHODS**			
Eligibility criteria	5	Specify the inclusion and exclusion criteria for the review and how studies were grouped for the syntheses.	4-5
Information sources	6	Specify all databases, registers, websites, organisations, reference lists and other sources searched or consulted to identify studies. Specify the date when each source was last searched or consulted.	4
Search strategy	7	Present the full search strategies for all databases, registers and websites, including any filters and limits used.	4，Supplementary file 2.
Selection process	8	Specify the methods used to decide whether a study met the inclusion criteria of the review, including how many reviewers screened each record and each report retrieved, whether they worked independently, and if applicable, details of automation tools used in the process.	4
Data collection process	9	Specify the methods used to collect data from reports, including how many reviewers collected data from each report, whether they worked independently, any processes for obtaining or confirming data from study investigators, and if applicable, details of automation tools used in the process.	5
Data items	10a	List and define all outcomes for which data were sought. Specify whether all results that were compatible with each outcome domain in each study were sought (e.g. for all measures, time points, analyses), and if not, the methods used to decide which results to collect.	4-5
	10b	List and define all other variables for which data were sought (e.g. participant and intervention characteristics, funding sources). Describe any assumptions made about any missing or unclear information.	5

	Item #	Checklist item	Location where item is reported
Study risk of bias assessment	11	Specify the methods used to assess risk of bias in the included studies, including details of the tool(s) used, how many reviewers assessed each study and whether they worked independently, and if applicable, details of automation tools used in the process.	5-6
Effect measures	12	Specify for each outcome the effect measure(s) (e.g. risk ratio, mean difference) used in the synthesis or presentation of results.	6
Synthesis methods	13a	Describe the processes used to decide which studies were eligible for each synthesis (e.g. tabulating the study intervention characteristics and comparing against the planned groups for each synthesis (item #5)).	4-5
	13b	Describe any methods required to prepare the data for presentation or synthesis, such as handling of missing summary statistics, or data conversions.	5-6
	13c	Describe any methods used to tabulate or visually display results of individual studies and syntheses.	6-7
	13d	Describe any methods used to synthesize results and provide a rationale for the choice(s). If meta-analysis was performed, describe the model(s), method(s) to identify the presence and extent of statistical heterogeneity, and software package(s) used.	6
	13e	Describe any methods used to explore possible causes of heterogeneity among study results (e.g. subgroup analysis, meta-regression).	6
	13f	Describe any sensitivity analyses conducted to assess robustness of the synthesized results.	-
Reporting bias assessment	14	Describe any methods used to assess risk of bias due to missing results in a synthesis (arising from reporting biases).	6，Figure 3.
Certainty assessment	15	Describe any methods used to assess certainty (or confidence) in the body of evidence for an outcome.	6-7
**RESULTS**			
Study selection	16a	Describe the results of the search and selection process, from the number of records identified in the search to the number of studies included in the review, ideally using a flow diagram.	6-7<br>Figure 1.
	16b	Cite studies that might appear to meet the inclusion criteria, but which were excluded, and explain why they were excluded.	6-7
Study characteristics	17	Cite each included study and present its characteristics.	7-8, Supplementary Table 1.
Risk of bias in studies	18	Present assessments of risk of bias for each included study.	8, Supplementary Figure 2 and

	Item #	Checklist item	Location where item is reported
			Supplementary Table 4.
Results of individual studies	19	For all outcomes, present, for each study: (a) summary statistics for each group (where appropriate) and (b) an effect estimate and its precision (e.g. confidence/credible interval), ideally using structured tables or plots.	11-15，Supplementary Table 2.
Results of syntheses	20a	For each synthesis, briefly summarise the characteristics and risk of bias among contributing studies.	7-8
	20b	Present results of all statistical syntheses conducted. If meta-analysis was done, present for each the summary estimate and its precision (e.g. confidence/credible interval) and measures of statistical heterogeneity. If comparing groups, describe the direction of the effect.	8-16, Tables 1-4 and Figures2-3;Supplementary Tables 2-3.
	20c	Present results of all investigations of possible causes of heterogeneity among study results.	18, Supplementary Figure S4 and Table S5.
	20d	Present results of all sensitivity analyses conducted to assess the robustness of the synthesized results.	-
Reporting biases	21	Present assessments of risk of bias due to missing results (arising from reporting biases) for each synthesis assessed.	9, Supplementary Figure 3.
Certainty of evidence	22	Present assessments of certainty (or confidence) in the body of evidence for each outcome assessed.	Supplementary Table 6.
**DISCUSSION**			
Discussion	23a	Provide a general interpretation of the results in the context of other evidence.	18-21
	23b	Discuss any limitations of the evidence included in the review.	21
	23c	Discuss any limitations of the review processes used.	21
	23d	Discuss implications of the results for practice, policy, and future research.	21-22
**OTHER INFORMATION**			

	Item #	Checklist item	Location where item is reported
Registration and protocol	24a	Provide registration information for the review, including register name and registration number, or state that the review was not registered.	4, PROSPERO CRD420261348823.
	24b	Indicate where the review protocol can be accessed, or state that a protocol was not prepared.	-
	24c	Describe and explain any amendments to information provided at registration or in the protocol.	-
Support	25	Describe sources of financial or non-financial support for the review, and the role of the funders or sponsors in the review.	22
Competing interests	26	Declare any competing interests of review authors.	22
Availability of data, code and other materials	27	Report which of the following are publicly available and where they can be found: template data collection forms; data extracted from included studies; data used for all analyses; analytic code; any other materials used in the review.	22

Supplementary File S2

Search strategy of PubMed, Embase, Cochrane, Web of Science, and EBSCO

Database	Search Terms	Number of records
PubMed	("Overweight"[Mesh] OR "Obesity"[Mesh] OR "Body Mass Index"[Mesh] OR "Adiposity"[Mesh] OR overweight*[tiab] OR obese[tiab] OR obesity[tiab] OR "body mass index"[tiab] OR BMI[tiab] OR "body weight"[tiab] OR "weight loss"[tiab] OR "weight gain"[tiab] OR adipos*[tiab] OR "fat mass"[tiab] OR "waist circumference"[tiab]) AND ("Magnesium"[Mesh] OR magnesium[tiab] OR Mg[tiab] OR Resveratrol[Mesh] OR "Vitamin D"[Mesh] OR "vitamin d"[tiab] OR cholecalciferol[tiab] OR ergocalciferol[tiab] OR calcitriol[tiab] OR "Zinc"[Mesh] OR zinc[tiab] OR "Probiotics"[Mesh] OR probiotic*[tiab] OR lactobacillus[tiab] OR bifidobacterium[tiab] OR "Thioctic Acid"[Mesh] OR "alpha-lipoic acid"[tiab] OR "lipoic acid"[tiab] OR "thioctic acid"[tiab] OR ALA[tiab] OR "Fatty Acids, Omega-3"[Mesh] OR "omega-3"[tiab] OR "n-3 fatty acids"[tiab] OR "eicosapentaenoic acid"[tiab] OR EPA[tiab] OR "docosahexaenoic acid"[tiab] OR DHA[tiab] OR "fish oil"[tiab] OR "Synbiotics"[Mesh] OR synbiotic*[tiab] OR "Vitamin E"[Mesh] OR "vitamin e"[tiab] OR tocopherol[tiab] OR tocotrienol[tiab] OR "Camellia sinensis"[Mesh] OR "green tea"[tiab] OR "tea extract"[tiab] OR epigallocatechin gallate[tiab] OR EGCG[tiab] OR "Curcumin"[Mesh] OR curcumin[tiab] OR turmeric[tiab]) AND ("Inflammation"[Mesh] OR inflamm*[tiab] OR "inflammatory markers"[tiab] OR "inflammatory mediators"[tiab]) AND (randomized controlled trial[pt] OR controlled clinical trial[pt] OR randomized[tiab] OR placebo[tiab] OR randomly[tiab] OR trial[tiab] OR groups[tiab])	649
Embase	('obesity'/exp OR 'overweight'/exp OR obes*:ti,ab,kw OR overweight*:ti,ab,kw) AND ('interleukin 6'/exp OR 'tumor necrosis factor alpha'/exp OR 'c reactive protein'/exp OR 'leptin'/exp OR 'adiponectin'/exp OR 'il 6':ti,ab,kw OR 'tnf alpha':ti,ab,kw OR 'tnf α':ti,ab,kw OR crp:ti,ab,kw OR leptin:ti,ab,kw OR adiponectin:ti,ab,kw OR 'inflammation'/exp OR 'innate inflammatory response':ti,ab,kw OR 'inflammatory response, innate':ti,ab,kw OR inflammatory) AND marker*:ti,ab,kw AND ('magnesium'/exp OR magnesium:ti,ab,kw OR mg:ti,ab,kw OR 'vitamin d'/exp OR 'vitamin d':ti,ab,kw OR vd:ti,ab,kw OR 'zinc'/exp OR zinc:ti,ab,kw OR resveratrol:ti,ab,kw OR 'probiotic agent'/exp OR probiotic*:ti,ab,kw OR 'synbiotic agent'/exp OR synbiotic*:ti,ab,kw OR 'alpha lipoic acid'/exp OR 'alpha lipoic acid':ti,ab,kw OR ala:ti,ab,kw OR 'omega 3 fatty acid'/exp OR 'omega 3':ti,ab,kw OR pufa:ti,ab,kw OR 'vitamin e'/exp OR 'vitamin e':ti,ab,kw OR ve:ti,ab,kw OR 'green tea extract'/exp OR 'green tea extract':ti,ab,kw OR 'curcumin'/exp OR curcumin:ti,ab,kw) AND ('randomized controlled trial'/exp OR 'clinical trial'/exp OR random*:ti,ab,kw OR placebo:ti,ab,kw) AND (placebo:ti,ab,kw OR control*:ti,ab,kw OR compar*:ti,ab,kw) AND ('adult'/exp OR adult*:ti,ab,kw)	1582
Cochrane	((overweight):ti,ab,kw OR (obesity):ti,ab,kw OR (obese):ti,ab,kw) AND ((magnesium):ti,ab,kw OR (Mg):ti,ab,kw OR (vitamin D):ti,ab,kw OR (cholecalciferol):ti,ab,kw OR (ergocalciferol):ti,ab,kw OR (zinc):ti,ab,kw OR (Zn):ti,ab,kw OR (probiotic):ti,ab,kw OR (probiotics):ti,ab,kw OR (lactobacillus):ti,ab,kw OR (bifidobacterium):ti,ab,kw OR (alpha lipoic	1188

	acid):ti,ab,kw OR (Thioctic Acid):ti,ab,kw OR (ALA):ti,ab,kw OR (omega-3):ti,ab,kw OR (omega-3 fatty acid):ti,ab,kw OR (n-3 PUFA):ti,ab,kw OR (eicosapentaenoic acid):ti,ab,kw OR (EPA):ti,ab,kw OR (docosahexaenoic acid):ti,ab,kw OR (DHA):ti,ab,kw OR (fish oil):ti,ab,kw OR (synbiotic):ti,ab,kw OR (synbiotics):ti,ab,kw OR (vitamin E):ti,ab,kw OR (tocopherol):ti,ab,kw OR (tocotrienol):ti,ab,kw OR (green tea):ti,ab,kw OR (tea extract):ti,ab,kw OR (EGCG):ti,ab,kw OR (epigallocatechin gallate):ti,ab,kw OR (curcumin):ti,ab,kw OR (turmeric):ti,ab,kw) AND ((inflammation):ti,ab,kw OR (inflammatory marker):ti,ab,kw OR (inflammatory mediator):ti,ab,kw OR (cytokine):ti,ab,kw OR (adipokine):ti,ab,kw OR (interleukin-6):ti,ab,kw OR (IL-6):ti,ab,kw OR (tumor necrosis factor-alpha):ti,ab,kw OR (TNF-alpha):ti,ab,kw OR (TNF-α):ti,ab,kw OR (C-reactive protein):ti,ab,kw OR (CRP):ti,ab,kw OR (leptin):ti,ab,kw OR (adiponectin):ti,ab,kw) AND ((randomized controlled trial):ti,ab,kw OR (randomized):ti,ab,kw OR (placebo):ti,ab,kw OR (randomly):ti,ab,kw) AND ((supplementation):ti,ab,kw OR (intake):ti,ab,kw)	
Web of Science	TS=((overweight OR obesity OR adiposity OR obese OR "weight loss" OR "weight gain" OR adipos* OR "fat mass" OR "waist circumference")AND (magnesium OR Mg OR "vitamin D" OR cholecalciferol OR "25-hydroxyvitamin D" OR "25(OH)D" OR zinc OR Zn OR probiotic* OR lactobacillus OR bifidobacterium OR saccharomyces OR "streptococcus thermophilus" OR "alpha-lipoic acid" OR "lipoic acid" OR "thioctic acid" OR ALA OR "omega-3" OR "n-3 fatty acid*" OR "eicosapentaenoic acid" OR EPA OR "docosahexaenoic acid" OR "resveratrol" OR DHA OR "fish oil" OR synbiotic* OR symbiotic* OR "vitamin E" OR tocopherol* OR tocotrienol* OR "green tea" OR "tea extract" OR "camellia sinensis" OR "epigallocatechin gallate" OR EGCG OR curcumin OR turmeric OR curcuma)AND (inflamm* OR "inflammatory marker*" OR "inflammatory mediator*" OR "interleukin-6" OR "IL-6" OR "tumor necrosis factor-alpha" OR "TNF-alpha" OR "TNF-α" OR "C-reactive protein" OR CRP OR leptin OR adiponectin) AND (randomized OR "randomized controlled trial" OR "controlled clinical trial" OR placebo OR randomly OR trial OR groups OR "double-blind" OR "single-blind" OR "clinical trial"))AND WC=("Nutrition & Dietetics")	1858
EBSCO	(MH "Obesity" OR MH "Overweight" OR TI (overweight OR obesity OR obese) OR AB (overweight OR obesity OR obese)) AND (MH "Magnesium" OR MH "Vitamin D" OR MH "Zinc" OR MH "Probiotics" OR MH "Fatty Acids, Omega-3" OR TI (magnesium OR "vitamin D" OR zinc OR probiotic* OR omega-3 OR EPA OR DHA OR "fish oil" OR synbiotic* OR curcumin) OR AB (magnesium OR "vitamin D" OR zinc OR probiotic* OR omega-3)) AND (MH "Inflammation" OR MH "Interleukin-6" OR MH "Tumor Necrosis Factor-alpha" OR MH "C-Reactive Protein" OR MH "Leptin" OR MH "Adiponectin" OR TI (cytokine* OR "IL-6" OR "TNF-alpha" OR CRP OR leptin OR adiponectin) OR AB ("IL-6" OR "TNF-alpha" OR CRP OR leptin OR adiponectin)) AND (MH "Randomized Controlled Trial" OR TI (randomized OR placebo) OR AB (randomized OR placebo))	302

Supplementary Table S1

Characteristics of included studies.

Author	Year	Country	Sample		Age Mean(SD) /Range	Male %	BMI	Interventions	Exercise	Diet control	Duration (week)	Outcome
			T	C								
Walker et al. [1]	2018	America	14	14	47 ± 8<br>48 ± 9	100	35.0 ± 3.0<br>33.8 ± 3.3	Placebo vs. Resveratrol (2000 mg, b.i.d.)	yes	yes	4	CRP
Arzola et al. [2]	2016	Mexico	15	24	33.7 ± 11.9<br>38.8 ± 9.6	15.4	35.6 ± 2.7<br>34.7 ± 2.9	Placebo vs. Resveratrol (300 mg, q.d.)	no	yes	24	Leptin, Adiponectin
Kantartzis et al. [3]	2018	Germany	52	53	18-70	100	33-35	Placebo vs. Resveratrol (150 mg, q.d.)	no	no	12	IL-6,CRP, Adiponectin
Poulsen et al. [4]	2012	Denmark	12	12	44.7 ± 12.1<br>31.9 ± 10	100	32.5 ± 0.6<br>35.9 ± 1.2	Placebo vs. Resveratrol (500 mg, q.d.)	no	no	4	IL-6,TNF-α,CRP, Leptin
Seyyedebrahimi et al. [5]	2018	Iran	23	23	54.96 ± 6.37<br>58.72 ± 6.06	52	29.0 ± 3.4<br>28.9 ± 5.3	Placebo vs. Resveratrol (400 mg ,b.i.d.)	no	yes	8	CRP
Joris et al. [6]	2017	Netherlands	26	25	62 ± 6<br>62 ± 6	57.7	29.6 ± 2.8<br>29.6 ± 2.8	Placebo vs. Magnesium (350 mg,q.d.)	no	yes	24	IL-6,TNF-α,CRP
Moslehi et al. [7]	2012	Iran	35	34	40-55	0	28.0 ± 3.2<br>28.1 ± 2.9	Placebo vs. Magnesium (250 mg, q.d.)	no	no	8	IL-6，CRP
Chacko et al. [8]	2010	America	7	7	47.0 ± 13.8<br>41.9 ± 12.7	69.2	28.3 ± 1.6<br>28.1 ± 2.2	Placebo vs. Magnesium (500 mg,q.d.)	no	no	4	IL-6,TNFα, CRP,Leptin
Talari et al. [9]	2019	Iran	27	27	61.8 ± 10.2<br>58.8 ± 10.1	46	27.2 ±5.6<br>26.1 ±4.5	Placebo vs. Magnesium (250 mg, q.d.)	no	no	24	CRP

Author	Year	Country	Sample		Age Mean(SD) /Range	Male %	BMI	Interventions	Exercise	Diet control	Duration (week)	Outcome
			T	C								
Abiri et al. [10]	2021	Iran	25/26/26	25	34.40 ± 9.39<br>34.15 ± 9.28<br>34.30 ± 9.24<br>34.36 ± 9.36	0	33.47 ±1.82<br>33.18 ±1.92<br>33.00 ±1.93<br>33.40 ±1.93	Placebo vs. Vitamin D (50,000 IU, q.w.) vs. Magnesium (250 mg, q.d.) vs.Vitamin D (50,000 IU, q.w.) + Magnesium (250 mg, q.d.)	no	no	8	IL-6,TNF-α, CRP,
Kheyruri et al. [11]	2020	Iran	42	41	45 ± 5.9<br>46.7 ± 6.7	0	27.6-35.05	Placebo vs. Vitamin D (50,000 IU, q.w.) + Magnesium (250 mg, q.d.)	no	no	8	IL-6,TNF-α, CRP
Agbalalah et al. [12]	2022	UK	28	27	35.9±11.8<br>33.1±12.2	100	29.9 ± 3.3<br>28.4 ± 2.6	Placebo vs. Vitamin D(125 μg, q.d.)	no	no	8	CRP
Cheung.et al. [13]	2022	America	21/34	23	41.0-45.3	35.9	25-40	Placebo vs. Vitamin D (1000 IU, t.i.d.) vs.Mg (360 mg, t.i.d.) + Vitamin D (1000 IU, t.i.d.)	no	yes	12	IL-6,CRP,Adiponectin
Duggan et al. [14]	2015	America	109	109	60.3 ± 5.3<br>59.0 ± 4.7	0	28.51 ±1.04<br>28.39 ±0.87	Placebo vs. Vitamin D (2000 IU, q.d.)	yes	yes	52	IL-6,TNF-α, Leptin,Adiponectin
Khodadoust et al. [15]	2021	Iran	13	13	45-55	100	33.7 ± 4.1<br>33.0 ± 4.3	Placebo vs. Vitamin D (50,000 IU,q.w,)	yes	no	8	TNF-α
Zittermann et al. [16]	2009	Germany	82	83	47.4±10.3<br>43.7±10.1	32.7	33.17 ±6.34<br>32.64 ±2.37	Placebo vs. Vitamin D (3,332IU,q.d.)	no	yes	52	IL-6,TNF-α, CRP
Khorsandi et al. [17]	2019	Iran	18	22	35.63 ± 3.2<br>32.95 ± 1.7	28	30-58.5<br>28.51 ±1.04	Placebo vs. Zinc (30 mg, q.d.)	no	yes	15	CRP
Santellano et al. [18]	2024	Mexico	8/9	7	NA	25	28.39 ±0.87	Placebo vs. Zinc (50 mg, q.d.) vs. Resveratrol (500 mg, q.d.)	no	no	8.5	Leptin

Author	Year	Country	Sample		Age Mean(SD) /Range	Male %	BMI	Interventions	Exercise	Diet control	Duration (week)	Outcome
			T	C								
Foster et al. [19]	2013	Australia	12/10	10	65.0 ± 7.8	0	28.6 ± 5.1	Placebo vs. Zinc (40 mg, q.d.) vs. α-Lipoic acid (2 g , q.d.)	no	no	12	IL-6,TNF-α,CRP
Kobyliak et al. [20]	2020	Ukraine	28	26	56.29 ± 11.14<br>55.73 ± 8.76	50	35.66 ± 5.35<br>35.63 ± 7.76	Placebo vs. Probiotic+omega-3 PUFA (1 sachet, q.d.)	no	no	8	IL-6,TNF-α
Laue et al. [21]	2023	Germany	60/60	60	61.50 ± 11.30<br>56.90 ± 14.60<br>61.80 ± 10.30	36.7	32.37 ± 5.18<br>31.98 ± 5.73<br>32.64 ± 5.50	Placebo vs. Probiotic (5×10^9 CFU, b.i.d.) vs. Synbiotic (10 g, q.d.)	no	no	12	CRP
Noori et al. [22]	2025	Iran	32	35	65-66	100	26.97 ± 2.46<br>27.34 ± 3.02	Placebo vs. Probiotic（3×10^9 CFU, q.d.)	no	no	8	IL-6,CRP
Tenorio-Jiménez et al. [23]	2019	Spain	25	28	NA	NA	38.30 ± 7.3<br>38.76 ± 7.2	Placebo vs. Probiotic(5×10^9 CFU, q.d.)	yes	yes	12	IL-6, TNF-α, CRP, Leptin, Adiponectin,
Nasiri et al. [24]	2021	Iran	21/21/21	21	34.67 ± 5.02<br>37.33 ± 7.64<br>34.86 ± 5.73<br>34.29 ± 7.25	NA	27.57 ± 0.77<br>27.26 ± 0.57<br>27.78 ± 0.98<br>27.87 ± 0.51	Placebo vs. Probiotic (500 mg, q.d.) vs. Alpha-Lipoic Acid (600 mg, q.d.) vs. Probiotic (500 mg, q.d.) + Alpha-Lipoic Acid (600 mg, q.d.)	no	yes	24	CRP
Rajkumar et al. [25]	2014	India	15/15/15	15	40–60	50	28.79 ± NA	Placebo vs. Probiotic (1.125×1011CFU, q.d.) vs. Omega-3 (180 mg EPA + 120 mg DHA, q.d.) vs.Probiotic(1.125×1011CFU, q.d.) + Omega-3(180 mg EPA + 120 mg DHA, q.d.)	no	no	6	CRP

**Author**	**Year**	**Country**	**Sample**		**Age Mean(SD) /Range**	**Male%**	**BMI**	**Interventions**	**Exercise**	**Diet control**	**Duration (week)**	**Outcome**
			**T**	**C**								
Farrokhian et al. [26]	2017	Iran	30	30	50-85	36.7	32.3 ± 6.0	Placebo vs. Synbiotic (2× 109CFU/g + 800 mg inulin)	no	no	12	CRP
Kanazawa et al. [27]	2021	Japan	44	42	61.1 ± 11.0<br>61.1 ± 11.0	70.5	29.6 ± 4.6<br>29.5 ± 4.4	Placebo vs. Synbiotic(3×10^8 CFU b.i.d.)	no	yes	24	IL-6
Lyon et al. [28]	2023	India	46	49	41.13 ± 6.58<br>41.74 ± 7.21	20.4	29.1 ± 3.7<br>31.15 ± 2.83	Placebo vs. Synbiotic(2×1010 CFU,q.d.)	no	no	12	CRP
Rabiei et al. [29]	2018	Iran	20	20	57.1 ± 1.5<br>60.8 ± 1.6	28.3	29.89 ± 2.59<br>32.7 ± 1.0	Placebo vs. Synbiotic (2×10^8 CFU, b.i.d.)	no	yes	12	IL-6,CRP
Raji Lahiji et al. [30]	2021	Iran	36	36	56.56 ± 5.07<br>58.31 ± 6.44	0	32.7 ± 1.0<br>NA	Placebo vs. Synbiotic (1×10^9 CFU, q.d.)	no	yes	8	CRP,Adiponectin
Ekhlasi et al. [31]	2017	Iran	15/15	15	25-64	80	27.28 ± 2.21	Placebo vs. Synbiotic (4×10^8 CFU, q.d.) vs. Vitamin E(400 IU, q.d.)	no	yes	52	TNF-α
Pauls et al. [32]	2021	Canada	21	22	21-51	0	36.8 ± 6.10<br>36.8 ± 6.10	α-Lipoic acid (4 g,q.d) vs. Omega-3 (3.86 g DHA + 1.01 g EPA, q.d.)	no	yes	4	IL-6,TNF-α,CRP, Adiponectin
Huerta et al. [33]	2015	Spain	19	21	38 ± 7<br>39 ± 8	0	27.5-40	Placebo vs. α-Lipoic acid (300 mg, q.d.)	no	yes	10	Leptin
Mohammadi et.al. [34].	2015	Iran	28	30	39 ± 6.44<br>36.8 ± 7.48	100	27.77 ± 4.33<br>28.02 ± 5.09	Placebo vs. α-Lipoic acid (600 mg, q.d.)	no	no	12	IL-6,CRP
Sardu et al. [35]	2017	Italy	33	40	58.8 ± 6.7<br>61.5 ± 8.1	52	29.6±4.8<br>28.9±2.5	Placebo vs. α-Lipoic acid (600 mg, q.d.)	no	no	52	IL-6,TNF-α, CRP

Author	Year	Country	Sample		Age Mean(SD) /Range	Male%	BMI	Interventions	Exercise	Diet control	Duration (week)	Outcome
			T	C								
Marfella et al. [36]	2015	Italy	22	21	63.7 ± 6.5<br>63.9 ± 5.2	0	27.0 ± 1.6<br>27.6 ± 2.2	Placebo vs. α-Lipoic acid (600 mg, q.d.)	no	no	52	TNF-α,CRP
Manning et al. [37]	2012	New Zealand	34/36	40	55±10<br>57±10<br>54±13	37.1	31.7 ± 6.2<br>31.5 ± 5.6<br>31.3 ± 5.3	Placebo vs. α-Lipoic acid (600 mg, q.d.) vs. Vitamin E (100 IU, q.d.)	no	yes	52	IL-6,TNF-α,CRP, Adiponectin
Zhang et al. [38]	2011	China	13	9	52.5 ± 8.2<br>52.6 ± 6.2	50	30.2 ± 1.4<br>30.4 ± 2.1	Placebo vs. α-Lipoic acid (600 mg, q.d.)	no	yes	2	IL-6,TNF-α,Adiponectin
Bakker et al. [39]	2023	Netherlands	26	30	44-49	0	41-43	Placebo vs. Omega-3(920 mg EPA + 760 mg DHA, q.d.)	no	yes	4	IL-6,CRP
Itariu et al. [40]	2012	Austria	27	28	39 ± 10.6<br>39 ± 10.6	14.8	43.5 ± 5.5<br>44.4 ± 5.4	Placebo vs. Omega-3(1840 mg EPA + 1520 mg DHA q.d.)	no	no	8	IL-6,CRP,Leptin, Adiponectin
Krebs et al. [41]	2006	UK	35	32	21-69	0	35.3 ± 5.6	Placebo vs. Omega-3 (1300mg EPA + 2900mg DHA, q.d.)	yes	yes	24	IL-6,TNF-α,CRP, Leptin,Adiponectin
Munro et al. [42]	2013	Australia	15	18	18-60	33	30-40	Placebo vs. Omega-3 (1620 mg DHA + 420 mg EPA,q.d)	no	yes	12	IL-6,TNF-α,CRP, Leptin,Adiponectin
Huerta et al. [43]	2015	Spain	16/19	21	20–50	0	27.5-40	Placebo vs. Omega-3(1300 mg, q.d.) vs. α-Lipoic acid (300 mg, q.d.)	no	yes	10	IL-6,CRP,Adiponectin
Hejazi et al. [44]	2015	Iran	14	13	48.0 ± 6.28<br>46.61 ± 7.58	25.9	29.22 ± 6.62<br>28.81 ± 4.04	Placebo vs. Vitamin E (400 IU, q.d.)	no	no	6	IL-6,CRP
Khosla et al. [45]	2013	America	41	40	59 ± 12<br>59 ± 12	53	30.3 ± 8.1<br>28.7 ± 8.2	Placebo vs. Vitamin E (220 mg ,q.d.)	no	no	16	IL-6

Author	Year	Country	Sample		Age Mean(SD) /Range	Male%	BMI	Interventions	Exercise	Diet control	Duration (week)	Outcome
			T	C								
Devaraj et al. [46]	2008	America	20	20	57 ± 14<br>56±11	30	35 ± 7<br>39 ± 15	Placebo vs. Vitamin E(800 mg α-Tocopherol + 800 mg γ-Tocopherol, q.d.)	no	no	6	IL-6,TNF-α
Stonehouse et al. [47]	2016	Australia	28	29	18-70	57	30.7-34.8	Placebo vs. Vitamin E (132 mg α-Tocopherol + 420 mg γ-Tocophero, q.d.)	no	yes	8	TNF-α
Pervez et al. [48]	2018	Pakistan	31	33	44.7 ± 8.8<br>43.9 ± 9.2	50	30.68 ± 2.9<br>31.30 ± 3.1	Placebo vs. Vitamin E(600mg,q.d.)	yes	yes	12	CRP
Bagheri et al. [49]	2020	Iran	15	15	43.8±3.4<br>44.6±3.0	100	27.3 ± 0.4<br>27.8 ± 0.3	Placebo vs. Green tea extract (500 mg, q.d.)	yes	yes	8	TNF-α,CRP Adiponectin
de Oliveira Vogado et al. [50]	2024	Brazil	32	29	20-60	0	27.5-28.9	Placebo vs. Green tea extract (558 mg, q.d.)	no	yes	12	IL-6
Nogueira et al. [51]	2016	Brazil	20	20	41.1 ± 8.4<br>41.1 ± 8.4	0	33.56 ± 0.98<br>33.56 ± 0.98	Placebo vs. Green tea extract (1500 mg, q.d.)	no	no	4	IL-6,TNF-α,CRP, Adiponectin
Bogdanski et al [52]	2012	Poland	28	28	51.5 ± 7.4<br>49.2 ± 8.8	50	32.5 ± 3.3<br>33.9 ± 2.3	Placebo vs. Green tea extract (379 mg,q.d.)	no	yes	12	TNF-α,CRP
Basu et al. [53]	2010	America	7	11	39.5 ± 3.0<br>44.6 ± 3.2	41.7	36.1 ± 1.3<br>36.1 ± 1.3	Placebo vs. Green tea extract (460 mg,q.d.)	no	yes	8	CRP,Leptin,Adiponectin
Cunningham et al. [54]	2026	America	50	47	60.4± 5.3<br>60.4± 5.5	0	29.1 ± 3.1<br>28.7 ± 2.7	Placebo vs. Green tea extract (843 mg , q.d.)	no	no	52	IL-6,TNF-α,CRP
Campbell et al. [55]	2019	America	10	10	18-35	NA	NA	Placebo vs. Curcumin (500 mg, q.d.)	no	no	12	Adiponectin
Ganjali et al. [56]	2014	Iran	42	42	18-65	NA	NA	Placebo vs. Curcumin (1000 mg , q.d.)	no	no	4	IL-6,TNF-α

Author	Year	Country	Sample		Age Mean(SD) /Range	Male%	BMI	Interventions	Exercise	Diet control	Duration (week)	Outcome
			T	C								
Hellmann et al. [57]	2022	Denmark	18	19	46 ± 14	73	38.0 ± 5.5<br>39.5 ± 6.8	Placebo vs. Curcumin (400 mg, q.d.)	no	no	6	IL-6,TNF-α,CRP, Adiponectin
Jazayeri-Tehrani et al. [58]	2019	Iran	42	42	41.8 ± 5.6<br>42.5 ± 6.2	54.8	30.67 ± 2.14<br>30.75 ± 2.35	Placebo vs. Curcumin (80 mg, q.d.)	yes	yes	12	IL-6,TNF-α,CRP
Sohaei et al. [59]	2019	Iran	27	24	29.40 ± 5.33<br>29.58 ± 5	0	29.67 ± 3.72<br>31.32 ± 4.60	Placebo vs. Curcumin (1000 mg,q.d.)	no	no	6	CRP
Yaikwawong et al. [60]	2024	Thailand	113	114	60.27 ± 0.83<br>62.26 ± 0.81	51.1	27.21 ± 0.37<br>26.76 ± 0.38	Placebo vs.Curcumin (1500 mg, q.d.)	yes	yes	48	IL-6,TNF-α

Supplementary File S3
List of included studies.

1. Walker, J.M.; Eckardt, P.; Aleman, J.O.; da Rosa, J.C.; Liang, Y.; Iizumi, T.; Etheve, S.; Blaser, M.J.; L Breslow, J.; Holt, P.R. The Effects of Trans-Resveratrol on Insulin Resistance, Inflammation, and Microbiota in Men with the Metabolic Syndrome: A Pilot Randomized, Placebo-Controlled Clinical Trial. *J Clin Transl Res* **2018**.
2. Arzola-Paniagua, M.A.; García-Salgado López, E.R.; Calvo-Vargas, C.G.; Guevara-Cruz, M. Efficacy of an Orlistat-Resveratrol Combination for Weight Loss in Subjects with Obesity: A Randomized Controlled Trial. *Obesity* **2016**. https://doi.org/10.1002/oby.21523.
3. Kantartzis, K.; Fritsche, L.; Bombrich, M.; Machann, J.; Schick, F.; Staiger, H.; Kunz, I.; Schoop, R.; Lehn-Stefan, A.; Heni, M.; et al. Effects of Resveratrol Supplementation on Liver Fat Content in Overweight and Insulin Resistant Subjects: A Randomized, Double-Blind and Placebo-Controlled Clinical Trial. *Diabetes Obes. Metab.* **2018**. https://doi.org/10.1111/dom.13268.
4. Poulsen, M.M.; Vestergaard, P.F.; Clasen, B.F.; Radko, Y.; Christensen, L.P.; Stødkilde-Jørgensen, H.; Møller, N.; Jessen, N.; Pedersen, S.B.; Jørgensen, J.O.L. High-Dose Resveratrol Supplementation in Obese Men: An Investigator-Initiated, Randomized, Placebo-Controlled Clinical Trial of Substrate Metabolism, Insulin Sensitivity, and Body Composition. *Diabetes* **2012**. https://doi.org/10.2337/db12-0975.
5. Seyyedebrahimi, S.; Khodabandehloo, H.; Nasli Esfahani, E.; Meshkani, R. The Effects of Resveratrol on Markers of Oxidative Stress in Patients with Type 2 Diabetes: A Randomized, Double-Blind, Placebo-Controlled Clinical Trial. *Acta Diabetol.* **2018**. https://doi.org/10.1007/s00592-017-1098-3.
6. Joris, P.J.; Plat, J.; Bakker, S.J.L.; Mensink, R.P. Effects of Long-Term Magnesium Supplementation on Endothelial Function and Cardiometabolic Risk Markers: A Randomized Controlled Trial in Overweight/Obese Adults. *Sci. Rep.* **2017**. https://doi.org/10.1038/s41598-017-00205-9.
7. Moslehi, N.; Vafa, M.; Rahimi-Foroushani, A.; Golestan, B. Effects of Oral Magnesium Supplementation on Inflammatory Markers in Middle-Aged Overweight Women. *J Res Med Sci* **2012**.
8. Chacko, S.A.; Sul, J.; Song, Y.; Li, X.; LeBlanc, J.; You, Y.; Butch, A.; Liu, S. Magnesium Supplementation, Metabolic and Inflammatory Markers, and Global Genomic and Proteomic Profiling: A Randomized, Double-Blind, Controlled, Crossover Trial in Overweight Individuals. *Am. J. Clin. Nutr.* **2010**. https://doi.org/10.3945/ajcn.110.002949.
9. Talari, H.R.; Zakizade, M.; Soleimani, A.; Bahmani, F.; Ghaderi, A.; Mirhosseini, N.; Eslahi, M.; Babadi, M.; Mansournia, M.A.; Asemi, Z. Effects of Magnesium Supplementation on Carotid Intima–Media Thickness and Metabolic Profiles in Diabetic Haemodialysis Patients: A Randomised, Double-Blind, Placebo-Controlled Trial. *Briti. J. Nutr.* **2019**. https://doi.org/10.1017/s0007114519000163.
10. Abiri, B.; Sarbakhsh, P.; Vafa, M. Randomized Study of the Effects of Vitamin D and/or Magnesium Supplementation on Mood, Serum Levels of BDNF, Inflammation, and SIRT1 in Obese Women with Mild to Moderate Depressive Symptoms. *Nutr. Neurosci.* **2021**. https://doi.org/10.1080/1028415x.2021.1945859.
11. Kheyruri, F.; Sarrafzadeh, J.; Hosseini, A.F.; Abiri, B.; Vafa, M. Randomized Study of the Effects of Vitamin D and Magnesium Co-Supplementation on Muscle Strength and Function, Body Composition, and Inflammation in Vitamin D-Deficient Middle-Aged Women. *Biol. Trace Elem. Res.* **2020**. https://doi.org/10.1007/s12011-020-02387-2.
12. Agbalalah, T.; Mushtaq, S. Effect of Vitamin D3 Supplementation on Cardiometabolic Disease Risk among Overweight/Obese Adult Males in the UK: A Pilot Randomised Controlled Trial. *J. Hum. Nutr. Diet.* **2022**. https://doi.org/10.1111/jhn.13021.
13. Cheung, M.M.; Dall, R.D.; Shewokis, P.A.; Altasan, A.; Volpe, S.L.; Amori, R.; Singh, H.; Sukumar, D. The Effect of Combined Magnesium and Vitamin D Supplementation on Vitamin D Status, Systemic Inflammation, and Blood Pressure: A Randomized Double-Blinded Controlled Trial. *Nutrition* **2022**. https://doi.org/10.1016/j.nut.2022.111674.
14. Duggan, C.; de Dieu Tapsoba, J.; Mason, C.; Imayama, I.; Korde, L.; Wang, C.-Y.; McTiernan, A. Effect of Vitamin D3 Supplementation in Combination with Weight Loss on Inflammatory Biomarkers in Postmenopausal Women: A Randomized Controlled Trial. *Cancer Prev. Res.* **2015**. https://doi.org/10.1158/1940-6207.capr-14-0449.

15. Khodadoust, M.; Habibian, M. Investigating the Changes of Tumor Necrosis Factor-α and Interleukin-10 After 8 Weeks of Regular Pilates Exercise and Vitamin D Intake in Overweight Men: A Randomized Clinical Trial. *Arak Medical University Journal* **2021**, *23*, 888–900. https://doi.org/10.32598/JAMS.23.6.3537.5.
16. Zittermann, A.; Frisch, S.; Berthold, H.K.; Götting, C.; Kuhn, J.; Kleesiek, K.; Stehle, P.; Koertke, H.; Koerfer, R. Vitamin D Supplementation Enhances the Beneficial Effects of Weight Loss on Cardiovascular Disease Risk Markers. *Am. J. Clin. Nutr.* **2009**. https://doi.org/10.3945/ajcn.2008.27004.
17. Khorsandi, H.; Nikpayam, O.; Yousefi, R.; Parandoosh, M.; Hosseinzadeh, N.; Saidpour, A.; Ghorbani, A. Zinc Supplementation Improves Body Weight Management, Inflammatory Biomarkers and Insulin Resistance in Individuals with Obesity: A Randomized, Placebo-Controlled, Double-Blind Trial. *Diabetol. Metab. Syndr.* **2019**. https://doi.org/10.1186/s13098-019-0497-8.
18. Santellano-Estrada, E.; Portillo Siqueiros, E.Y.; Flores Villalobos, M.Á.; Roacho Soto, M.G.; Martínez Flórez, S. [Effects of Zinc and Resveratrol as Modulators of Leptin Response in Adults with Obesity]. *Nutr Hosp* **2024**. https://doi.org/10.20960/nh.05177.
19. Foster, M.; Petocz, P.; Samman, S. Inflammation Markers Predict Zinc Transporter Gene Expression in Women with Type 2 Diabetes Mellitus. *J. Nutr. Biochem.* **2013**. https://doi.org/10.1016/j.jnutbio.2013.02.006.
20. Kobyliak, N.; Falalyeyeva, T.; Mykhalchyshyn, G.; Molochek, N.; Savchuk, O.; Kyriienko, D.; Komisarenko, I. Probiotic and Omega-3 Polyunsaturated Fatty Acids Supplementation Reduces Insulin Resistance, Improves Glycemia and Obesity Parameters in Individuals with Type 2 Diabetes: A Randomised Controlled Trial. *Obesity Medicine* **2020**. https://doi.org/10.1016/j.obmed.2020.100248.
21. Laue, C.; Papazova, E.; Pannenbeckers, A.; Schrezenmeir, J. Effect of a Probiotic and a Synbiotic on Body Fat Mass, Body Weight and Traits of Metabolic Syndrome in Individuals with Abdominal Overweight: A Human, Double-Blind, Randomised, Controlled Clinical Study. *Nutrients* **2023**. https://doi.org/10.3390/nu15133039.
22. Noori, M.; Shateri, Z.; Babajafari, S.; Eskandari, M.H.; Parastouei, K.; Ghasemi, M.; Afshari, H.; Samadi, M. The Effect of Probiotic-Fortified Kefir on Depression, Appetite, Oxidative Stress, and Inflammatory Parameters in Iranian Overweight and Obese Elderly: A Randomized, Double-Blind, Placebo-Controlled Clinical Trial. *J. Health Popul. Nutr.* **2025**. https://doi.org/10.1186/s41043-025-00773-x.
23. Tenorio-Jiménez, C.; Martínez-Ramírez, M.J.; Del Castillo-Codes, I.; Arraiza-Irigoyen, C.; Tercero-Lozano, M.; Camacho, J.; Chueca, N.; García, F.; Olza, J.; Plaza-Díaz, J.; et al. Lactobacillus Reuteri V3401 Reduces Inflammatory Biomarkers and Modifies the Gastrointestinal Microbiome in Adults with Metabolic Syndrome: The PROSIR Study. *Nutrients* **2019**. https://doi.org/10.3390/nu11081761.
24. Nasiri, G.; Bastani, A.; Haji-Aghamohammadi, A.A.; Nooshabadi, M.R.; Shahmirzalou, P.; Haghighian, H.K. Effects of Probiotic and Alpha-Lipoic Acid Supplements, Separately or in Combination on the Anthropometric Indicators and Maintenance of Weight in Overweight Individuals. *Clinical Nutrition ESPEN* **2021**. https://doi.org/10.1016/j.clnesp.2020.12.007.
25. Rajkumar, H.; Mahmood, N.; Kumar, M.; Varikuti, S.R.; Challa, H.R.; Myakala, S.P. Effect of Probiotic (VSL#3) and Omega-3 on Lipid Profile, Insulin Sensitivity, Inflammatory Markers, and Gut Colonization in Overweight Adults: A Randomized, Controlled Trial. *Mediat. Inflamm.* **2014**. https://doi.org/10.1155/2014/348959.
26. Farrokhian, A.; Raygan, F.; Soltani, A.; Tajabadi-Ebrahimi, M.; Sharifi Esfahani, M.; Karami, A.A.; Asemi, Z. The Effects of Synbiotic Supplementation on Carotid Intima-Media Thickness, Biomarkers of Inflammation, and Oxidative Stress in People with Overweight, Diabetes, and Coronary Heart Disease: A Randomized, Double-Blind, Placebo-Controlled Trial. *Probiotics Antimicrob. Proteins* **2017**. https://doi.org/10.1007/s12602-017-9343-1.
27. Kanazawa, A.; Aida, M.; Yoshida, Y.; Kaga, H.; Katahira, T.; Suzuki, L.; Tamaki, S.; Sato, J.; Goto, H.; Azuma, K.; et al. Effects of Synbiotic Supplementation on Chronic Inflammation and the Gut Microbiota in Obese Patients with Type 2 Diabetes Mellitus: A Randomized Controlled Study. *Nutrients* **2021**. https://doi.org/10.3390/nu13020558.
28. Lyon, J.; Connell, M.; Chandrasekaran, K.; Srivastava, S. Effect of Synbiotics on Weight Loss and Metabolic Health in Adults with Overweight and Obesity: A Randomized

Controlled Trial. *Obesity* **2023**. https://doi.org/10.1002/oby.23801.
29. Rabiei, S.; Hedayati, M.; Rashidkhani, B.; Saadat, N.; Shakerhossini, R. The Effects of Synbiotic Supplementation on Body Mass Index, Metabolic and Inflammatory Biomarkers, and Appetite in Patients with Metabolic Syndrome: A Triple-Blind Randomized Controlled Trial. *Journal of Dietary Supplements* **2018**. https://doi.org/10.1080/19390211.2018.1455788.
30. Raji Lahiji, M.; Zarrati, M.; Najafi, S.; Yazdani, B.; Cheshmazar, E.; Razmpoosh, E.; Janani, L.; Raji Lahiji, M.; Shidfar, F. Effects of Synbiotic Supplementation on Serum Adiponectin and Inflammation Status of Overweight and Obese Breast Cancer Survivors: A Randomized, Triple-Blind, Placebo-Controlled Trial. *Support. Care Cancer* **2021**. https://doi.org/10.1007/s00520-020-05926-8.
31. Ekhlasi, G.; Zarrati, M.; Agah, S.; Hosseini, A.F.; Hosseini, S.; Shidfar, S.; Soltani Aarbshahi, S.S.; Razmpoosh, E.; Shidfar, F. Effects of Symbiotic and Vitamin E Supplementation on Blood Pressure, Nitric Oxide and Inflammatory Factors in Non-Alcoholic Fatty Liver Disease. *EXCLI J* **2017**. https://doi.org/10.17179/excli2016-846.
32. Pauls, S.D.; Rodway, L.R.; Sidhu, K.K.; Winter, T.; Sidhu, N.; Aukema, H.M.; Zahradka, P.; Taylor, C.G. Oils Rich in α-Linolenic Acid or Docosahexaenoic Acid Have Distinct Effects on Plasma Oxylipin and Adiponectin Concentrations and on Monocyte Bioenergetics in Women with Obesity. *J. Nutr.* **2021**. https://doi.org/10.1093/jn/nxab235.
33. Huerta, A.E.; Navas-Carretero, S.; Prieto-Hontoria, P.L.; Martínez, J.A.; Moreno-Aliaga, M.J. Effects of α-Lipoic Acid and Eicosapentaenoic Acid in Overweight and Obese Women during Weight Loss. *Obesity* **2015**. https://doi.org/10.1002/oby.20966.
34. Mohammadi, V.; Khalili, M.; Eghtesadi, S.; Dehghani, S.; Jazayeri, S.; Aghababaee, S.K.; Sabour, H.; Saberi, H.; Eghtesadi, M.; Gohari, M.R. The Effect of Alpha-Lipoic Acid (ALA) Supplementation on Cardiovascular Risk Factors in Men with Chronic Spinal Cord Injury: A Clinical Trial. *Spinal Cord*. https://doi.org/10.1038/sc.2015.35.
35. Sardu, C.; Santulli, G.; Santamaria, M.; Barbieri, M.; Sacra, C.; Paolisso, P.; D'Amico, F.; Testa, N.; Caporaso, I.; Paolisso, G.; et al. Effects of Alpha Lipoic Acid on Multiple Cytokines and Biomarkers and Recurrence of Atrial Fibrillation Within 1 Year of Catheter Ablation. *Am J Cardiol* **2017**. https://doi.org/10.1016/j.amjcard.2017.01.040.
36. Marfella, R.; Barbieri, M.; Sardu, C.; Rizzo, M.R.; Siniscalchi, M.; Paolisso, P.; Ambrosino, M.; Fava, I.; Materazzi, C.; Cinquegrana, G.; et al. Effects of α-Lipoic Acid Therapy on Sympathetic Heart Innervation in Patients with Previous Experience of Transient Takotsubo Cardiomyopathy. *J. Cardiol.* **2015**. https://doi.org/10.1016/j.jjcc.2015.07.012.
37. Manning, P.J.; Sutherland, W.H.F.; Williams, S.M.; Walker, R.J.; Berry, E.A.; De Jong, S.A.; Ryalls, A.R. The Effect of Lipoic Acid and Vitamin E Therapies in Individuals with the Metabolic Syndrome. *Nutr. Metab. Cardiovascular Dis.* **2012**. https://doi.org/10.1016/j.numecd.2011.11.006.
38. Zhang, Y.; Han, P.; Wu, N.; He, B.; Lu, Y.; Li, S.; Liu, Y.; Zhao, S.; Liu, L.; Li, Y. Amelioration of Lipid Abnormalities by α-Lipoic Acid through Antioxidative and Anti-Inflammatory Effects. *Obesity* **2011**. https://doi.org/10.1038/oby.2011.121.
39. Bakker, N.; Hickey, M.; Shams, R.; Rivera, C.F.; Vlahos, J.; Cense, H.A.; Demirkiran, A.; Ramkhelawon, B.; Houdijk, A.P. Oral ω-3 PUFA Supplementation Modulates Inflammation in Adipose Tissue Depots in Morbidly Obese Women: A Randomized Trial. *Nutrition* **2023**. https://doi.org/10.1016/j.nut.2023.112055.
40. Itariu, B.K.; Zeyda, M.; Hochbrugger, E.E.; Neuhofer, A.; Prager, G.; Schindler, K.; Bohdjalian, A.; Mascher, D.; Vangala, S.; Schranz, M.; et al. Long-Chain n-3 PUFAs Reduce Adipose Tissue and Systemic Inflammation in Severely Obese Nondiabetic Patients: A Randomized Controlled Trial. *Am. J. Clin. Nutr.* **2012**. https://doi.org/10.3945/ajcn.112.037432.
41. Krebs, J.D.; Browning, L.M.; McLean, N.K.; Rothwell, J.L.; Mishra, G.D.; Moore, C.S.; Jebb, S.A. Additive Benefits of Long-Chain n-3 Polyunsaturated Fatty Acids and Weight-Loss in the Management of Cardiovascular Disease Risk in Overweight Hyperinsulinaemic Women. *Int. J. Obesity* **2006**. https://doi.org/10.1038/sj.ijo.0803309.
42. Munro, I.A.; Garg, M.L. Dietary Supplementation with Long Chain Omega-3 Polyunsaturated Fatty Acids and Weight Loss in Obese Adults. *Obes. Res. Clin. Pract.* **2013**. https://doi.org/10.1016/j.orcp.2011.11.001.
43. Huerta, A.E.; Prieto-Hontoria, P.L.; Sáinz, N.; Martínez, J.A.; Moreno-Aliaga, M.J. Supplementation with α-Lipoic Acid Alone or in Combination with Eicosapentaenoic Acid

Modulates the Inflammatory Status of Healthy Overweight or Obese Women Consuming an Energy-Restricted Diet. *J. Nutr.* **2015**. https://doi.org/10.3945/jn.115.224105.
44. Hejazi, N.; Dabbaghmanesh, M.H.; Mazloom, Z.; Dashtabi, A. Effects of Vitamin E on Fasting and Postprandial Oxidative Stress, Inflammatory Markers, Glucose Status, Insulin Resistance, Blood Pressure and Pulse Rate in Type-2 Diabetic Patients: A Randomized Clinical Trial. *Galen Medical Journal* **2015**, *4*, 67–74.
45. Khosla, P.; Daud, Z.; Tubie, B.; Sheyman, M.; Osia, R.; Adams, J.; Tubie, S. Vitamin E Tocotrienol Supplementation Improves Lipid Profiles in Chronic Hemodialysis Patients. *Vascular Health and Risk Management* **2013**. https://doi.org/10.2147/vhrm.s51710.
46. Devaraj, S.; Leonard, S.; Traber, M.G.; Jialal, I. Gamma-Tocopherol Supplementation Alone and in Combination with Alpha-Tocopherol Alters Biomarkers of Oxidative Stress and Inflammation in Subjects with Metabolic Syndrome. *Free Radical Bio. Med.* **2008**. https://doi.org/10.1016/j.freeradbiomed.2007.12.018.
47. Stonehouse, W.; Brinkworth, G.D.; Thompson, C.H.; Abeywardena, M.Y. Short Term Effects of Palm-Tocotrienol and Palm-Carotenes on Vascular Function and Cardiovascular Disease Risk: A Randomised Controlled Trial. *Atherosclerosis* **2016**. https://doi.org/10.1016/j.atherosclerosis.2016.10.027.
48. Pervez, M.A.; Khan, D.A.; Ijaz, A.; Khan, S. Effects of Delta-Tocotrienol Supplementation on Liver Enzymes, Inflammation, Oxidative Stress and Hepatic Steatosis in Patients with Nonalcoholic Fatty Liver Disease. *Turk J Gastroenterol* **2018**. https://doi.org/10.5152/tjg.2018.17297.
49. Bagheri, R.; Rashidlamir, A.; Ashtary-Larky, D.; Wong, A.; Grubbs, B.; Motevalli, M.S.; Baker, J.S.; Laher, I.; Zouhal, H. Effects of Green Tea Extract Supplementation and Endurance Training on Irisin, pro-Inflammatory Cytokines, and Adiponectin Concentrations in Overweight Middle-Aged Men. *Eur. J. Appl. Physiol.* **2020**. https://doi.org/10.1007/s00421-020-04332-6.
50. de Oliveira Vogado, C.; Ferreira, M.A.; Nakano, E.Y.; Azevedo, S.; Magalhães, K.G.; Fernandes Arruda, S.; Botelho, P.B. Interaction between Green Tea and Metformin and Its Effects on Oxidative Stress and Inflammation in Overweight Women: A Randomised Clinical Trial. *Briti. J. Nutr.* **2024**. https://doi.org/10.1017/s0007114524002356.
51. Nogueira, L. de P.; Nogueira Neto, J.F.; Klein, M.R.S.T.; Sanjuliani, A.F. Short-Term Effects of Green Tea on Blood Pressure, Endothelial Function, and Metabolic Profile in Obese Prehypertensive Women: A Crossover Randomized Clinical Trial. *J. Am. Coll. Nutr.* **2016**. https://doi.org/10.1080/07315724.2016.1194236.
52. Bogdanski, P.; Suliburska, J.; Szulinska, M.; Stepien, M.; Pupek-Musialik, D.; Jablecka, A. Green Tea Extract Reduces Blood Pressure, Inflammatory Biomarkers, and Oxidative Stress and Improves Parameters Associated with Insulin Resistance in Obese, Hypertensive Patients. *Nutr. Res.* **2012**. https://doi.org/10.1016/j.nutres.2012.05.007.
53. Basu, A.; Du, M.; Sanchez, K.; Leyva, M.J.; Betts, N.M.; Blevins, S.; Wu, M.; Aston, C.E.; Lyons, T.J. Green Tea Minimally Affects Biomarkers of Inflammation in Obese Subjects with Metabolic Syndrome. *Nutrition* **2010**. https://doi.org/10.1016/j.nut.2010.01.015.
54. Cunningham, A.; Gomes, A.; Meng, L.; Shapses, S.; Byham-Gray, L.; Samavat, H. Effects of Green Tea Extract Supplementation on Inflammatory Cytokines Among Postmenopausal Women with Overweight or Obesity—A Secondary Analysis of a Randomized Controlled Trial. *Nutrients* **2026**. https://doi.org/10.3390/nu18010143.
55. Campbell, M.S.; Ouyang, A.; I.M., K.; Charnigo, R.J.; Westgate, P.M.; Fleenor, B.S. Influence of Enhanced Bioavailable Curcumin on Obesity-Associated Cardiovascular Disease Risk Factors and Arterial Function: A Double-Blinded, Randomized, Controlled Trial. *Nutrition* **2019**. https://doi.org/10.1016/j.nut.2019.01.002.
56. Ganjali, S.; Sahebkar, A.; Mahdipour, E.; Jamialahmadi, K.; Torabi, S.; Akhlaghi, S.; Ferns, G.; Parizadeh, S.M.R.; Ghayour-Mobarhan, M. Investigation of the Effects of Curcumin on Serum Cytokines in Obese Individuals: A Randomized Controlled Trial. *The Scientific World Journal* **2014**. https://doi.org/10.1155/2014/898361.
57. Hellmann, P.H.; Bagger, J.I.; Carlander, K.R.; Forman, J.; Chabanova, E.; Svenningsen, J.S.; Holst, J.J.; Gillum, M.P.; Vilsbøll, T.; Knop, F.K. The Effect of Curcumin on Hepatic Fat Content in Individuals with Obesity. *Diabetes Obes. Metab.* **2022**. https://doi.org/10.1111/dom.14804.
58. Jazayeri-Tehrani, S.A.; Rezayat, S.M.; Mansouri, S.; Qorbani, M.; Alavian, S.M.; Daneshi-Maskooni, M.; Hosseinzadeh-Attar, M.-J. Nano-Curcumin Improves Glucose Indices, Lipids, Inflammation, and Nesfatin in Overweight and Obese Patients with Non-Alcoholic Fatty Liver Disease (NAFLD): A Double-Blind Randomized Placebo-Controlled Clinical Trial. *Nutr. Metab.* **2019**. https://doi.org/10.1186/s12986-019-0331-1.

59. Sohaei, S.; Amani, R.; Tarrahi, M.J.; Ghasemi-Tehrani, H. The Effects of Curcumin Supplementation on Glycemic Status, Lipid Profile and Hs-CRP Levels in Overweight/Obese Women with Polycystic Ovary Syndrome: A Randomized, Double-Blind, Placebo-Controlled Clinical Trial. *Complement Ther. Med.* **2019**. https://doi.org/10.1016/j.ctim.2019.102201.
60. Yaikwawong, M.; Jansarikit, L.; Jirawatnotai, S.; Chuengsamarn, S. Curcumin Reduces Depression in Obese Patients with Type 2 Diabetes: A Randomized Controlled Trial. *Nutrients* **2024**. https://doi.org/10.3390/nu16152414.

Supplementary Table S2

Pairwise meta-analysis

Comparisons	Studies	Participants	Heterogeneity%	Mean diff. (95% CI)
		**IL-6**		
Resveratrol vs Placebo	**2**	**129**	(P=0.13), I^2=56.27	-0.142 (-0.966, 0.683)
Mg vs Placebo	**4**	**197**	(P=0.073), I^2=56.46	0.053 (-0.162, 0.267)
VD＋Mg vs Placebo	**3**	**179**	(P=0.607, I^2=0	-0.110 (-0.348, 0.127)
Zinc vs Placebo	**1**	**22**	NA	-0.140 (-0.454, 0.174)
omega-3+Probiotics vs Placebo	**1**	**54**	NA	0.980 (-4.209, 6.169)
Probiotics vs Placebo	**3**	**120**	(P=0.008), I^2=78.47	-0.854 (-1.792, 0.084)
VD vs Placebo	**4**	**476**	(P=0.863), I^2=0	-0.030 (-0.350, 0.291)
α-lipoic acid vs Placebo	**7**	**328**	(P<0.001), I^2=90.34%	-0.319 (-0.789, 0.151)
Synbiotic vs Placebo	**2**	**126**	(P=0.757), I^2=0	-0.026 (-0.286, 0.234)
omega-3 vs Placebo	**6**	**289**	(P=0.567), I^2=0	-0.259 (-0.506,-0.013)
VE vs Placebo	**4**	**222**	(P=0.869), I^2=0	-0.295 (-0.914, 0.324)
Green tea vs Placebo	**3**	**154**	(P=0.973), I^2=0	-0.946 (-2.299, 0.406)
Curcumin vs Placebo	**3**	**151**	(P=0.102), I^2=69.6%	-0.285 (-0.971, 0.402)

Comparisons	Studies	Participants	Heterogeneity	Mean diff. (95% CI)
		**TNF-a**		
Resveratrol vs Placebo	**1**	**24**	NA	-0.860 (-4.725, 3.005)
Mg vs Placebo	**3**	**128**	(P=0.220), I^2=36.10	-0.099 (-0.757, 0.559)
VD＋Mg vs Placebo	**1**	**50**	NA	-1.060 (-1.983, -0.137)
Zinc vs Placebo	**1**	**22**	NA	0.700 (-0.178, 1.578)
omega-3+Probiotics vs Placebo	**1**	**54**	NA	-6.63(-11.916,-1.344)
Probiotics vs Placebo	**1**	**53**	NA	-1.54 (-2.476, -0.604)
VD vs Placebo	**5**	**527**	(P=0.074), I^2=55.04	-0.403 (-0.956, 0.149)
α-lipoic acid vs Placebo	**6**	**271**	(P<0.001), I^2=93.28	-0.804 (-1.850, 0.242)
Synbiotic vs Placebo	**1**	**30**	NA	-11.04(-20.488,-1.592)
omega-3 vs Placebo	**3**	**142**	(P=0.008), I^2=79.68	-0.653 (-1.936, 0.630)
VE vs Placebo	**3**	**171**	(P<0.001), I^2=92.75	-0.139 (-0.447, 0.168)
Green tea vs Placebo	**4**	**266**	(P=0.789), I^2=0.00	-0.119 (-0.429, 0.191)
Curcumin vs Placebo	**4**	**374**	(P<0.001), I^2=88.51	-0.222 (-0.542, 0.097)

Comparisons	Studies	Participants	Heterogeneity	Mean diff. (95% CI)
**CRP**				
Resveratrol vs Placebo	**4**	**205**	(P=0.402), I²=0.00	0.421 (-0.520, 1.362)
Mg vs Placebo	**5**	**254**	(P=0.228), I²=0.00	-0.052 (-0.258,0.153)
VD＋Mg vs Placebo	**3**	**180**	(P<0.001),I²=92.85	-0.343 (-0.825,0.139)
Zinc vs Placebo	**2**	**60**	(P=0.599), I²=0.00	-0.936(-1.473,-0.399)
α-lipoic acid +Probiotics vs Placebo	**1**	**42**	**NA**	-0.270(-0.319,-0.221)
omega-3+Probiotics vs Placebo	**1**	**30**	**NA**	-0.070 (-1.752, 1.612)
Probiotics vs Placebo	**5**	**302**	(P=0.460), I²=0.00	-0.210 (-0.251,-0.168)
VD vs Placebo	**4**	**332**	(P=0.407), I²=24.92	-0.004 (-0.126, 0.118)
α-lipoic acid vs Placebo	**8**	**376**	(P<0.001), I²=98.36	-0.343 (-0.924, 0.238)
Synbiotic vs Placebo	**5**	**379**	(P=0.180), I²=40.12	-0.157 (-0.480, 0.166)
omega-3 vs Placebo	**7**	**320**	(P=0.287), I²=23.53	-0.252 (-0.865, 0.360)
VE vs Placebo	**3**	**160**	(P=0.948), I²=0.00	-0.617 (-1.265, 0.030)
Green tea vs Placebo	**5**	**240**	(P=0.316), I²=8.52	-0.808 (-1.174,-0.442)
Curcumin vs Placebo	**3**	**174**	(P=0.006), I²=86.82	-0.774 (-2.351, 0.803)

Comparisons	Studies	Participants	Heterogeneity	Mean diff. (95% CI)
**Leptin**				
Resveratrol vs Placebo	**3**	**79**	(P=0.714), I²=0.00%	0.941 (-4.629, 6.512)
Mg vs Placebo	**1**	**26**	**NA**	-0.12(-4.867, 4.627)
Zinc vs Placebo	**1**	**15**	**NA**	16.10 (-13.063, 45.263)
Probiotics vs Placebo	**1**	**53**	**NA**	-10.040(-16.416,-3.664)
VD vs Placebo	**1**	**201**	**NA**	-1.12 (-6.352, 4.112)
α-lipoic acid vs Placebo	**1**	**40**	**NA**	-3.40 (-7.985, 1.185)
omega-3 vs Placebo	**3**	**155**	(P=0.571), I²=0.00	2.043 (-2.396, 6.482)
Green tea vs Placebo	**1**	**18**	**NA**	-6.90 (-31.726,17.926)

Comparisons	Studies	Participants	Heterogeneity	Mean diff. (95% CI)
**Adiponectin**				
Resveratrol vs Placebo	**2**	**144**	(P=0.758), I²=0.00	-0.085 (-1.488, 1.317)
VD＋Mg vs Placebo	**1**	**46**	**NA**	-0.010 (-0.216, 0.196)
Probiotics vs Placebo	**1**	**53**	**NA**	1.380 (-0.828, 3.588)
VD vs Placebo	**2**	**150**	(P=0.920), I²=0.00	0.081 (-0.126, 0.287)
α-lipoic acid vs Placebo	**4**	**177**	(P<0.001), I²=88.38	0.391 (-1.614, 2.396)
Synbiotic vs Placebo	**1**	**72**	**NA**	5.020 (-2.175, 12.215)

omega-3 vs Placebo	**5**	**235**	(P=0.227), I^2=35.49	-0.434 (-1.755, 0.887)
VE vs Placebo	**1**	**74**	**NA**	1.030 (-1.603, 3.663)
Green tea vs Placebo	**3**	**84**	(P=0.002), I^2=85.45	0.662 (-1.111, 2.434)
Curcumin vs Placebo	**2**	**57**	(P=0.462), I^2=0.00	0.269 (-0.595, 1.133)

Supplementary Figure S1.

Network plot of different nutrition supplements for overweight and obese treatment

IL-6

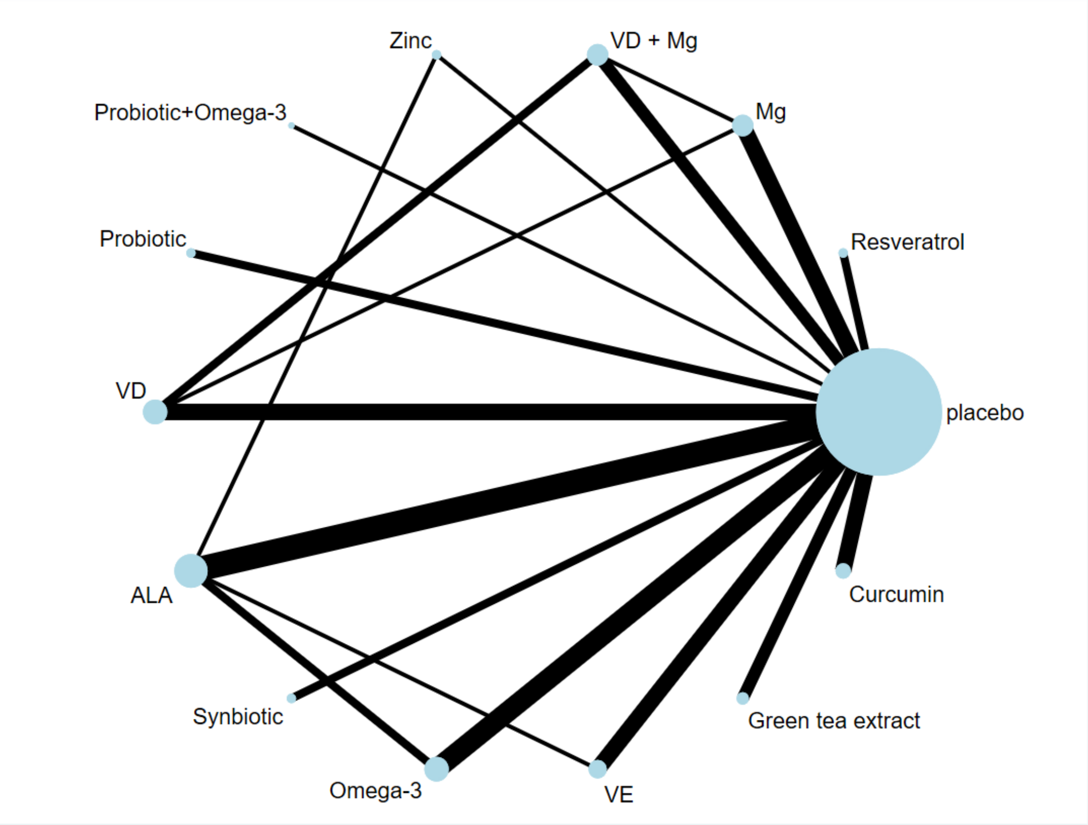

TNF-a

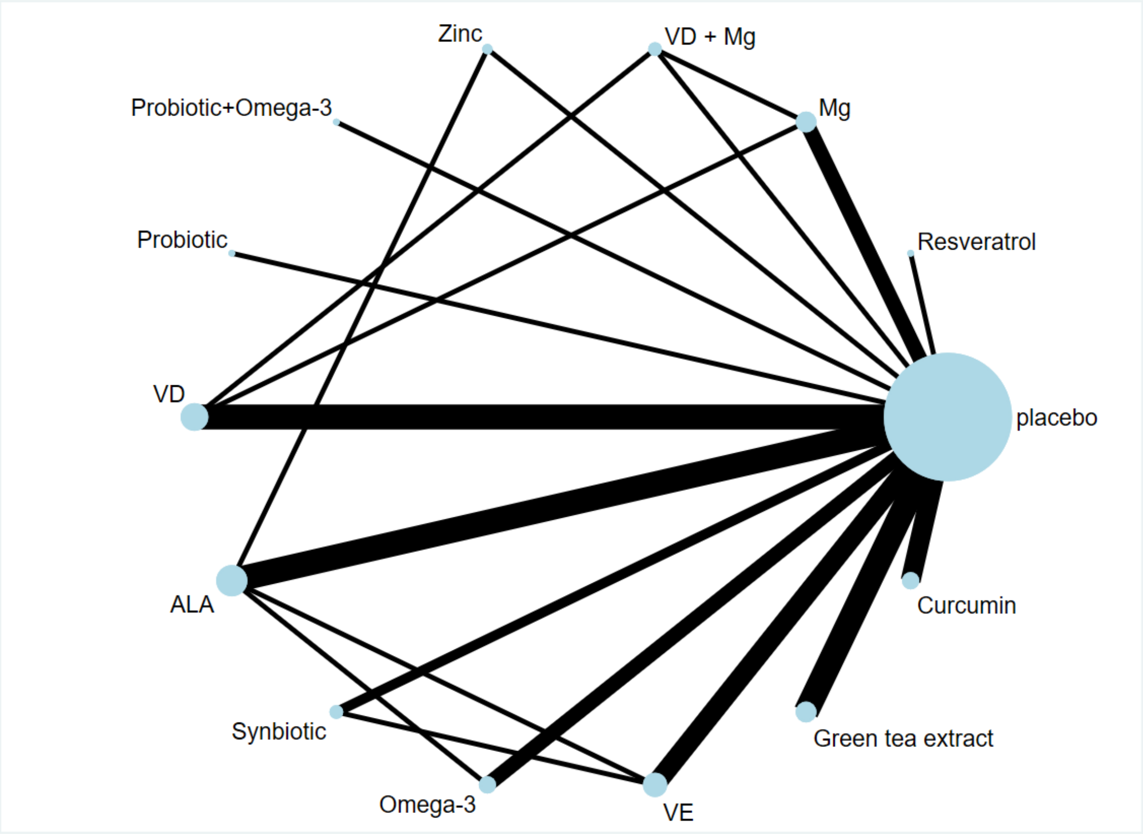

Leptin

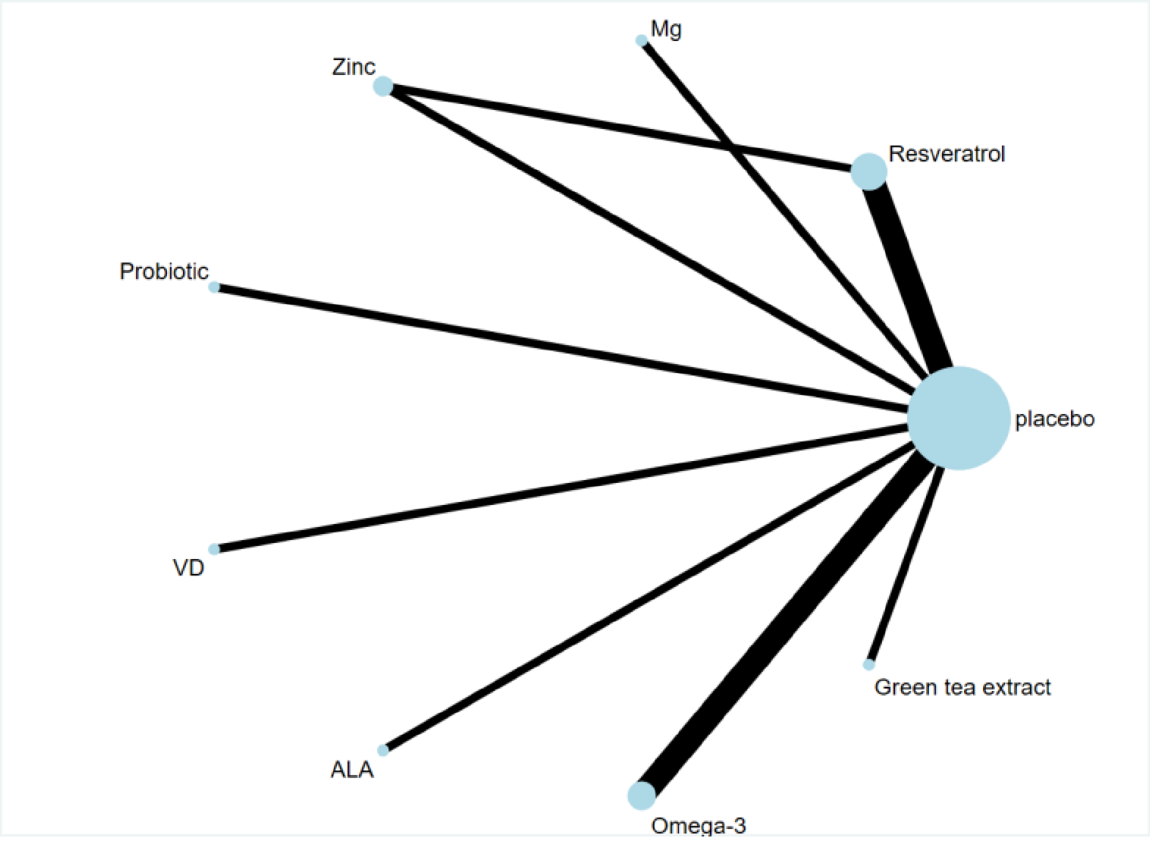

Adiponectin

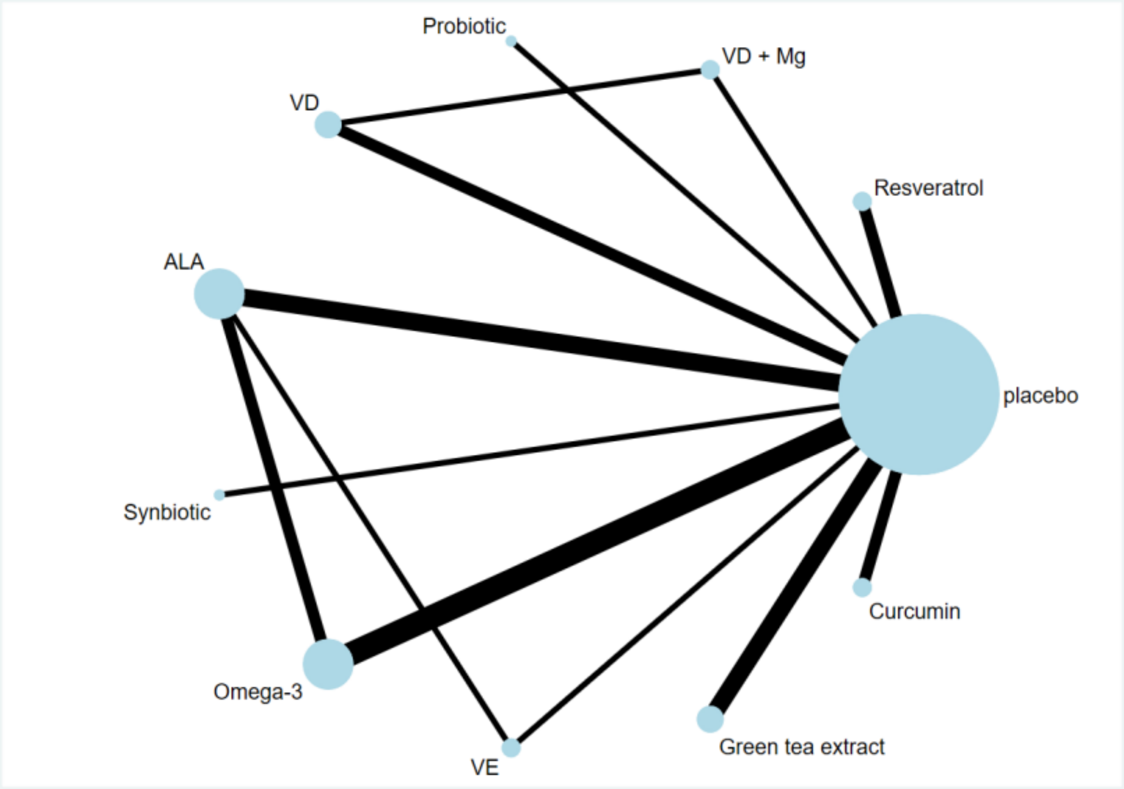

Supplementary Table S3

Inconsistency of IL-6，TNF-α，CRP, leptin, adiponectin, tested by loop-specific heterogeneity estimates, inconsistency model and node splitting analysis

Note:

loop-specific heterogeneity estimates

Loop	IF	seIF	z_value	p_value	CI_95	Loop_Heterog_tau2
		**IL-6**				
Placebo-Zinc-ALA	1.01	1.06	0.953	0.341	(0.00,3.09)	0.095
Placebo-VD + Mg-VD	0.755	0.601	1.255	0.209	(0.00,1.93)	0.073
Placebo-ALA-Omega-3	0.655	0.465	1.41	0.158	(0.00,1.57)	0.100
Mg-VD + Mg-VD	0.47	0.133	3.542	0.062	(0.00,0.73)	0.000
Placebo-Mg-VD	0.388	0.619	0.626	0.531	(0.00,1.60)	0.046
Placebo-Mg-VD + Mg	0.219	0.32	0.686	0.493	(0.00,0.85)	0.033
Placebo-ALA-VE	0.121	0.935	0.129	0.897	(0.00,1.95)	0.145
		**TNF-a**				
Placebo-Zinc-ALA	2.30	1.523	1.51	0.131	(0.00,5.29)	0.827
Placebo-ALA-VE	1.259	1.09	1.155	0.248	(0.00,3.40)	0.15
Placebo-Mg-VD	0.731	1.077	0.679	0.497	(0.00,2.84)	0.195
Placebo-Mg-VD + Mg	0.499	0.347	1.438	0.15	(0.00,1.18)	0.00
Placebo-ALA-Omega-3	0.496	1.461	0.34	0.734	(0.00,3.36)	0.795
Placebo-VD + Mg-VD	0.091	0.619	0.146	0.884	(0.00,1.30)	0.153
		**CRP**				
Placebo-Zinc-ALA	1.156	1.145	1.009	0.313	(0.00,3.40)	0.265
Placebo-Probiotic+Omega-3-Probiotic	1.054	0.531	1.986	0.057	(0.00,2.09)	0.009
Placebo-Probiotic-Synbiotic	0.932	1.145	0.814	0.416	(0.00,3.17)	0.279
Placebo-ALA-VE	0.681	1.156	0.589	0.556	(0.00,2.95)	0.269
Probiotic-ALA-Omega-3	0.601	0.333	1.805	0.071	(0.00,1.25)	0.00
Placebo-Probiotic-Omega-3	0.598	0.68	0.879	0.379	(0.00,1.93)	0.015
Placebo-Probiotic+ALA-ALA	0.473	1.701	0.278	0.781	(0.00,3.81)	0.579
Placebo-ALA-Omega-3	0.426	0.913	0.467	0.641	(0.00,2.22)	0.58
Placebo-VD + Mg-VD	0.414	0.568	0.729	0.466	(0.00,1.53)	0.183
Placebo-Probiotic-ALA	0.271	0.848	0.32	0.749	(0.00,1.93)	0.154
Placebo-Mg-VD + Mg	0.271	1.831	0.148	0.882	(0.00,3.86)	0.336
Placebo-Probiotic+Omega-3-Omega-3	0.225	0.801	0.281	0.779	(0.00,1.79)	0.15
Mg-VD + Mg-VD	0.13	0.051	2.551	0.051	(0.00,0.23)	0.00
Placebo-Probiotic+ALA-Probiotic	0.129	0.585	0.221	0.825	(0.00,1.28)	0.038
Placebo-Mg-VD	0.058	0.71	0.082	0.935	(0.00,1.45)	0.021
		**Leptin**				
Placebo-Resveratrol-VD + Mg	5.645	8.506	0.664	0.507	(0.00,22.32)	0.00

Adiponectin						
Placebo-ALA-VE	1.039	0.473	2.196	0.058	(0.00,1.97)	0.00
Placebo-ALA-Omega-3	0.735	3.048	0.241	0.809	(0.00,6.71)	2.826
Placebo-VD + Mg-VD	0.19	0.227	0.837	0.403	(0.00,0.63)	0.00

Inconsistency model

	IL-6	TNF-a	CRP	Leptin	Adiponectin
chi2	1.21	4.23	8.92	1.10	7.56
Prob > chi2	0.999	0.646	0.962	0.293	0.182

Node splitting analysis

Side	Direct Coef.	Std. Err.	Indirect Coef.	Std. Err.	Difference Coef.	Std. Err.	P>z
**IL-6**							
A C	0.169	0.894	-0.620	3.067	0.789	3.195	0.805
A D	-0.263	1.032	-1.043	2.623	0.780	2.819	0.782
A E	-0.640	1.789	-3.012	3.431	2.372	3.861	0.539
A H	-0.423	0.895	0.541	2.887	-0.964	3.023	0.750
A I	-0.625	0.757	-1.015	1.691	0.390	1.852	0.833
A K	-0.399	0.803	-0.132	1.841	-0.268	2.009	0.894
A L	-0.853	0.915	-1.302	3.459	0.448	3.578	0.900
C D	-0.200	1.787	-0.691	1.581	0.491	2.386	0.837
C H	-0.080	1.786	-0.682	1.436	0.602	2.292	0.793
D H	0.355	1.259	-0.890	2.118	1.245	2.464	0.613
E I	0.960	1.789	-1.412	3.430	2.372	3.861	0.539
I K	0.510	1.264	0.171	1.213	0.339	1.752	0.846
I L	0.031	1.789	-0.311	1.300	0.342	2.212	0.877
**TNF-α**							
A C	0.588	0.767	-0.178	2.610	0.766	2.710	0.777
A D	-0.710	1.292	0.611	2.147	-1.321	2.506	0.598
A E	0.900	1.283	-3.340	2.542	4.240	2.852	0.137
A C	0.588	0.767	-0.178	2.610	0.766	2.710	0.777
A H	-0.250	0.567	2.637	2.925	-2.886	2.979	0.333
A I	-0.786	0.584	-0.175	1.388	-0.611	1.506	0.685
A K	-0.175	0.921	-0.592	1.414	0.416	1.687	0.805
A L	-0.031	0.746	-1.798	2.518	1.767	2.625	0.501
C D	-0.310	1.302	-3.196	2.649	2.886	2.979	0.333
C H	0.059	1.315	-1.199	1.114	1.259	1.720	0.464
D H	0.390	1.298	-0.376	2.379	0.766	2.710	0.777
E I	0.200	1.308	-4.040	2.504	4.240	2.852	0.137
I K	0.150	1.292	0.567	1.085	-0.417	1.687	0.805
I L	-0.100	1.280	0.964	1.081	-1.064	1.675	0.525
**CRP**							
A C	-0.600	0.434	-0.249	1.589	-0.351	1.647	0.831

A D	-0.546	0.534	-0.832	1.346	0.286	1.448	0.843
A E	-0.888	0.682	-3.441	1.788	2.553	1.913	0.182
A F	-0.290	0.922	-1.127	1.428	0.837	1.700	0.623
A G	-2.010	0.947	-1.122	1.491	-0.888	1.772	0.616
A H	-0.400	0.407	-1.832	1.055	1.433	1.131	0.205
A I	-0.013	0.454	-1.850	1.446	1.837	1.516	0.226
A J	-0.515	0.376	-0.066	0.851	-0.449	0.931	0.629
A K	-1.227	0.425	-0.682	1.826	-0.544	1.875	0.772
A L	-0.260	0.414	-0.526	0.979	0.267	1.061	0.802
A M	-0.216	0.514	-1.072	1.796	0.856	1.867	0.647
C D	-0.260	0.925	0.172	0.789	-0.432	1.216	0.722
C I	-0.060	0.922	0.667	0.712	-0.727	1.165	0.532
D I	0.135	0.646	1.177	1.090	-1.043	1.267	0.411
E J	1.147	0.952	0.360	1.005	0.787	1.396	0.573
F H	0.090	0.923	-0.475	1.609	0.565	1.855	0.761
F J	0.200	0.924	-0.208	1.557	0.408	1.811	0.822
G H	0.740	0.942	2.450	1.638	-1.710	1.895	0.367
G L	1.640	0.974	0.938	1.649	0.702	1.943	0.718
H J	0.110	0.925	0.156	0.573	-0.046	1.088	0.966
H K	0.171	0.906	-1.001	0.638	1.171	1.108	0.291
H L	0.912	0.973	0.052	0.595	0.859	1.141	0.451
J L	-0.145	0.745	0.322	0.591	-0.467	0.952	0.624
J M	-0.566	0.942	0.556	0.699	-1.122	1.171	0.338
				**Leptin**			
A D	16.100	7.125	29.379	11.793	-13.279	12.823	0.300
B D	20.050	6.740	6.771	12.453	13.279	12.823	0.300
				**Adiponectin**			
A C	-0.040	1.525	0.340	4.059	-0.380	4.337	0.930
A F	0.450	0.916	-0.895	3.466	1.346	3.582	0.707
A H	0.367	0.778	5.234	3.379	-4.867	3.476	0.162
A I	-0.040	1.534	0.909	3.371	-0.949	3.702	0.798
C E	0.050	1.525	0.430	4.060	-0.380	4.337	0.930
F H	0.848	1.569	-0.209	1.435	1.057	2.133	0.620
F I	-0.070	1.545	-1.019	3.357	0.949	3.702	0.798

Supplementary Table S4

Risk of bias table

StudyID	Randomization process	Deviations from intended interventions	Mising outcome data	Measurement of the outcome	Selection of the reported result	Overall Bias
Kanazawa 2021	Low	Low	Low	Low	Low	Low
Huerta 2015	Low	Low	Low	Low	Low	Low
Cunningham 2026	Low	Low	Low	Some concerns	Low	Some concerns
Basu 2010	Low	High	Low	Low	Low	High
Arzola 2016	Low	Low	Low	Low	Low	Low
Abiri 2021	Low	Low	Some concerns	Low	Low	Some concerns
de Oliveira Vogado 2024	Low	Low	Low	Low	Low	Low
Duggan 2015	Low	Low	Low	Low	Low	Low
Laue 2023	Low	Low	Some concerns	Low	Low	Some concerns
Devaraj 2008	Low	Low	Low	Low	Low	Low
Farrokhian 2017	Low	Low	Some concerns	Low	Low	Some concerns
Kheyruri 2020	Low	Low	Low	Low	Low	Low
Foster 2013	Low	Low	Low	Low	Low	Low
Ganjali 2014	Low	Some concerns	Low	Low	Low	Some concerns
Ekhlasi 2017	Low	Low	Low	Low	Low	Low
Hejazi 2015	Low	Low	Low	Low	Low	Low
Hellmann 2022	Low	Low	Low	Low	Low	Low
Rajkumar 2014	Low	Low	Some concerns	Some concerns	Low	Some concerns
Huerta 2015	Low	Low	Low	Low	Low	Low
Munro 2013	Low	Low	Some concerns	Low	Low	Some concerns
Joris 2017	Low	Low	Low	Low	Low	Low
Kantartzis 2018	Low	Low	Low	Low	Low	Low
Khodadoust 2021	Low	Low	Some concerns	Low	Low	Some concerns
Khorsandi 2019	Low	Low	Low	High	Low	High
Krebs 2006	Low	Low	Some concerns	Low	Low	Some concerns
Nogueira 2016	Low	Low	Low	Low	Low	Low
Lyon 2023	Low	Low	Low	Low	Low	Low
Raji Lahiji 2021	Low	Low	Low	Some concerns	Low	Some concerns
Manning 2012	Low	Low	Low	Low	Low	Low
Marfella 2015	Low	Low	Low	Low	Low	Low
Campbell 2019	Low	Low	Low	Low	Low	Low

Cheung 2022	Low	Low	Low	Low	Low	Low
Mohammadi 2015	Low	Low	Low	Low	Low	Low
Moslehi 2012	Low	Low	Low	Low	Low	Low
Nasiri 2021	Low	Low	Low	Low	Low	Low
Bakker 2023	Low	Low	Low	Low	Low	Low
Kobyliak 2020	Low	Low	Low	Low	Low	Low
Noori 2025	Low	Some concerns	Low	Low	Low	Some concerns
Pauls 2021	Low	Low	Low	Low	Low	Low
Bogdanski 2012	Low	Low	Low	Low	Low	Low
Pervez 2018	Low	Low	Low	Low	Low	Low
Santellano 2024	Some concerns	Low	Low	Some concerns	Low	Some concerns
Poulsen 2012	Low	Low	Low	Low	Low	Low
Bagheri 2020	Low	Low	Some concerns	Low	Low	Some concerns
Rabiei 2018	Low	Low	Some concerns	Some concerns	Low	Some concerns
Sohaei 2019	Low	Low	Low	Low	Low	Low
Chacko 2010	Low	Low	Low	Low	Low	Low
Sardu 2017	Low	Low	Low	Some concerns	Low	Some concerns
Jazayeri-Tehrani 2019	Low	Low	Some concerns	Low	Low	Some concerns
Seyyedebrahimi 2018	Low	Low	Low	Low	Low	Low
Stonehouse 2016	Low	Low	Low	Low	Low	Low
Agbalalah 2022	Low	Low	Low	Low	Low	Low
Tenorio-Jiménez 2019	Low	Low	Low	Low	Low	Low
Walker 2018	Low	Low	Low	Some concerns	Low	Some concerns
Yaikwawong 2024	Low	Low	Low	Low	Low	Low
Zhang 2011	Low	Some concerns	Low	Low	Low	Some concerns
Zittermann 2009	Low	Low	Low	Low	Low	Low
Khosla 2013	Low	Low	Low	Low	Low	Low
Talari 2019	Low	Low	Low	Low	Low	Low
Itariu 2012	Low	High	Low	Low	Low	High

Supplementary Figure S2.
Risk of bias graph

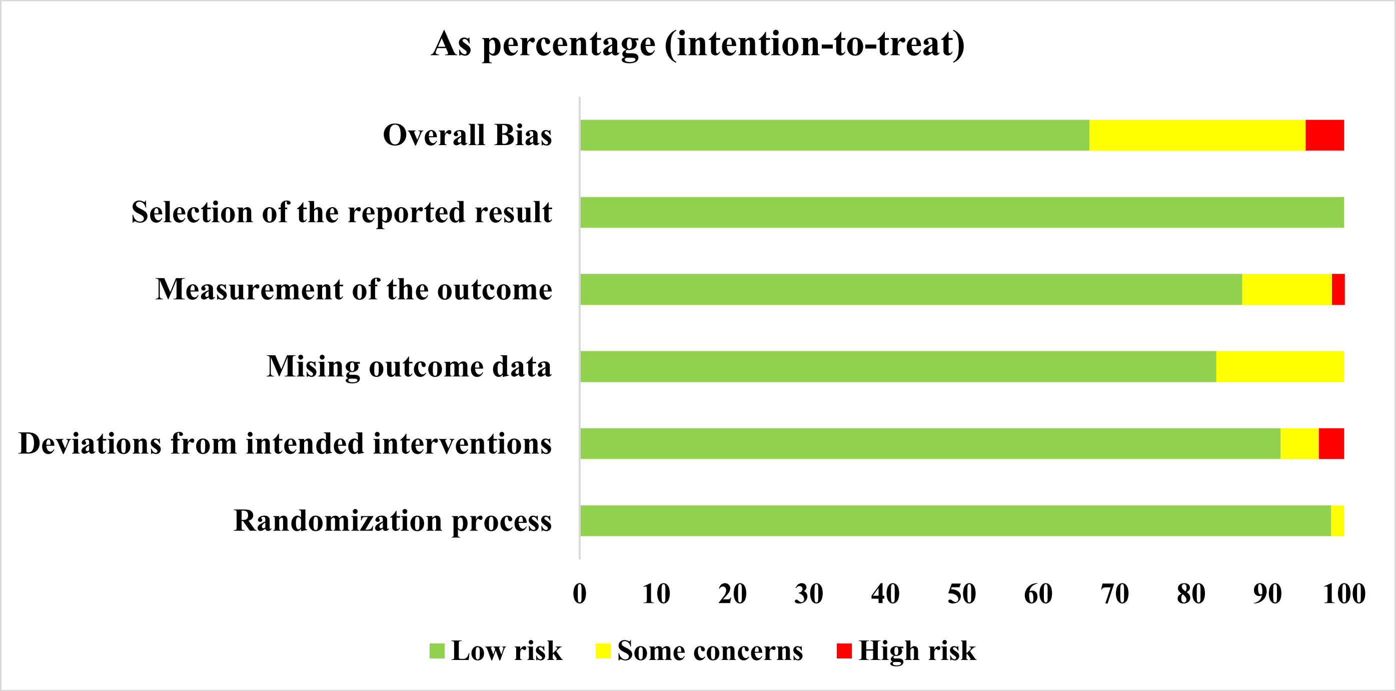

Unique ID	D1	D2	D3	D4	D5	Overall
Kanazawa 2021	+	+	+	+	+	+
Huerta 2015	+	+	+	+	+	+
Cunningham 2026	+	+	+	!	+	!
Basu 2010	+	-	+	+	+	-
Arzola-Paniagua 2016	+	+	+	+	+	+
Abiri 2021	+	+	!	+	+	!
de Oliveira Vogado 2024	+	+	+	+	+	+
Duggan 2015	+	+	+	+	+	+
Laue 2023	+	+	!	+	+	!
Devaraj 2008	+	+	+	+	+	+
Farrokhian 2017	+	+	!	+	+	!
Kheyruri 2020	+	+	+	+	+	+
Foster 2013	+	+	+	+	+	+
Ganjali 2014	+	!	+	+	+	!
Ekhlasi 2017	+	+	+	+	+	+
Hejazi 2015	+	+	+	+	+	+
Hellmann 2022	+	+	+	+	+	+
Rajkumar 2014	+	+	!	!	+	!
Huerta 2015	+	+	+	+	+	+
Munro 2013	+	+	!	+	+	!
Joris 2017	+	+	+	+	+	+
Kantartzis 2018	+	+	+	+	+	+
Khodadoust 2021	+	+	!	+	+	!
Khorsandi 2019	+	+	+	-	+	-
Krebs 2006	+	+	!	+	+	!
Nogueira 2016	+	+	+	+	+	+
Lyon 2023	+	+	+	+	+	+
Raji Lahiji 2021	+	+	+	!	+	!
Manning 2012	+	+	+	+	+	+
Marfella 2015	+	+	+	+	+	+
Campbell 2019	+	+	+	+	+	+
Cheung 2022	+	+	+	+	+	+
Mohammadi 2015	+	+	+	+	+	+
Moslehi 2012	+	+	+	+	+	+
Nasiri 2021	+	+	+	+	+	+
Bakker 2023	+	+	+	+	+	+

+ Low risk
! Some concerns
- High risk

D1 Randomisation process
D2 Deviations from the intended interventions
D3 Missing outcome data
D4 Measurement of the outcome
D5 Selection of the reported result

Unique ID	D1	D2	D3	D4	D5	Overall
Kanazawa 2021	+	+	+	+	+	+
Huerta 2015	+	+	+	+	+	+
Cunningham 2026	+	+	+	!	+	!
Basu 2010	+	-	+	+	+	-
Arzola-Paniagua 2016	+	+	+	+	+	+
Abiri 2021	+	+	!	+	+	!
de Oliveira Vogado 2024	+	+	+	+	+	+
Duggan 2015	+	+	+	+	+	+
Laue 2023	+	+	!	+	+	!
Devaraj 2008	+	+	+	+	+	+
Farrokhian 2017	+	+	!	+	+	!
Kheyruri 2020	+	+	+	+	+	+
Foster 2013	+	+	+	+	+	+
Ganjali 2014	+	!	+	+	+	!
Ekhlasi 2017	+	+	+	+	+	+
Hejazi 2015	+	+	+	+	+	+
Hellmann 2022	+	+	+	+	+	+
Rajkumar 2014	+	+	!	!	+	!
Huerta 2015	+	+	+	+	+	+
Munro 2013	+	+	!	+	+	!
Joris 2017	+	+	+	+	+	+
Kantartzis 2018	+	+	+	+	+	+
Khodadoust 2021	+	+	!	+	+	!
Khorsandi 2019	+	+	+	-	+	-
Krebs 2006	+	+	!	+	+	!
Nogueira 2016	+	+	+	+	+	+
Lyon 2023	+	+	+	+	+	+
Raji Lahiji 2021	+	+	+	!	+	!
Manning 2012	+	+	+	+	+	+
Marfella 2015	+	+	+	+	+	+
Campbell 2019	+	+	+	+	+	+
Cheung 2022	+	+	+	+	+	+
Mohammadi 2015	+	+	+	+	+	+
Moslehi 2012	+	+	+	+	+	+
Nasiri 2021	+	+	+	+	+	+
Bakker 2023	+	+	+	+	+	+

+ Low risk
! Some conc
- High risk

D1 Randomisa
D2 Deviations
D3 Missing ou
D4 Measurem
D5 Selection o

Supplementary Figure S3.

The funnel plot graphics of IL-6, TNF-α,CRP, leptin, and adiponectin in NMA

IL-6

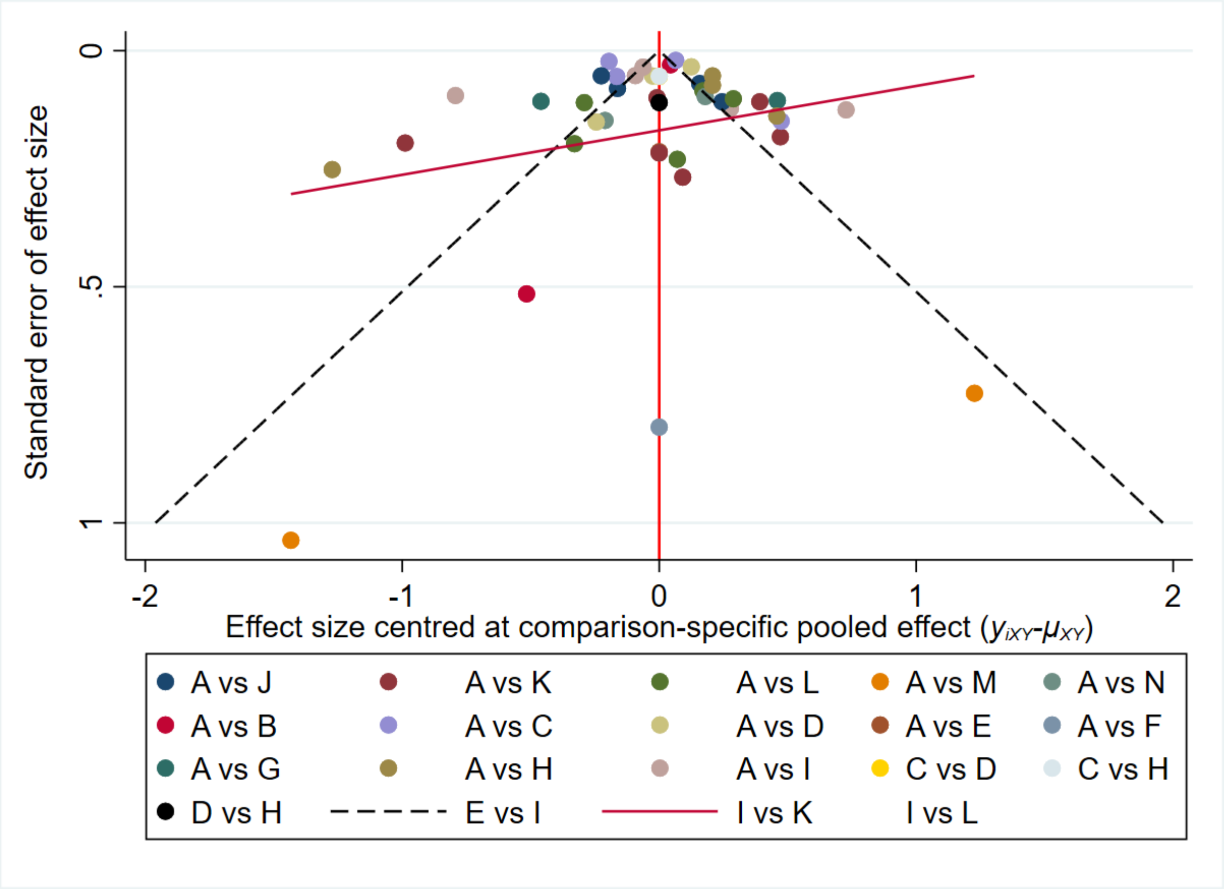

TNF-α

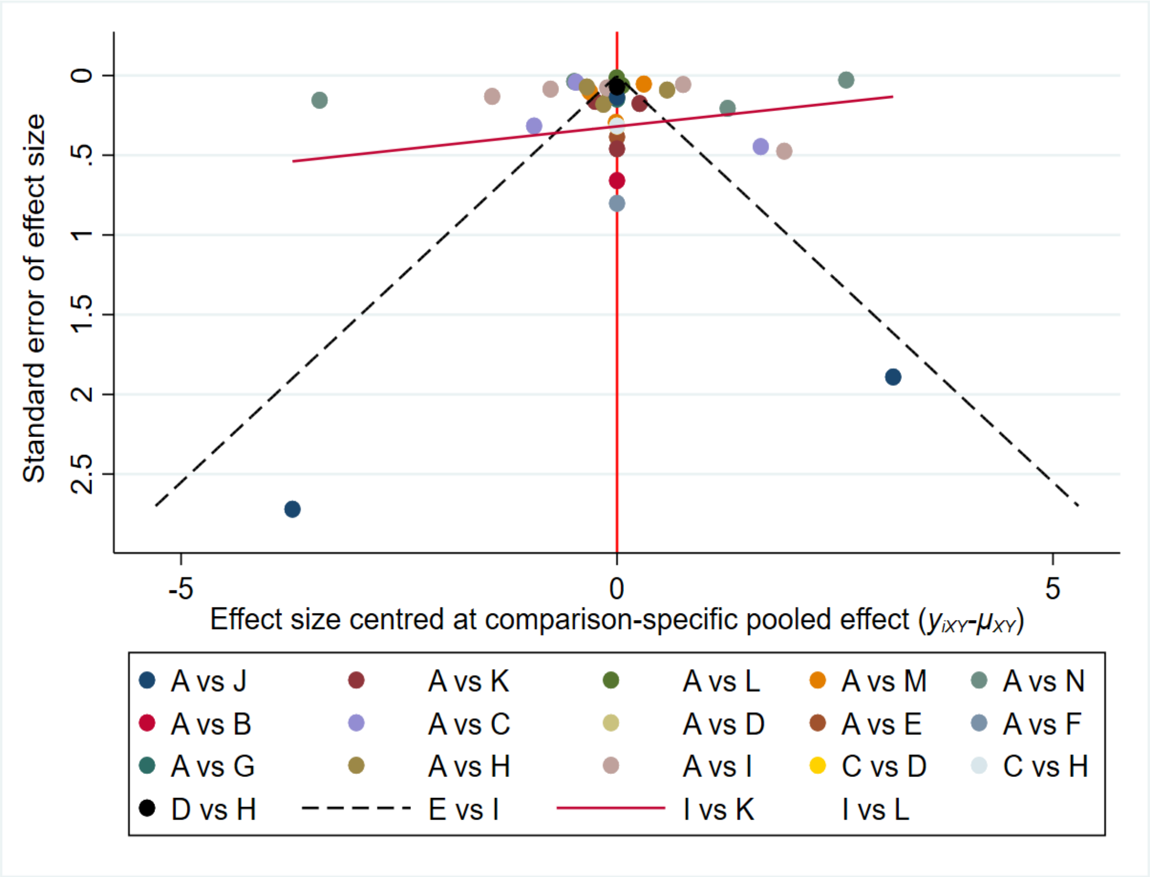

Standard error of effect size
0
.5
1
1.5
2
2.5
-5
0
5
Effect size centred at comparison-specific pooled effect (y_{iXY}-μ_{XY})
A vs J
A vs K
A vs L
A vs M
A vs N
A vs B
A vs C
A vs D
A vs E
A vs F
A vs G
A vs H
A vs I
C vs D
C vs H
D vs H
E vs I
I vs K
I vs L

CRP

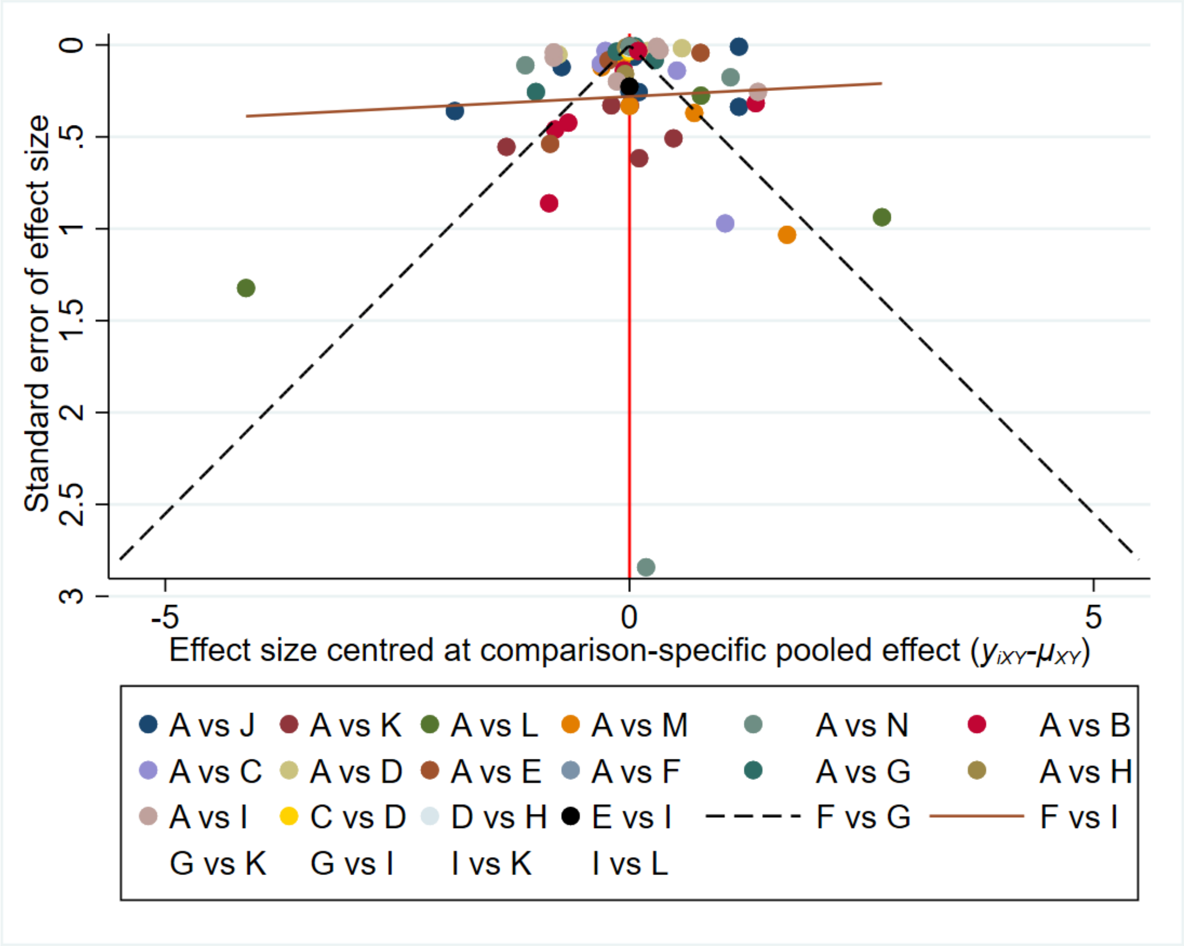

Standard error of effect size
0
.5
1
1.5
2
2.5
3
-5
0
5
Effect size centred at comparison-specific pooled effect (y_{iXY}-μ_{XY})
A vs J
A vs K
A vs L
A vs M
A vs N
A vs B
A vs C
A vs D
A vs E
A vs F
A vs G
A vs H
A vs I
C vs D
D vs H
E vs I
F vs G
F vs I
G vs K
G vs I
I vs K
I vs L

Leptin

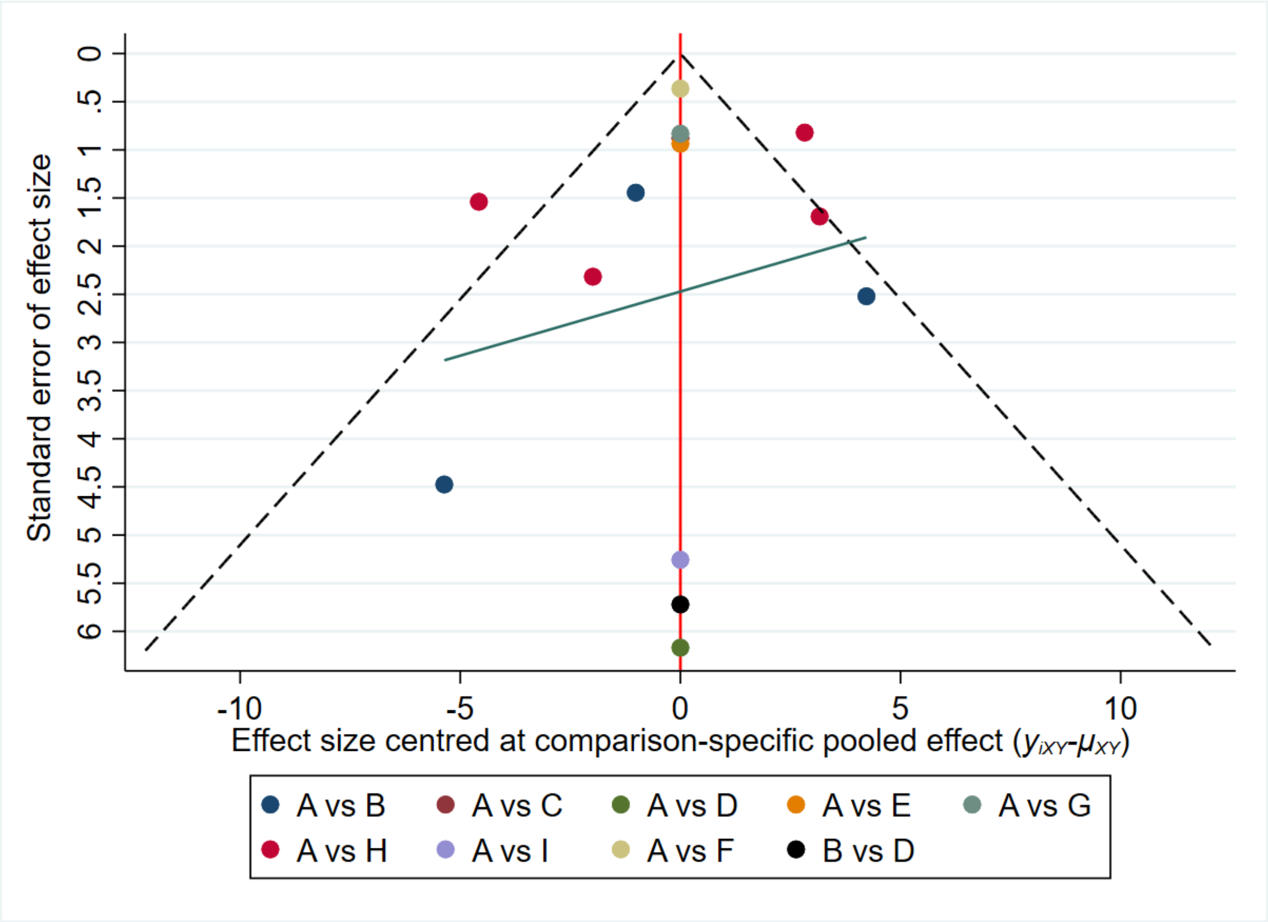

Adiponectin

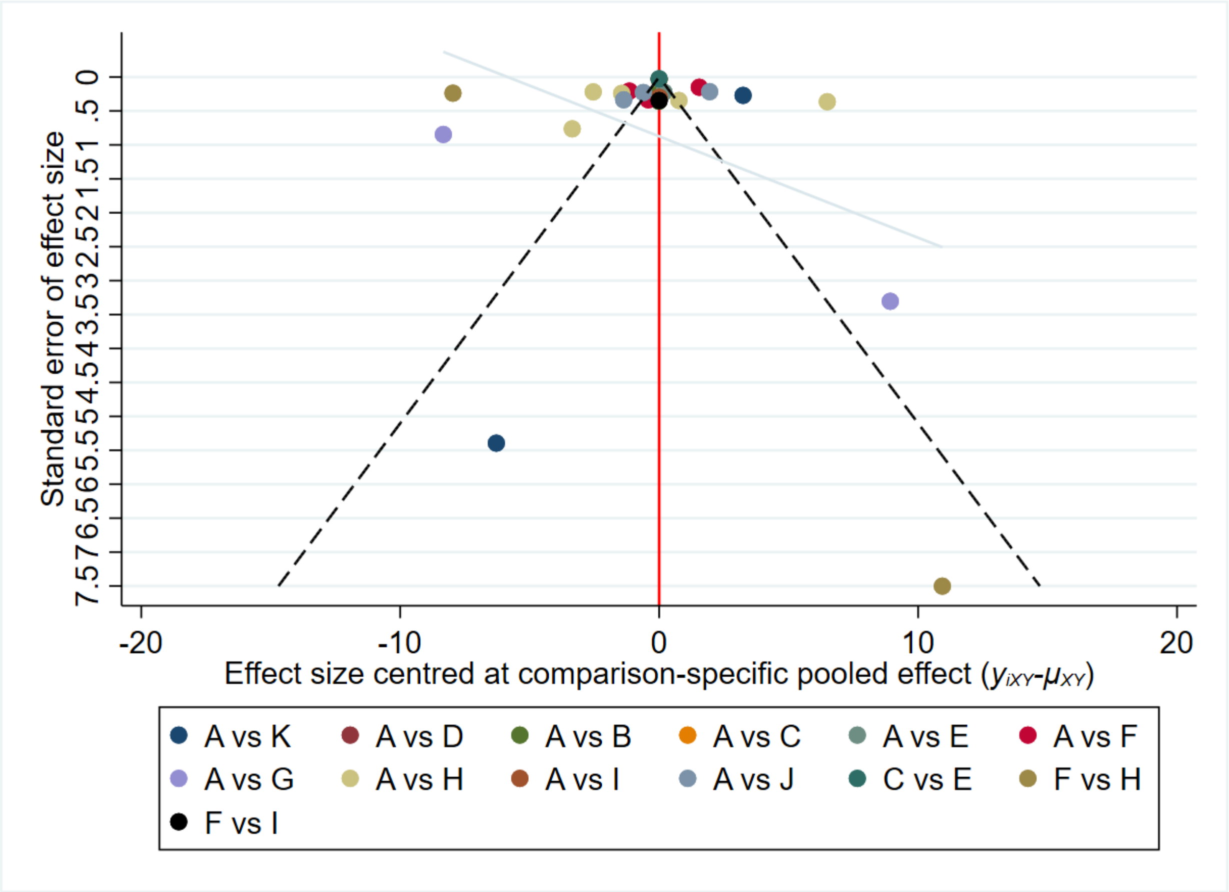

Supplementary Table S5

Results of Meta-regression.

IL-6									
	**Study (n)**	**Coef.**	**SE**	**Z**	**P**	**95% CI**	**R^2 (%)**	**Tau^2**	**I^2 (%)**
**Period**	36	-0.002	0.006	-0.33	0.738	[-0.013,0.009]	0.00	0.1195	70.74
**Age**	35	0.009	0.0065	1.4	0.16	[-0.004,0.022]	8.05	0.0811	63.2
**Exercise**	36	-0.578	0.198	-2.92	0.003	[-0.966, -0.198]	21.86	0.899	62.00
**Diet control**	36	-0.367	0.142	-2.58	0.01	[-0.646,-0.088]	21.58	0.090	64.27
**BMI**	35	-0.039	0.0215	-1.82	0.069	[-0.081, 0.003]	13.92	0.102	68.48
**Male proportion**	34	0.000	0.002	0.11	0.912	[-0.004,0.005]	0.00	0.094	66.70
**TNF-α**									
	**Study (n)**	**Coef.**	**SE**	**Z**	**P**	**95% CI**	**R^2 (%)**	**Tau^2**	**I^2 (%)**
**Period**	29	0.004	0.009	0.460	0.642	[-0.014, 0.023]	0.00	0.388	83.08
**Age**	28	0.036	0.014	2.650	0.008	[0.009, 0.063]	37.47	0.235	75.30
**Exercise**	29	0.227	0.618	0.370	0.713	[-0.985, 1.439]	0.00	0.40	85.36
**Diet control**	29	-0.145	0.318	-0.460	0.649	[-0.768, 0.478]	0.00	0.405	80.43
**BMI**	28	-0.100	0.054	-1.840	0.066	[-0.207, 0.007]	11.67	0.358	79.32
**Male proportion**	27	0.001	0.006	0.100	0.921	[-0.010, 0.012]	0.00	0.437	84.51
**CRP**									
	**Study (n)**	**Coef.**	**SE**	**Z**	**P**	**95% CI**	**R^2 (%)**	**Tau^2**	**I^2 (%)**
**Period**	44	-0.008	0.005	-1.72	0.085	[-0.018, 0.001]	7.97	0.113	92.69
**Age**	42	-0.004	0.006	-0.65	0.517	[-0.016, 0.008]	0.00	0.130	93.77
**Exercise**	44	-0.484	0.212	-2.28	0.022	[-0.900, -0.068]	18.29	0.100	92.27
**Diet control**	44	0.014	0.144	0.1	0.924	[-0.268, 0.295]	0.00	0.128	93.57
**BMI**	43	0.044	0.023	1.92	0.055	[-0.001, 0.089]	6.42	0.121	92.79
**Male proportion**	41	-0.001	0.003	-0.33	0.74	[-0.006, 0.004]	0.00	0.163	86.71
**Adiponectin**									
	**Study (n)**	**Coef.**	**SE**	**Z**	**P**	**95% CI**	**R^2 (%)**	**Tau^2**	**I^2 (%)**
**Period**	18	-0.001	0.024	-0.02	0.982	[-0.047, 0.046]	0.00	0.883	86.66
**Age**	17	0.076	0.034	2.26	0.024	[0.010, 0.141]	33.75	0.566	80.86
**Exercise**	18	1.816	0.740	2.45	0.014	[0.365, 3.267]	31.57	0.577	80.97
**Diet control**	18	0.473	0.655	0.72	0.470	[-0.811, 1.758]	0.00	0.916	86.79
**BMI**	16	-0.128	0.085	-1.5	0.133	[-0.294, 0.039]	16.87	0.770	85.60
**Male proportion**	16	0.024	0.008	3.13	0.002	[0.009, 0.040]	63.91	0.343	72.19

Supplementary Figure S4.

Results of network meta-regression analysis

IL-6

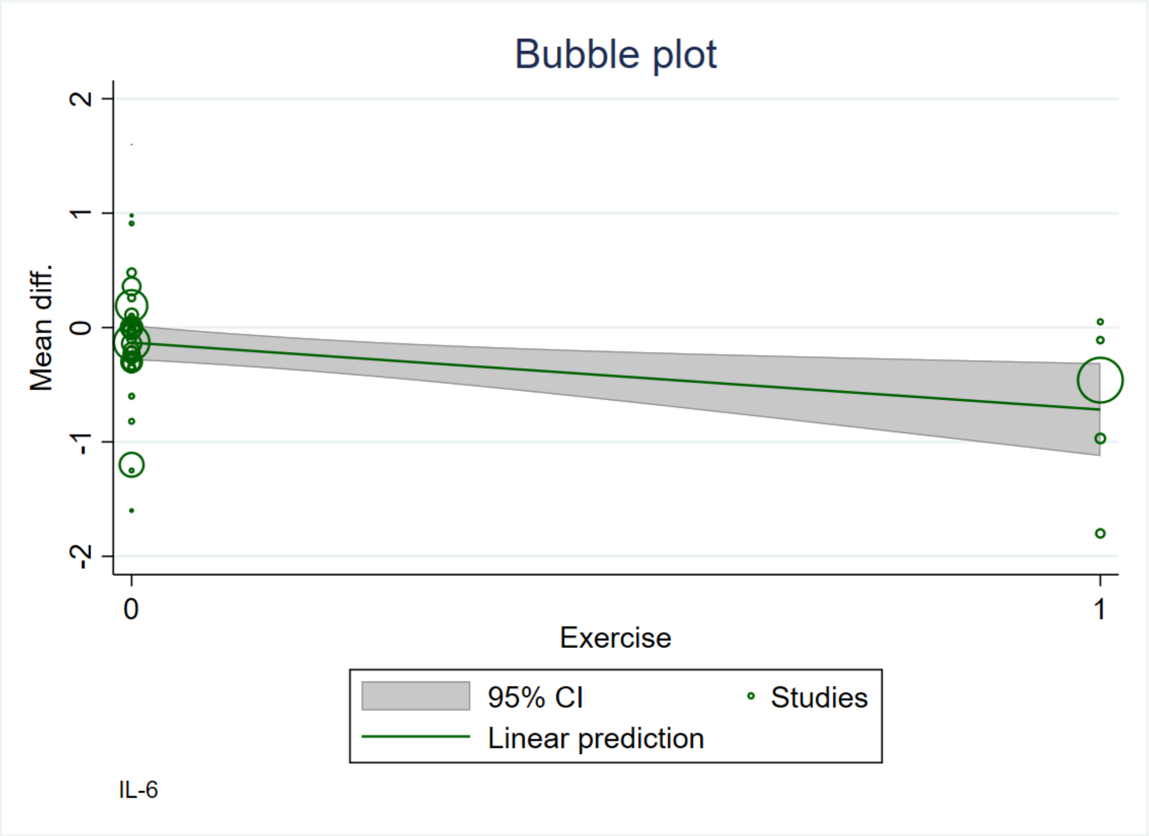

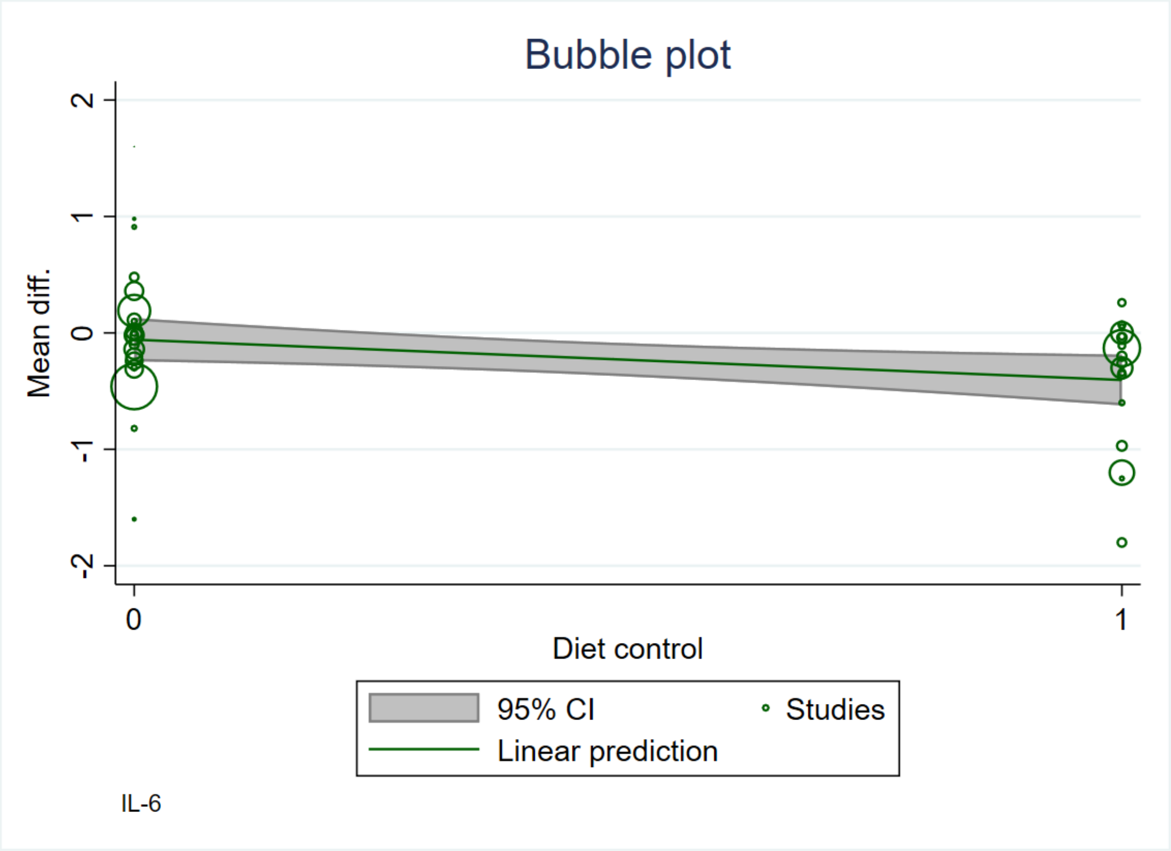

TNF-α

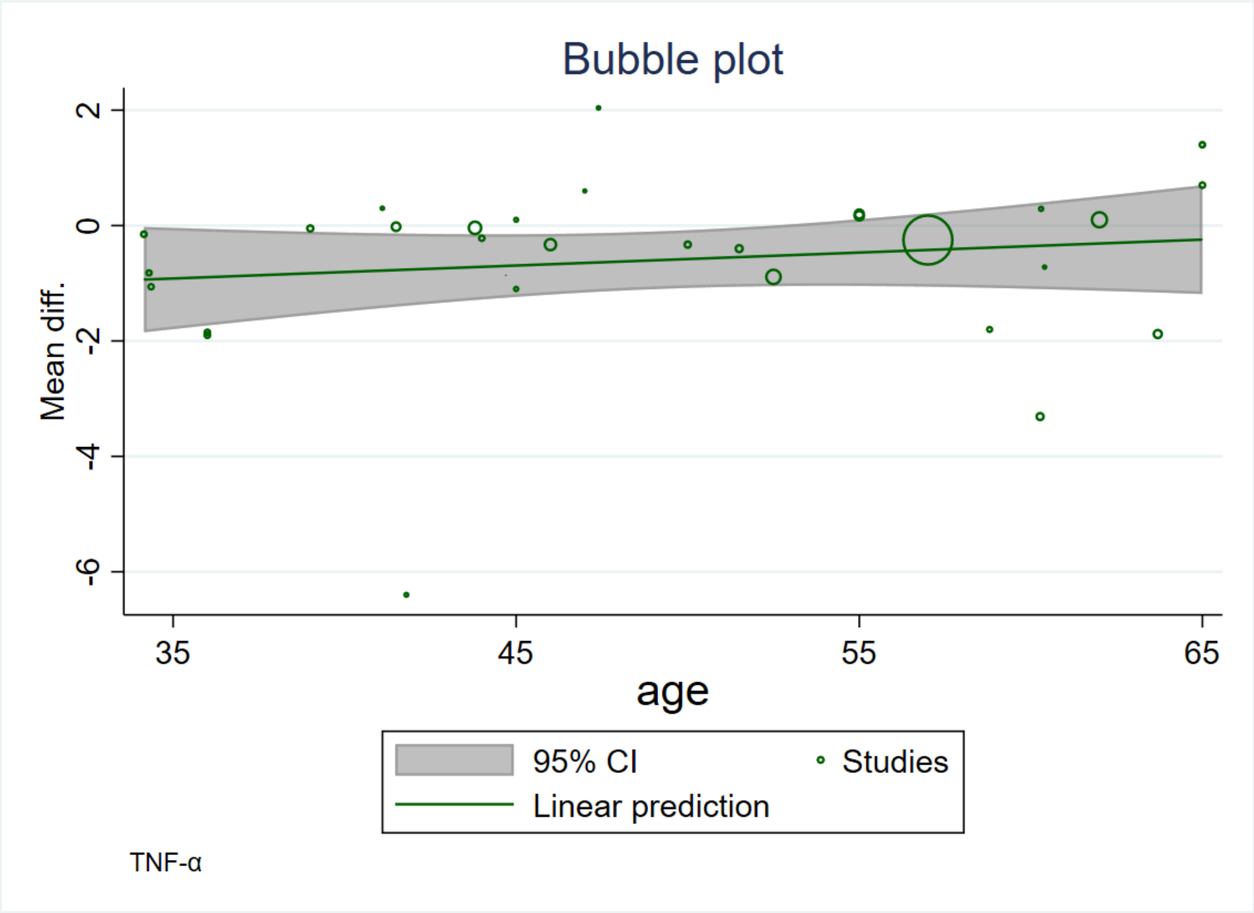

Bubble plot
2
0
-2
-4
-6
Mean diff.
35
45
55
65
age
95% CI
Studies
Linear prediction
TNF-α

CRP

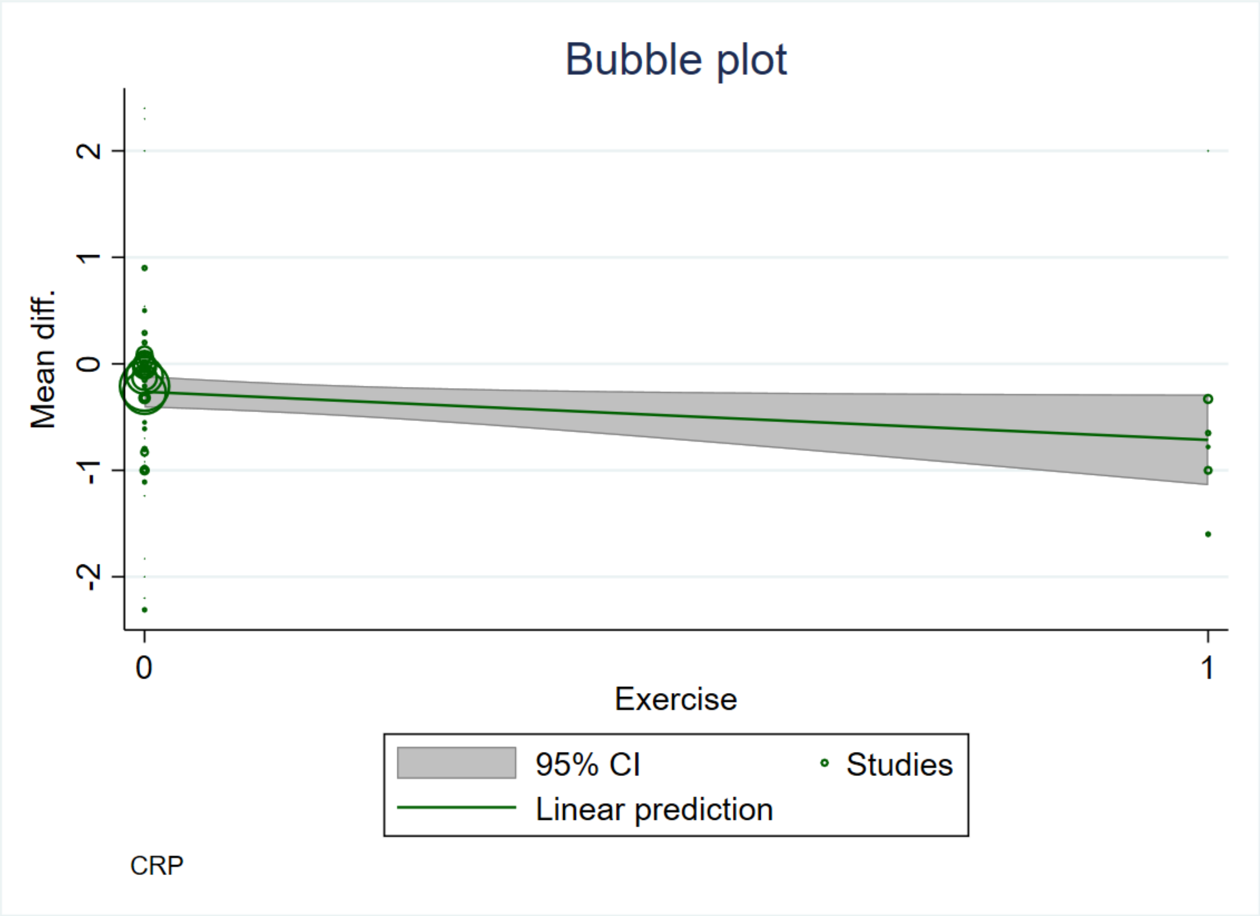

Bubble plot
2
1
0
-1
-2
Mean diff.
0
1
Exercise
95% CI
Studies
Linear prediction
CRP

Adiponectin

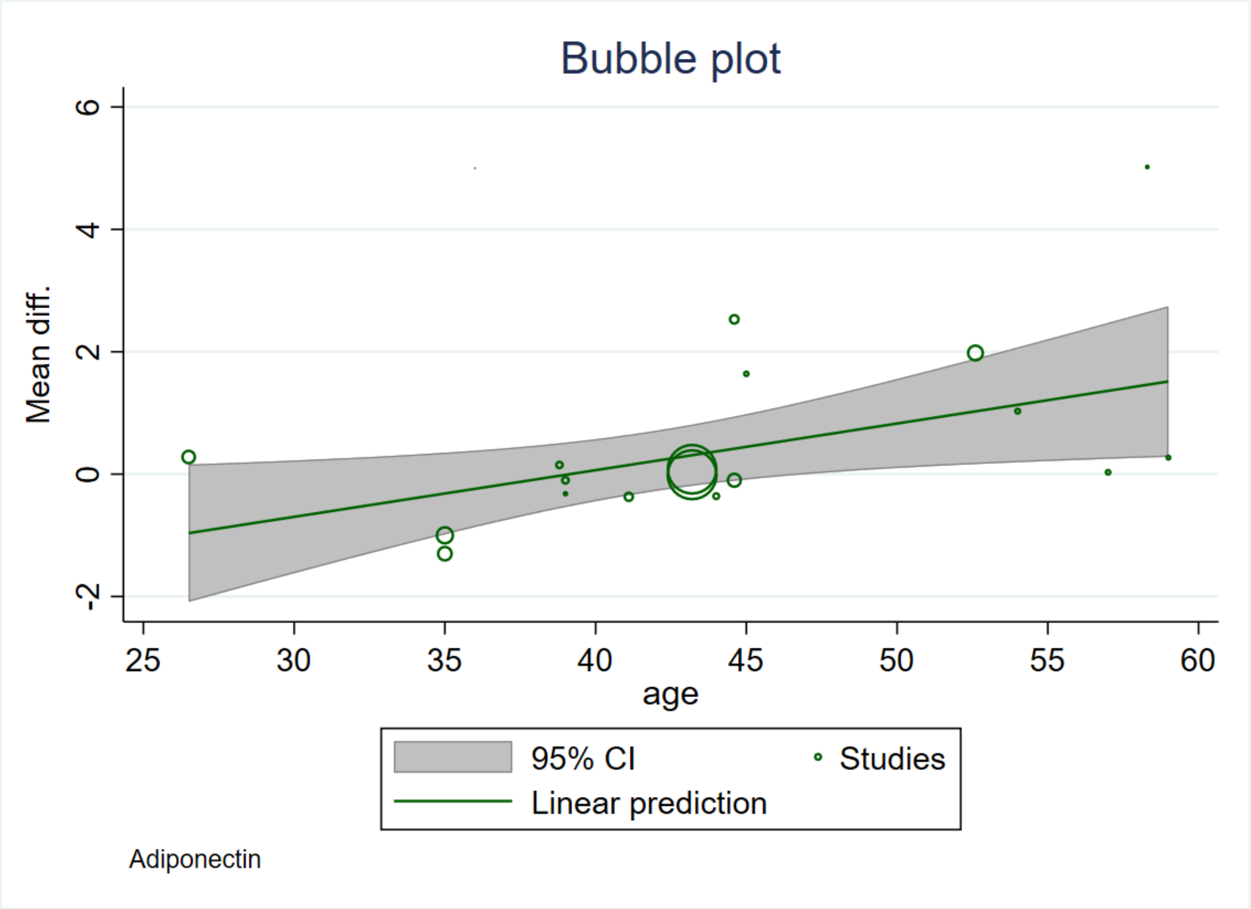

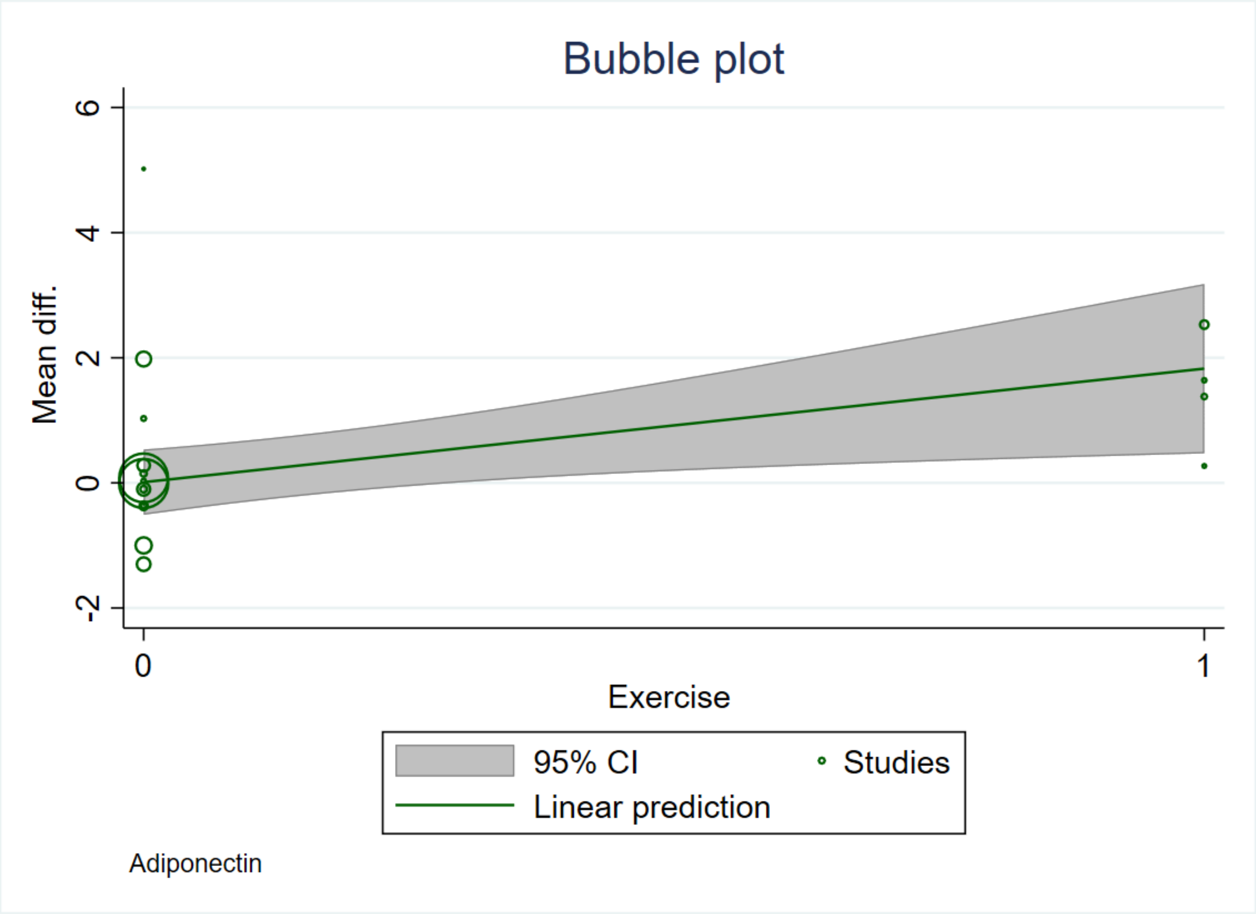

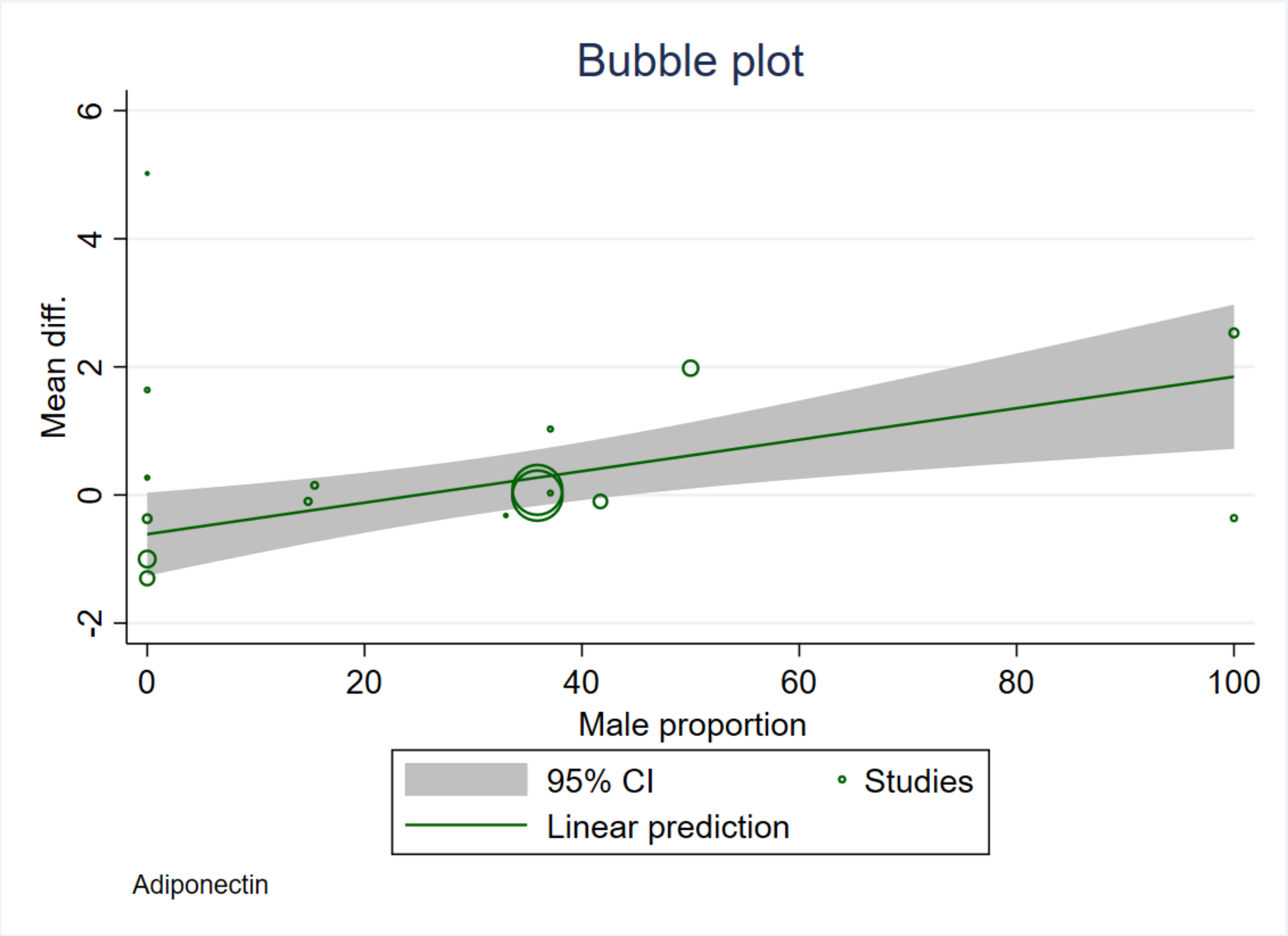
Bubble plot
Mean diff.
-2
0
2
4
6
0
20
40
60
80
100
Male proportion
95% CI
Studies
Linear prediction
Adiponectin

Supplementary Table S6.

CINeMA assessment of the confidence.

Comparison	Within-study bias	Reporting bias	Indirectness	Imprecision	Heterogeneity	Incoherence	Overall confidence
			**IL-6**				
Placebo vs Resveratrol	No concerns	Low risk	Some concerns	Major concerns	No concerns	No concerns	**Very low**
Placebo vs Mg	No concerns	Low risk	No concerns	Major concerns	No concerns	No concerns	**Low**
Placebo vs VD＋Mg	No concerns	Low risk	No concerns	Major concerns	No concerns	No concerns	**Low**
Placebo vs Zinc	No concerns	Low risk	Some concerns	Major concerns	No concerns	No concerns	**Very low**
Placebo vs omega-3+Probiotics	No concerns	Low risk	Some concerns	Some concerns	Some concerns	No concerns	**Low**
Placebo vs Probiotics	No concerns	Low risk	No concerns	Major concerns	No concerns	No concerns	**Low**
Placebo vs VD	No concerns	Low risk	No concerns	Major concerns	No concerns	No concerns	**Low**
Placebo vs α-lipoic acid	No concerns	Low risk	No concerns	No concerns	Major concerns	No concerns	**Low**
Placebo vs Synbiotic	Some concerns	Low risk	Some concerns	Major concerns	No concerns	No concerns	**Very low**
Placebo vs omega-3	No concerns	Low risk	No concerns	Major concerns	No concerns	No concerns	**Low**
Placebo vs VE	No concerns	Low risk	Some concerns	Some concerns	Some concerns	No concerns	**Low**
Placebo vs Green tea	No concerns	Low risk	No concerns	No concerns	No concerns	No concerns	**High**
Placebo vs Curcumin	No concerns	Low risk	No concerns	No concerns	Some concerns	No concerns	**Moderate**

Comparison	Within-study bias	Reporting bias	Indirectness	Imprecision	Heterogeneity	Incoherence	Overall confidence
			**TNF-α**				
Placebo vs Resveratrol	No concerns	Low risk	Some concerns	Major concerns	No concerns	No concerns	**Very low**
Placebo vs Mg	No concerns	Low risk	No concerns	Major concerns	No concerns	No concerns	**Low**
Placebo vs VD＋Mg	No concerns	Low risk	No concerns	Major concerns	No concerns	No concerns	**Low**
Placebo vs Zinc	No concerns	Low risk	Some concerns	Major concerns	No concerns	No concerns	**Very low**
Placebo vs omega-3+Probiotics	No concerns	Low risk	Some concerns	No concerns	No concerns	No concerns	**Moderate**
Placebo vs Probiotics	No concerns	Low risk	Some concerns	Major concerns	No concerns	No concerns	**Very low**
Placebo vs VD	No concerns	Low risk	No concerns	Major concerns	No concerns	No concerns	**Low**
Placebo vs α-lipoic acid	No concerns	Low risk	Some concerns	Major concerns	No concerns	No concerns	**Very low**
Placebo vs Synbiotic	No concerns	Low risk	Some concerns	Major concerns	No concerns	No concerns	**Very low**
Placebo vs omega-3	No concerns	Low risk	Some concerns	No concerns	No concerns	No concerns	**Moderate**
Placebo vs VE	No concerns	Low risk	Some concerns	Major concerns	No concerns	No concerns	**Very low**
Placebo vs Green tea	No concerns	Low risk	No concerns	Major concerns	No concerns	No concerns	**Low**
Placebo vs Curcumin	No concerns	Low risk	No concerns	Major concerns	No concerns	No concerns	**Low**

Comparison	Within-study bias	Reporting bias	Indirectness	Imprecision	Heterogeneity	Incoherence	Overall confidence
			CRP				
Placebo vs Resveratrol	No concerns	Low risk	Some concerns	Some concerns	Some concerns	No concerns	**Low**
Placebo vs Mg	No concerns	Low risk	No concerns	Some concerns	Some concerns	No concerns	**Moderate**
Placebo vs VD＋Mg	No concerns	Low risk	No concerns	No concerns	Major concerns	No concerns	**Low**
Placebo vs Zinc	No concerns	Low risk	Some concerns	No concerns	Some concerns	Some concerns	**Low**
Placebo vs α-lipoic acid + Probiotics	No concerns	Low risk	No concerns	Some concerns	Some concerns	No concerns	**Moderate**
Placebo vs omega-3+Probiotics	Some concerns	Low risk	Some concerns	No concerns	No concerns	No concerns	**Low**
Placebo vs Probiotics	No concerns	Low risk	Some concerns	No concerns	Some concerns	Some concerns	**Low**
Placebo vs VD	No concerns	Low risk	No concerns	Some concerns	Some concerns	Some concerns	**Moderate**
Placebo vs α-lipoic acid	No concerns	Low risk	No concerns	No concerns	Major concerns	No concerns	**Low**
Placebo vs Synbiotic	Some concerns	Low risk	Some concerns	No concerns	No concerns	No concerns	**Low**
Placebo vs omega-3	Some concerns	Low risk	No concerns	Some concerns	Some concerns	No concerns	**Low**
Placebo vs VE	No concerns	Low risk	Some concerns	No concerns	Major concerns	No concerns	**Very low**
Placebo vs Green tea	Some concerns	Low risk	No concerns	No concerns	Some concerns	No concerns	**Low**
Placebo vs Curcumin	Some concerns	Low risk	Some concerns	No concerns	No concerns	No concerns	**Low**

Comparison	Within-study bias	Reporting bias	Indirectness	Imprecision	Heterogeneity	Incoherence	Overall confidence
			**Leptin**				
Placebo vs Resveratrol	No concerns	Low risk	Some concerns	Major concerns	No concerns	No concerns	**Very low**
Placebo vs Mg	No concerns	Low risk	No concerns	Major concerns	No concerns	No concerns	**Low**
Placebo vs Zinc	No concerns	Low risk	Some concerns	Major concerns	No concerns	No concerns	**Very low**
Placebo vs Probiotics	No concerns	Low risk	No concerns	Major concerns	No concerns	No concerns	**Low**
Placebo vs VD	No concerns	Low risk	No concerns	Major concerns	No concerns	No concerns	**Low**
Placebo vs α-lipoic acid	No concerns	Low risk	No concerns	No concerns	Major concerns	No concerns	**Low**
Placebo vs omega-3	No concerns	Low risk	No concerns	Major concerns	No concerns	No concerns	**Low**
Placebo vs Green tea	No concerns	Low risk	No concerns	No concerns	No concerns	No concerns	**High**

Comparison	Within-study bias	Reporting bias	Indirectness	Imprecision	Heterogeneity	Incoherence	Overall confidence
			**Adiponectin**				
Placebo vs Resveratrol	No concerns	Low risk	Some concerns	No concerns	Major concerns	Major concerns	**Very low**
Placebo vs VD＋Mg	No concerns	Low risk	No concerns	No concerns	Major concerns	No concerns	**Low**
Placebo vs Probiotics	No concerns	Low risk	Some concerns	No concerns	Some concerns	Major concerns	**Very low**
Placebo vs VD	No concerns	Low risk	No concerns	No concerns	Major concerns	Major concerns	**Very low**
Placebo vs α-lipoic acid	No concerns	Low risk	No concerns	No concerns	Some concerns	No concerns	**Moderate**
Placebo vs Synbiotic	Some concerns	Low risk	No concerns	No concerns	No concerns	Major concerns	**Very low**
Placebo vs omega-3	Some concerns	Low risk	No concerns	No concerns	Some concerns	No concerns	**Low**
Placebo vs VE	No concerns	Low risk	Some concerns	No concerns	Major concerns	No concerns	**Very low**
Placebo vs Green tea	Some concerns	Low risk	No concerns	No concerns	Some concerns	Major concerns	**Very low**
Placebo vs Curcumin	No concerns	Low risk	No concerns	Some concerns	Some concerns	Major concerns	**Very low**
